# Direct access to a neutral alumene via CO reduction by a dialane and further CO homologation

**DOI:** 10.1038/s44160-025-00874-9

**Published:** 2025-09-16

**Authors:** John A. Kelly, Arseni Kostenko, Shigeyoshi Inoue

**Affiliations:** TUM School of Natural Sciences, Department of Chemistry, Catalysis Research Center and Institute of Silicon Chemistry, Technische Universität Müchen (TUM), Garching Bei München, Germany

**Keywords:** Chemical bonding, Chemical bonding

## Abstract

Multiple bonds between heavy elements have been shown to be not only stable but also offer divergent reactivity. Accordingly, there has been a drive in research to isolate such species. Here we report on the synthesis of a compound containing an aluminium–carbon double bond (alumene). The alumene was formed by exposing a dialane to a CO atmosphere. Experimental data and quantum chemical calculations confirm the existence of a *π*-bond between the aluminium and carbon centre. The mechanism for the formation of the alumene was calculated and indicated a heterocyclic intermediate, which we were able to observe spectroscopically. Treating the alumene with excess CO leads to CO homologation, forming a C_3_O_2_ chain initiated by interaction of a CO molecule with the *π*-bond of Al=C.

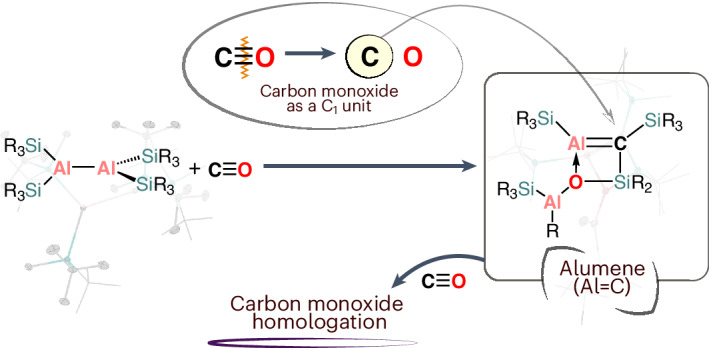

## Abstract (web summary)

Multiple bonds involving heavier elements were considered impossible but have recently been shown to be stable and offer divergent reactivity. Here the isolation of an alumene (a compound containing an Al=C bond) via direct CO reduction is described. Analysis of the alumene and its ability to homologate CO is reported.

## Main

**Fig. 1:**
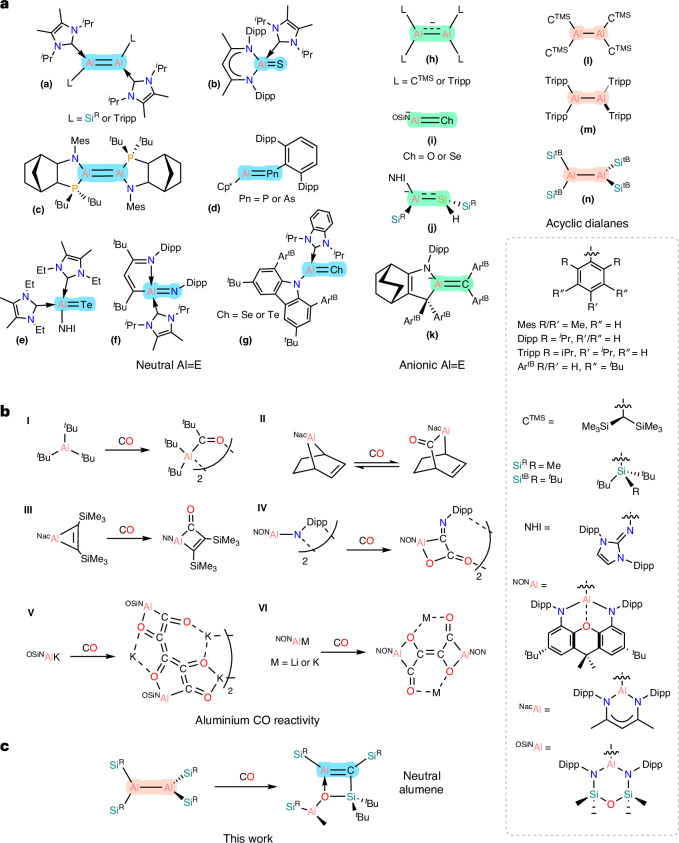
Aluminium multiple bonds and CO reactivity. **a**, Instances of Al=E multiple bonds (**a**–**k**) and acyclic dialanes (**l**–**n**) in the literature. **b**, Reported reactivity of aluminium compounds with CO (**I**–**VI**). **c**, The isolation of a neutral alumene (this work).

The ‘double-bond rule’ states that elements with principal quantum numbers higher than 2 cannot form multiple bonds; and it was thought that such compounds could not exist^1^. In spite of this, in 1976, Lappert and co-workers reported on the solid-state structure of the dimeric stannylene [^TMS^C_2_Sn]_2_, which appeared to have a weak Sn=Sn interaction (^TMS^C = (TMS)_2_CH, TMS = SiMe_3_)^2^. Then, in 1981, the isolation of a silene (TMS_2_Si=C(OTMS)Ad) by Brook et al., a disilene (Mes_2_Si=SiMes_2_) by West et al. and a diphosphene (Ar*P=PAr*) by Yoshifuji et al. were reported (Ad = adamantyl, Mes = 2,4,6-Me_3_-C_6_H_2_, Ar* = 2,4,6-^*t*^Bu_3_-C_6_H_2_)^3–5^. Since then, a wealth of multiply bonded compounds have been isolated, especially within the p-block^6–9^. One element that was notably lacking in multiply bonded compounds is aluminium. However, recently the isolation of dialumenes (Al=Al, **a**, **c**), iminoalanes (Al=N, **f**), pnictaalumenes (Al=P/As, **d**) and aluminium chalcogenides (Al=O/S/Se/Te, **b**, **e**, **g**) have been achieved (Fig. 1)^10–21^. Due to the vacant *p*-orbital on aluminium, it is possible to form anionic complexes with partial *π*-bonds, such as radical dialanes (**h**) and aluminata-silenes (**j**). It is also possible to form anionic heteronuclear double bonds, such as the recently published anionic alumene (Al=C) (**k**) and anionic aluminium chalcogenides (Al=O/Se/S/Te^−^, **i**) (Fig. 1).

Recently, work from our group has shown that using CO as a C1 source can lead to the isolation of heavy main-group multiply bonded compounds^22^. We reported that treating the silylene radical [(^*t*^Bu_3_Si)(TMS_3_Si)Si]^**·**^^−^ with CO results in a sila-ketenyl anion, [(^*t*^Bu_3_Si)Si=C=O]^−^. We were then interested in expanding this methodology to aluminium chemistry. Although a mainstay in transition metal chemistry, only recently has there been an emergence of main-group-mediated CO reactivity^23^. With regard to aluminium, there are few examples. Early reports include the insertion of CO into one of the Al–C bonds in ^*t*^Bu_3_Al and the incorporation of CO into metallobicycles (Fig. 1, **I**–**III**)^24–26^.

Utilizing CO as a building block in the synthesis of fine chemicals is very desirable and is the basis of the Fischer–Tropsch process^27^. A key aspect of this process is CO homologation, and recently it has been shown that low-oxidation-state main group compounds are capable of such reactivity^28^. In the case of aluminium there are reports on the CO homologation of transition metal carbonyl compounds, mediated by Al(I) compounds^29,30^. It has also been shown that aluminium imide, K[(NON)AlNAr], when treated with CO incorporates two molecules of CO to give K[(NON)Al(C_2_O_2_)NAr] (NON = 4,5-(DippN)_2_-2,7-^*t*^Bu-9,9-dimethylxanthene, Dipp = 2,6-^*i*^Pr_2_-C_6_H_3_) (Fig. 1, **IV**)^31^. An interesting aspect of this reaction is that the C≡O bond of one of the molecules of carbon monoxide has been completely cleaved, and has resulted in the formation of a C=N bond. The same system was shown to be able to incorporate multiple equivalents of CO to give conjugated C_4_ and C_6_ chains (Fig. 1, **V**)^32^. The anionic alumanyl, [(OSiN)AlK]_2_, can also couple molecular CO to give a C_5_ homologue (OSiN = O[Me_2_SiN(Dipp)]_2_) (Fig. 1, **VI**)^33^.

Recent advances in low-oxidation-state aluminium chemistry have mostly concerned the reactivity of anionic aluminium species, such as the alumanyl anions^34–36^. Our attention turned to neutral aluminium species especially dialanes, dimeric Al(II) compounds. Since the seminal work of Uhl, we have been lacking reports on dialane reactivity^37–45^. Due to the success in utilizing silyl-based ligands for the preparation of low-oxidation-state silicon species and the dialumene in our own research, we wanted to use silyl groups to isolate Al(II) compounds. A tetrasubstituted dialane was previously reported ((^*t*^Bu_3_Si)_4_Al_2_, **n**), stabilized using bulky ^*t*^Bu_3_Si ligands^46^. Due to this sterically hindering moiety, the Al–Al bond distance is exceptionally long, resulting in a compound of low stability due to facile Al–Al bond cleavage, making follow-up reactivity studies difficult. To remedy this, we proposed that using the less sterically hindering ^*t*^Bu_2_MeSi (Si^R^) would result in a more stable dialane. Initial attempts to form the desired bis-silyl iodo alane, (^*t*^Bu_2_MeSi)_2_AlI, by treating Na[^*t*^Bu_2_MeSi] with 0.5 equiv. of AlI_3_, resulted in the formation of the previously reported tris-silyl alane, (^*t*^Bu_2_MeSi)_3_Al, regardless of stoichiometry or reaction conditions^47^. Instead, we opted to go via an aluminium hydride species and subsequent halogenation to give the desired starting material, akin to how the precursor to the dialumenes are synthesized^10,11^.

## Synthesis of precursors

**Fig. 2:**
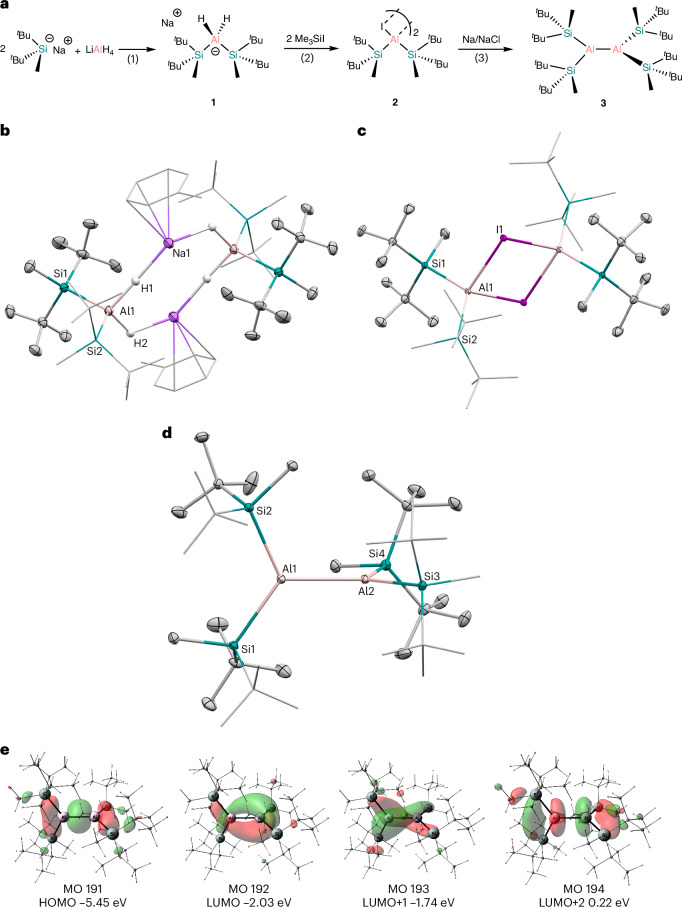
Synthesis of dialane 1. **a**, Synthesis of [(^*t*^Bu_2_MeSi)_2_AlH_2_Na]_2_, **1**, [(^*t*^Bu_2_MeSi)_2_Al(μ-I)]_2_, **2** and[(^*t*^Bu_2_MeSi)_2_Al]_2_, **3**.^a^ **b**–**d**, Thermal ellipsoid plots (30% probability surface) of **1** (**b**), **2** (**c**) and **3** (**d**). Hydrogen atoms (except H1 and H2 in **1**) are omitted for clarity. Selected bond lengths (Å) and angles (deg) for **1**: Al1–Si1 2.4873(8), Al1–Si2 2.4839(8), Al1–Na1 3.2529(11), Si1–Al1–Si2 120.92(3); for **2**: Al1–I1 2.7693(6), Al1–Si1 2.5236(9), Al1–Si2 2.5148(10), Si1–Al1–Si2 126.03(9), Si1–Al1–I1 113.36(5), Si2–Al1–I1 105.34(5); for **3**: Al1–Al2 2.6300(5), Al1–Si1 2.5284(4), Al1–Si2 2.5283(4), Al2–Si3 2.5252(4), Al2–Si4 2.5291(4), Si1–Al1–Si2 121.469(15), Si1–Al1–Al2 116.367(15), Si3–Al2–Si4 121.357(15). **e**, Selected molecular orbitals (MO) of **3** (isovalue = 0.04). ^a^Reagents and conditions: (1) diethyl ether/pentane, −78 °C to r.t., 16 h; (2) toluene, −78 °C to r.t., 16 h; (3) toluene, 60 °C, 16 h.

The reaction of 2 equiv. of Na[^*t*^Bu_2_MeSi] with LiAlH_4_ resulted in the clean formation of sodium aluminate, Na[Si^R^_2_AlH_2_] (**1**) (Fig. 2a). Compound **1** marks one of the few examples of a discrete substituted aluminate and the first bearing silyl ligands. Crystallization from toluene gives the toluene adduct (Fig. 2b). The molecular structure of **1** was elucidated using single-crystal X-ray diffraction (scXRD) and shows that **1** is dimeric, bridging via the hydrogen and sodium atoms. The central aluminium atoms are in a tetrahedral geometry ligated by two silyl groups and two hydride ligands. The Al1–Si1 (2.4873(8) Å) and Al1–Si2 (2.4839(8) Å) are close to identical to one another and are within a similar range to the previously reported silyl(halo)alane NHC adducts, (I′)Si^R^AlX_2_ (X = Br, Si–Al 2.474 Å; X = I, Si–Al 2.478 Å) (I′ = [MeCN(^*i*^Pr)]_2_C)^10^. The ^1^H NMR spectrum shows two singlets for the silyl moieties (*δ* = 0.26 ppm (Me), 1.18 ppm (^*t*^Bu)) and a broad hump for the aluminium-bound hydride ligands at *δ* = 1.72 ppm. The ^1^H NMR signal for the hydrides is upfield shifted compared with previously reported aluminates, (HMDS)_2_AlH_2_·Li and [PhCH_2_(^*t*^Bu)N]_2_AlH_2_·Li which range from 3.0 to 4.5 ppm (HMDS = (Me_3_Si)_2_N)^48,49^. The ^27^Al NMR spectrum has a broad peak at *δ* = 100.5 ppm, which is similar to the previously mentioned aluminates. No signal could be detected in the ^29^Si NMR spectrum, due to the quadrupolar nature of the aluminium atom to which the silyl ligands are directly bound.

Treating **1** with 2 equiv. of trimethylsilyl iodide (Me_3_SiI) in toluene forms the bis-silyl iodo alane, [Si^R^_2_Al(μ-I)]_2_ (**2**). Compound **2** is a rare example of a donor-free acyclic halo-alane with only a handful of examples, including (^*t*^Bu_3_Si)_2_AlCl, TMP_2_AlX (X = Cl or Br), HMDS_2_AlCl and [Dipp(SiR_3_)N]_2_AlI (R = Me or ^*i*^Pr) (TMP = tetramethylpiperidine)^50–53^. scXRD analysis shows that **2** exists as a dimer in the solid state, bridging via the iodide atoms. The Al–I bond distance (2.7693(6) Å) is one of the longest reported, exceeding other silyl-substituted iodo alanes (Fig. 2c)^10,54^. The Si–Al bond lengths in **2** (Al1–Si1 2.5236(9) Å, Al1–Si2 2.5148(10) Å) are elongated compared with those in **1** (vide supra). The geometry of the central aluminium centre in **2** is highly distorted tetrahedral with an obtuse Si1–Al1–Si2 bond angle (126.03(9)°), wider than that found in **1** (120.92(3)°). As in **1**, no signal could be detected in the ^29^Si NMR spectrum. It also was not possible to identify the signal for the central aluminium atom in the ^27^Al NMR spectrum, as is also the case for previously reported silyl-substituted iodo alanes^10,54^.

Next, we trialled different reducing agents in the transformation of **2** into the corresponding dialane and found that sodium on sodium chloride (Na/NaCl) was the most effective in terms of selectivity and yield. When stirring a solution of **2** and Na/NaCl in toluene at ambient temperature a deep red colour slowly forms over the course of 5 days. Upon work-up and recrystallization from *n*-pentane, deep red crystals of dialane, (Si^R^)_4_Al_2_ (**3**), were isolated. The reaction can be expedited by heating the solution to 60 °C, which results in the reaction being complete within 16 h without any appreciable decline in yield. Compound **3** is exceptionally stable in solution: heating a toluene solution up to 100 °C shows no signs of degradation, in stark contrast to (^*t*^Bu_3_Si)_4_Al_2_ which decomposes when heated to 50 °C. scXRD analysis of **3** shows two (^*t*^Bu_2_MeSi)_2_Al units bonded via a single Al–Al bond (Fig. 2d). The Al–Al bond length in **3** (2.6300(5) Å) is within the same range reported for acyclic trisubstituted dialanes (^TMS^C_4_Al_2_ = 2.660(1) Å, Tripp_4_Al_2_ = 2.647(3) Å, (^*t*^Bu_3_Si)_4_Al_2_ = 2.751(2) Å) (Tripp = 2,4,6-^*i*^Pr_3_-C_6_H_2_)^45,46,55^. The Si–Al–Al–Si torsion angle in **3** is almost perpendicular (82.8°), similar to (^*t*^Bu_3_Si)_4_Al_2_ (90°) but larger compared with both Tripp_4_Al_2_ (44.8°) and ^TMS^**C**_4_Al_2_ (8°). The Si–Al bond distances (Al–Si_ave_ 2.527(4) Å) and angles (Si–Al–Si = 121.4°) are similar to those in **1** and **2**.

To gain insight into the electronic structure of **3**, quantum chemical calculations were carried out (see Suppplementary Information for computational details). According to the natural bond orbital (NBO) analysis of the canonical molecular orbitals (CMOs), the HOMO of **3** is 96% bonding (Fig. 2e). The biggest contribution arises from the Al–Al bonding interaction (44%), while the four bonding Al–Si interactions sum to 37%. The LUMO and the LUMO + 1 are predominantly non-bonding (75% and 72%) and correspond mainly to the vacant *p*-orbitals of the two aluminium centres and can potentially act as electrophilic reactive sites (Fig. 2e). The antibonding σ*(Al–Al) is reflected in the antibonding (84%) LUMO + 2, where it is the largest contribution (33%), with additional high contributions from the vicinal σ*(Si–C) orbitals. The Al–Al bond in **3** has a Wiberg bond index (WBI) and Mayer bond order (MBO) of 0.93 and 1.01, respectively. The calculated Al–Al bond dissociation energy (BDE) is 60.5 kcal mol^−1^, which is very similar to the previously calculated Al–Al BDE in the parent system (H_2_Al–AlH_2_) of 58.8 kcal mol^−1^ (ref. ^56^). Additional computational results regarding bonding and properties of **3** can be found in Supplementary Figs. 38–41.

## CO reactivity

**Fig. 3:**
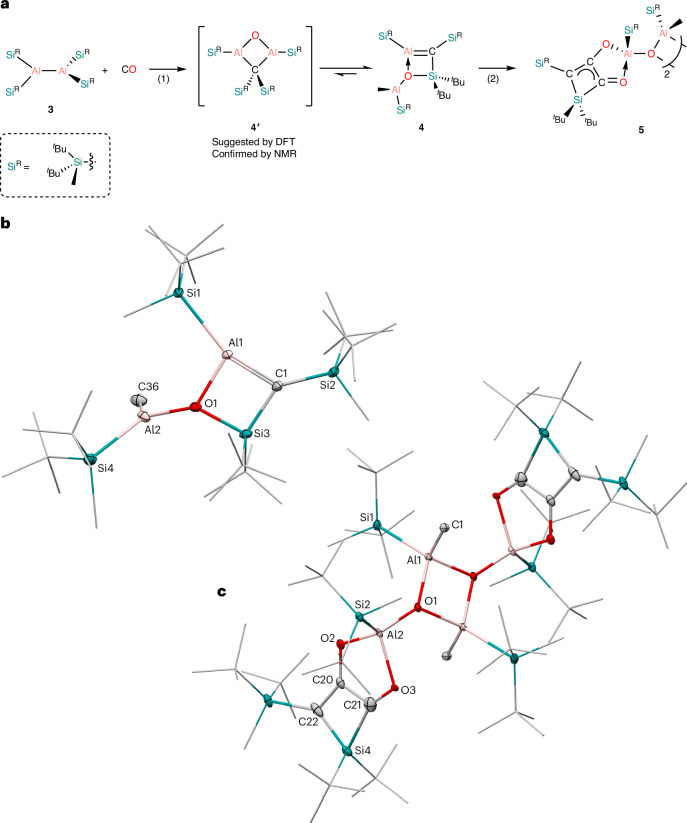
Synthesis of alumene 4. **a**, Synthesis of (Si^R^)C=Al(Si^R^)(^*t*^Bu_2_Si)OAlMe(Si^R^) **4** and homologation product, [(Si^R^)(^*t*^Bu_2_Si)C_3_O_2_Al(Si^R^)OAlMe(Si^R^)]_2_ **5**^a^. **b**,**c**, Thermal ellipsoid plots (20% probability surface) of **4** (**b**) and **5** (**c**). Hydrogen atoms are omitted for clarity. Selected bond lengths (Å) and angles (deg) for **4**: Al1–C1 1.849(3), Al1–Si1 2.4819(10), Al1–O1 1.917(2), C1–Si2 1.816(3), C1–Si3 1.792(3), Si3–O1 1.786(2), Al2–O1 1.820(3), Al2–C28 1.970(7), Al2–Si4 2.487(3), Si1–Al1–C1 160.35(9), O1–Al1–C1 89.17(10), Si1–Al1–O1 110.43(7), Al1–C1–Si2 133.64(15), Al1–C1–Si3 88.72(12), Si2–C1–Si3 137.55(15), Al1–O1–Si3 86.79(9), Si1–Al1–C1–Si2 7.9(7); for **5**: Al1–O1 1.979(2), Al1–O2 1.821(2), Al1–O3 1.7618(13), Al2–C21 1.968(3), Al2–O3 1.8381(15), C1–C2 1.383(5), C1–O1 1.262(3), C2–O2 1.317(7), C2–C3 1.383(5), C1–Si2 1.929(3), C3–Si2 1.915(5), C3–Si1 1.903(5), C1–C2–C3 105.5(5), O1–C1-C2 118.4(3), C1–C2–O2 115.9(4), C1–Si2–C3 71.88(16), Si2–C3–C2 52.5(3). ^a^Reagents and conditions: (1) benzene, r.t.; (2) 1 bar CO, benzene, 60 °C.

We then explored the potential reactivity of dialane **3** with CO. When putting **3** under 1 atm CO a colour change from red to yellow is observed within 5 min. After work-up in *n*-pentane and storing at −30 °C for 3–5 days, yellow crystals of alumene, (Si^R^)C=Al(Si^R^)(^*t*^Bu_2_Si)OAlMe(Si^R^) (**4**) were isolated (Fig. 3b). It appears that **3** has completely cleaved the C≡O bond and the carbon atom has formed a double bond with one of the aluminium centres. It also seems that silyl migration and activation has occurred with two of the silyl ligands. The Al1–C1 bond length (1.848(4) Å) is shorter compared with the standard Al–C bonds (1.95–2.01 Å) and the previously reported anionic **k** (1.869(3) Å)^19,57^. It should be noted that previous computational reports estimate that the Al=C bond length should be approximately 1.80 Å (refs. (^57,58^). The sum of the bond angles for A1 (359°) and C1 (358°), and the Si1–Al1–C1–Si2 torsion angle (7.8°), indicate that both the aluminium and carbon centres are in trigonal planar geometries. The Al1–O1 bond length (1.917(3) Å) is within the range of other ether alane adducts (for example, Ph_3_Al·OEt_2_; 1.920 Å (ref. ^59^)) and longer than Al2–O1 (1.779(3) Å) indicating the Al1–O1 bond is dative. When taking all the structural information together, there is strong evidence that there is a true multiple bond between the aluminium and carbon centre, giving an unprecedented, albeit base-stabilized, neutral alumene. The ^1^H NMR spectrum of **4** has eight singlet resonances, four for each ^*t*^Bu moiety and four for each methyl group, which are all inequivalent (Supplementary Fig. 18). The ^29^Si{^1^H} NMR spectrum of **4** shows only two silicon signals, the carbon-bound ^*t*^Bu_2_MeSi moiety (*δ* = 0.5 ppm) and the endocyclic ^*t*^Bu_2_Si group (*δ* = 45.5 ppm), the aluminium-bound silicon atoms are not visible due to the quadrupolar nature of ^27^Al. It was not possible to detect the alumene carbon (Al=*C*) in the ^13^C{^1^H} NMR spectrum using standard CO gas. However, when the reaction was conducted with labelled ^13^CO a peak at *δ* = 49.5 ppm was observed in the ^13^C{^1^H} NMR spectrum for the alumene carbon. Also, in the ^1^H NMR spectrum the signal at *δ* = 0.32 ppm is now a doublet, indicating ^3^*J*_CH_ coupling between the alumene carbon and the methyl group of the bound silyl ligand; this was also confirmed with a ^1^H–^13^C heteronuclear multiple bond correlation experiment (Supplementary Fig. 22). The alumene carbon signal for **4** is upfield shifted compared with that reported for the anionic **k** (*δ* = 70.1 ppm)^19^.

**Fig. 4:**
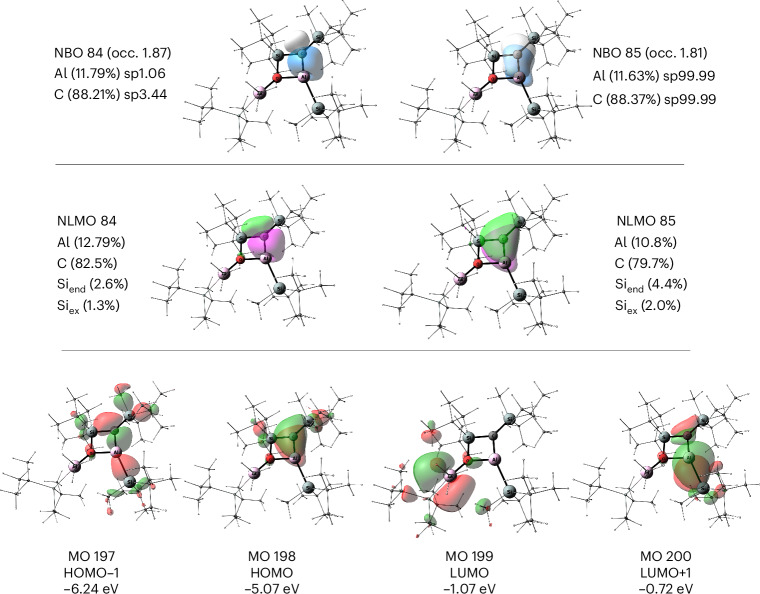
Bonding analysis of 4. Top: selected NBOs of (Si^R^)C=Al(Si^R^)(^*t*^Bu_2_Si)OAlMe(Si^R^) **4**. occ., occupancy. Middle: NLMOs of **4**. Bottom: selected canonical MOs of **4** (isovalue = 0.04). sp indicates hybridization degree.

NBO analysis shows two bonding interactions between the endocyclic aluminium and carbon centres (Fig. 4). The *σ*(Al–C) bond (NBO 84) which is strongly polarized towards the carbon centre (88% C, 12% Al) has an occupancy of 1.87*e*. According to the second-order perturbation theory, this is due to *σ*(Al–C) delocalization to the σ*(Si–O) and the σ*(Al–Si), with donor–acceptor interactions E(2) of 9.1 and 8.3 kcal mol^−1^, respectively. The *π*(Al–C) orbital (NBO 85) is also strongly polarized toward the carbon centre (88% C, 12% Al) and has a low occupancy of 1.81*e*. This is predominantly due to the donor–acceptor interactions with the two antibonding orbitals at the endocyclic silicon σ*(Si–C) with E(2) of 13.5 and 13.4 kcal mol^−1^. These delocalizations are reflected in the respective natural localized molecular orbitals (NLMOs) 84 and 85 (Fig. 4). Full NBO analysis of the bonding situation in the four-membered ring of **4** is presented in Supplementary Figs. 42 and 43). The WBI and the MBO for the Al–C bond in **4** are 0.70 and 1.46, respectively. Although the MBO clearly indicates a double-bonding nature of the Al–C interaction, the WBI is low, which could be due to the endocyclic O–Al interaction. To rationalize this observation, we looked at the hypothetical isomer of **4** in which the endocyclic O–Al interaction is cleaved. The open-chain isomer **4″** (Supplementary Fig. 46) can be obtained by rotation around the Si–C endocyclic bond. The results for this hypothetical isomer **4″**, which is 12.4 kcal mol^−1^ higher in energy than **4**, are similar to the cyclic system with WBI and MBO for the Al–C bond of 0.78 and 1.65. As with **4**, in **4″**, due to differences in electronegativity and charge separation, the NBOs corresponding to *σ*(Al–C) and *π*(Al–C) are strongly polarized toward the carbon centre (88% C, 12% Al, and 87% C, 13% Al), giving the Al–C interaction partial ionic character. Additionally, as in **4**, the *σ*(Al–C) and *π*(Al–C) of **4″** are substantially delocalized, in this case to the geminal Al–Si and the vicinal Si–C antibonding orbitals. Thus, the low WBI is not specific to the four-membered ring of **4** with the O–Al interaction but is due to the ionic character of the Al–C bond and the bonding density not being localized entirely on the Al–C fragment.

The Al–C multiple-bonding character of **4** is also reflected in the molecular orbitals (Fig. 4 and Supplementary Fig. 42). NBO analysis of the CMOs shows that the HOMO-1 of **4** is 93.5% bonding with the biggest contribution of the *σ*(Al–C) and additional contributions from *σ*(Al–Si) and the vicinal *σ*(Si–C) orbitals. The 89.5% bonding HOMO corresponds primarily to the *π*(Al–C) orbital. The LUMO is mainly non-bonding (64.8%) with the lone-vacancy *p*-orbitals of the exocyclic aluminium centre being the major components. The antibonding (86.3%) LUMO + 1 orbital is primarily the π*(Al–C). The intrinsic bond orbital (IBO) localization method also supports the assignment of a double-bond character to the Al–C interaction (Supplementary Fig. 45).

**Fig. 5:**
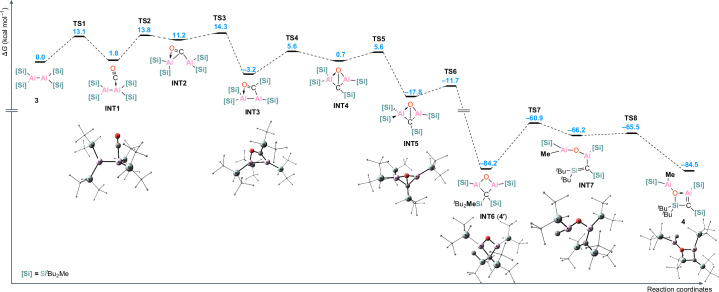
Mechanism for the formation of 4. Free energy reaction coordinate diagram for the proposed mechanism of the formation of (Si^R^)C=Al(Si^R^)(^*t*^Bu_2_Si)OAlMe(Si^R^) **4** from [(^*t*^Bu_2_MeSi)_2_Al]_2_ **3** in the presence of CO at the (SMD=Benzene)PW6B95-D4/def2-QZVPP//r^2^SCAN-3c level of theory.

We then examined the possible mechanism for the formation of **4** from **3** in the presence of CO, and initially set out to explore it computationally. The proposed mechanism for this transformation is presented in Fig. 5. Utilizing the LUMO which corresponds to the vacant *p*-orbital at the aluminium centre as an electrophilic site, dialane **3** reacts with one molecule of CO via **TS1** at 13.1 kcal mol^−1^ to form a dialane carbonyl complex, **INT1** at 1.8 kcal mol^−1^. The rearrangement of the carbonyl complex **INT1** via a transition state at 13.8 kcal mol^−1^ is a formal insertion of the CO into the Al–Al bond, which is accompanied by Al–Al bond cleavage, giving a dialane ketone intermediate **INT2** at 11.2 kcal mol^−1^. In **INT2** the oxygen atom of the carbonyl moiety is coordinated to an aluminium centre. A dialane ketone structural motif has been previously reported by our group; however, in that case the aluminium centres were coordinated by NHCs^60^. The formation of **INT2** enables the migration of one of the silyl ligands to the carbon centre of the CO moiety, forming **INT3** at −3.2 kcal mol^−1^. Thus, **INT3** represents a formal product of CO insertion into the Al–Si bond, in which the oxygen atom of the carbonyl coordinates to the second aluminium centre. This intermediate undergoes a rearrangement to form **INT4**, a species with a Al–C–Al–O four-membered ring. Such a structural motif has been reported several times in the literature^60–63^. The isomerization of **INT4** to **INT5** at −17.8 kcal mol^−1^ affords a conformer in which the migration of an additional Si^*t*^Bu_2_Me unit from the aluminium to the carbon is possible. Up until the formation of **INT5**, which is exergonic by −17.8 kcal mol^−1^, the barriers for all the steps have been low, with the highest transition state **TS3** at 14.3 kcal mol^−1^, which also corresponds to the highest barrier for the formation of **INT5**. The migration of the silyl group in **INT5** is highly exergonic, by 72.3 kcal mol^−1^, and gives the nearly C_2_-symmetric intermediate **INT6** (**4′**) at −84.2 kcal mol^−1^. In **INT6** (**4′**) the distance between an aluminium centre and a methyl carbon of a geminal ^*t*^Bu_2_MeSi substituent is only 2.621 Å, according to the calculations. This arrangement allows for the formation of the reactive silene intermediate **INT7**, which is endergonic by 18.8 kcal mol^−1^ and proceeds via a formal Al–C/Si–C bond metathesis. The process involves the methyl transfer from the silicon to the aluminium centre, accompanied by the ring opening by cleavage across the endocyclic Al–C bond, forming the silene **INT7**. The final step of the process is the addition of the oxygen to the silicon centre of the silene **INT7**, via a low barrier of only 0.7 kcal mol^−1^ to form an O–Si–C–Al four-membered cycle and yield the final product **4** at −84.5 kcal mol^−1^. The calculations suggest that the transformation of **INT6** (**4′**) to **4** is a nearly ergoneutral process which proceeds via a barrier of 23.3 kcal mol^−1^, which is much higher than the barriers of the preceding steps of the process, indicating it may be possible to observe **INT6** experimentally.

Based on the computational results we next monitored the initial reaction of **3** with CO via NMR spectroscopy to ascertain if **INT6** (**4′**) could be observed. This appears to be the case because upon addition of CO a new product is observed within 5 min in the ^1^H NMR spectrum as four singlet peaks. There are resonances at *δ* = 1.23 and 1.22 ppm for the ^*t*^Bu moieties and at 0.53 and 0.23 ppm for the methyl groups (Supplementary Fig. 25). The ^29^Si{^1^H} NMR spectrum shows a new peak at *δ* = 5.6 ppm, possibly indicating the migration of one of the silyl ligands away from an aluminium centre. We next conducted the reaction with ^13^CO. The ^1^H NMR spectrum of the reaction of **3** and ^13^CO is identical to that of the reaction with the non-labelled substrate except the peak at *δ* = 0.53 ppm is now a broad doublet. The ^13^C{^1^H} NMR spectrum shows a new signal at *δ* = 26.3 ppm, which is in agreement with the calculated shift at 26.2 ppm for the endocyclic carbon atom. Furthermore, the peaks at *δ* = 5.0 and 22.5 ppm which were previously singlets have now split into doublets, due to the proximity of the ^13^C to the silyl bound *C*H_3_ and *C*^*t*^Bu_3_ atoms. This splitting also occurs in the ^29^Si{^1^H} NMR spectrum. Any attempts to crystallize **4′** resulted in the isolation of **4**, presumably due to its higher crystallinity. Interestingly, when a solution of **4** is left standing, the peaks for **4′** start to appear in the ^1^H NMR spectrum, indicating an equilibrium, in a ratio of 5:1 (**4**:**4′**) (Supplementary Fig. 33). This is in line with the calculated mechanism, which predicts that the isomers **4** and **4′** are similar in energy (−84.2 and −84.5 kcal mol^−1^, respectively) and the barriers for their interconversion (**TS7** and **TS8**) should be achievable at ambient conditions (Fig. 5).

## CO homologation

Next, we wanted to see if the Al=C of **4** could react further, so we exposed a benzene solution of **4** to an atmosphere of CO and heated the reaction to 60 °C for 16 h, after which yellow crystals of the CO-coupled product, [(Si^R^)(^*t*^Bu_2_Si)C_3_O_2_Al(Si^R^)OAlMe(Si^R^)]_2_ (**5**) were isolated from the reaction solution (Fig. 3a). It should be noted that **5** can be formed directly from **3** under the same reaction conditions. scXRD analysis indicates that two molecules of CO have been coupled and inserted into the Al=C bond of **4**, forming an ethelenediolate-like moiety (C_2_O_2_) (Fig. 3c). The former alumene aluminium atom (Al=C) is now coordinatively saturated in **5** by three oxygen atoms and a silyl ligand. From the bond lengths, the aluminium centre is singly bonded to the silicon atom (Al2–Si2 2.5056(10) Å) and two of the oxygen atoms (Al2–O1 1.7606(18), Al2–O2 1.822(3) Å) and datively bonded to the third oxygen atom (1.981(3) Å). Within the C_2_O_2_ group it appears there is a single C–O (C20–O2 1.356(5) Å) and a double C=O bond (C21–O3 1.245(5) Å) and that it has formed a conjugated system with the alumene carbon atom (Al=C) in **4** (C20–C21 1.384(5), C20–C22 1.382(4) Å). The other aluminium centre (Al1) has not been altered from **4** to **5**, although now it is bridging via the oxygen atom (O1). Due to the insolubility of **5** it was not possible to obtain any NMR spectroscopic data. The formation of **5** is a rare instance of CO homologation by a neutral aluminium species utilizing CO gas.

**Fig. 6:**
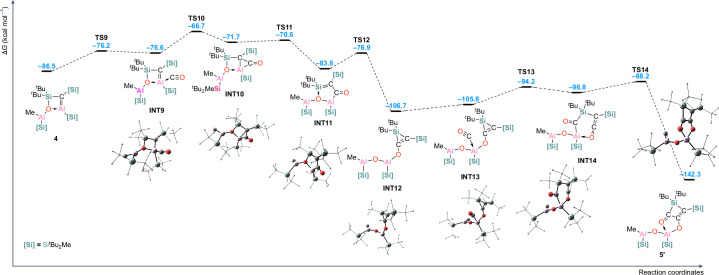
Mechanism for the formation of **5**. Free energy reaction coordinate diagram for the proposed mechanism of the formation of [(Si^R^)(^*t*^Bu_2_Si)C_3_O_2_Al(Si^R^)OAlMe(Si^R^)]_2_ **5** from (Si^R^)C=Al(Si^R^)(^*t*^Bu_2_Si)OAlMe(Si^R^) **4** in the presence of 2 equiv. CO at the (SMD=Benzene)PW6B95-D4/def2-QZVPP//r^2^SCAN-3c level of theory.

We were interested in the mechanism for the formation of **5**, and how the Al=C bond behaved in such a transformation. The calculated reaction coordinate of the proposed mechanism of this process is presented in Fig. 6. We propose that the initial step of the process is the addition of a CO molecule to the electrophilic site which corresponds to the antibonding Al–C π* orbital, that is, LUMO + 1 (Fig. 4), at the endocyclic aluminium centre. This step leads to the formation of intermediate **INT9** (−76.6 kcal mol^−1^) via the low barrier **TS9** of 10.3 kcal mol^−1^ in which the endocyclic Al–C bond is elongated by 0.040 Å, in comparison with **4**. This is followed by the formation of **INT10** (Al–C bond elongated by 0.040 Å in comparison with **4**)—a product of a formal [1 + 2] cycloaddition of CO into the Al=C double bond. This type of reactivity is well known for main-group multiple bonds. The Al–C bond is consequently cleaved by the insertion of the CO molecule forming the five-membered **INT11** (at Δ*G* = 2.7 kcal mol^−1^ relative to **4** + CO).

At this stage we propose that **INT11** rearranges to the three-membered silirene **INT12**, with an exocyclic R_2_Al–O–AlR–O– substituent, in an exergonic step of 22.9 kcal mol^−1^. An additional molecule of CO coordinates to an exocyclic Al(III) centre, without a barrier, forming **INT13**, which can undergo a ring expansion and form the six-membered **INT14**, via **TS13**. The consecutive C–C coupling via **TS14** forms the final product **5′**, which is a monomeric unit of the isolated compound **5**.

## Conclusion

We have reported on a neutral alumene, **4**, synthesized by the direct activation of CO by a dialane, **3**. scXRD and DFT analysis both support the existence of an aluminium–carbon double bond in **4**. This is another example of utilising CO as a C_1_ unit in the formation of intriguing multiply bonded carbon–main-group species (the other being the sila-ketenyl anion^22^). This reactivity also highlights the multifaceted silyl-based ligands that not only stabilize reactive compounds but, via their migration/elimination, can aid in the isolation of new and interesting species. Alumene **4** has been shown to react further with carbon monoxide, incorporating two more molecules of CO via the Al=C bond. Catenation with main-group elements, especially aluminium, is an emerging type of reactivity with promising applications. The calculated mechanism indicates the initial step involves the CO molecule interacting with the Al–C *π*-bond. This marks the first instance of reactivity of an aluminium–carbon multiple bond. We hope to expand this methodology to further develop low-oxidation-state/multiply bonded aluminium chemistry.

## Methods

Details of experimental procedures, analytical data, and X-ray structure determinations and computational procedures are given in the Supplementary Information.

### Synthetic methods

All reactions and product manipulations were carried out in flame-dried glassware under an inert atmosphere of argon using standard Schlenk-line or glovebox techniques (maintained at <0.1 ppm H_2_O and O_2_). Solvents were purified, dried and degassed with an MBraun SPS800 solvent purification system and then stored under argon over activated 3-Å molecular sieves or a potassium mirror in gas-tight ampoules. Deuterated benzene (C_6_D_6_) was obtained from Deutero Deutschland and was dried over 3-Å molecular sieves. Elemental analyses were conducted with a EURO EA (HEKA tech) instrument equipped with a CHNS combustion analyser at the Laboratory for Microanalysis at the TUM Catalysis Research Center. ^*t*^Bu_2_MeSiNa^64^ and Na/NaCl^65^ were prepared according to the literature. All other chemicals were used as purchased.

### Spectroscopic methods

All NMR samples were prepared under argon in J. Young PTFE tubes. NMR spectra were recorded on a Bruker AV400US. ^1^H and ^13^C NMR spectra were calibrated against the residual proton and natural abundance carbon resonances of the respective deuterated solvent as internal standard. Infrared spectra were recorded on a Perkin Elmer Spectrum Two Fourier transform–infrared spectrometer (diamond attenuated total reflectance) in the range 400–4,000 cm^−1^ at room temperature inside an argon-filled glovebox. Liquid injection field desorption ionization mass spectrometry was measured directly from an inert atmosphere glovebox with a Thermo Fisher Scientific Exactive Plus Orbitrap equipped with an ion source from Linden CMS.

### Crystallographic methods

Single-crystal diffraction data were collected on an X-ray single-crystal diffractometer equipped with an IMS microsource with Cu K_α_ and a Helios mirror optic by using the APEX III software package. The measurements were performed on single crystals coated with the perfluorinated ether Fomblin Y. The crystals were fixed on the top of a microsampler, transferred to the diffractometer and frozen under a stream of cold nitrogen. Additional details of the data processing, structure refinement and graphic depictions are given in the Supplementary Information.

### Computational methods

Calculations were carried out using ORCA 5.0.4 software. Geometry optimizations were carried using the r^2^SCAN-3c composite method, utilizing the regularized and restored SCAN functional, geometric counterpoise correction gCP, the atom-pairwise dispersion correction based on tight binding partial charges (D4), the def2-mTZVPP basis set and the def2-mTZVPP/J auxiliary basis set. Single-point calculations of the optimized geometries were carried out at the r^2^SCAN-3c level using the SMD solvation module to obtain electrostatic contribution and the cavity term in order to account for the solvent effects. Single-point calculations of the r^2^SCAN-3c optimized geometries were carried using the PW6B95 functional, with D4 dispersion correction, the def2-QZVPP basis set, and the def2/J and def2-QZVPP/C auxiliary basis sets. The method at which the free energies are reported is denoted as (SMD=Benzene)PW6B95-D4/def2-QZVPP//r^2^SCAN-3c. The NBO analysis was done using the NBO7 software, at the PBE0/def2-TZVP/r^2^SCAN-3c level of theory. Additional computational details are given in the Supplementary Information.

## Supplementary information

Supplementary Information Supplementary Figs. 1–51, Tables 1 and 2, Experimental details and Discussion.

Supplementary Data 1 Structure factors for 1: CCDC 2404617.

Supplementary Data 2 Structure factors for 2: CCDC 2404616.

Supplementary Data 3 Structure factors for 3: CCDC 2404620.

Supplementary Data 4 Structure factors for 4: CCDC 2404619.

Supplementary Data 5 Structure factors for 5: CCDC 2404618.

## Data Availability

All data generated or analysed during this study are included in this published Article and its Supplementary Information files. The structures of compounds **1**, **2**, **3**, **4** and **5** were determined by scXRD. Crystallographic data for the structures reported in this Article have been deposited at the Cambridge Crystallographic Data Centre, under deposition numbers CCDC 2404617 (**1**), 2404616 (**2**), 2404620 (**3**), 2404619 (**4**) and 2404618 (**5**). Copies of the data can be obtained free of charge via https://www.ccdc.cam.ac.uk/structures/. The Cartesian coordinates of all optimized and calculated structures, and the electronic energies, are summarized in the Supplementary Information. The file comprises all necessary data for reproducing the values. All non-default parameters for the computational studies are given in the Supplementary Information together with the corresponding references of the methods used. Further details are provided in the Supplementary Information.
