# Supplementary material for: Direct access to a neutral alumene via CO reduction by a dialane and further CO homologation
Source: Nat Synth. 2025 Sep 16;4(12):1577–86. doi: 10.1038/s44160-025-00874-9 (PMC12680539; doi:10.1038/s44160-025-00874-9)
Supplement: Supplementary file 1 — Supplementary Figs. 1–51, Tables 1 and 2, Experimental details and Discussion. [file 44160_2025_874_MOESM1_ESM.pdf]

nature synthesis

Supplementary information https://doi.org/10.1038/s44160-025-00874-9

Direct access to a neutral alumene via CO reduction by a dialane and further CO homologation

In the format provided by the authors and unedited

Supporting Information (72 pages)

Contents

**1. Synthesis**	**S2**
**2. X-Ray Crystallography**	**S6**
**3. NMR Spectroscopy**	**S13**
**4. UV-Vis Spectroscopy**	**S27**
**5. Mass Spectrometry**	**S28**
**6. IR Spectroscopy**	**S29**
**7. Quantum Chemical Calculations**	**S31**
**8. References**	**S72**

Synthesis

General Remarks

All reactions and product manipulations were carried out in flame-dried glassware under an inert atmosphere of argon using standard Schlenk-line or glovebox techniques (maintained at <0.1 ppm H_2O and O_2). Solvents were purified, dried, and degassed with an MBraun SPS800 solvent purification system and then stored under argon over activated 3 Å molecular sieves or a potassium mirror in gas-tight ampules. Deuterated benzene (C_6D_6) was obtained from Deutero Deutschland GmbH and were dried over 3 Å molecular sieves. All NMR samples were prepared under argon in J. Young PTFE tubes. NMR spectra were recorded on a Bruker AV400US. ^{1}H and ^{13}C NMR spectra were calibrated against the residual proton and natural abundance carbon resonances of the respective deuterated solvent as internal standard. Elemental analyses (EA) were conducted with a EURO EA (HEKA tech) instrument equipped with a CHNS combustion analyzer at the Laboratory for Microanalysis at the TUM Catalysis Research Center. Infrared (IR) spectra were recorded on a Perkin Elmer FT-IR spectrometer (diamond ATR, Spectrum Two) in a range of 400–4000 cm^{-1} at room temperature inside an argon-filled glovebox. Liquid Injection Field Desorption Ionization Mass Spectrometry (LIFDI-MS) was measured directly from an inert atmosphere glovebox with a Thermo Fisher Scientific Exactive Plus Orbitrap equipped with an ion source from Linden CMS. ^{t}Bu$_2$MeSiNa[1] and Na/NaCl[2] were prepared according to literature. All other chemicals were used as purchased.

[(^{t}Bu$_2$MeSi)$_2$AlH$_2$Na]$_2$ (1):

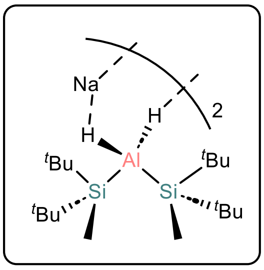

A solution of ^{t}Bu$_2$MeSiNa (2 g, 11.0 mmol) in *n*-pentane was added dropwise to a solution of $LiAlH_4$ (0.21 g, 5.5 mmol) in Et_2O at -78 °C. Upon complete addition, the reaction was left to stir and warm slowly overnight. Subsequently all volatiles were removed *in vacuo* and the residue was extracted with toluene. Again, all volatiles were removed *in vacuo* and *n*-pentane was added causing incipient precipitation of a white solid. This solid was isolated via filtration and dried under vacuum to yield **1** as a white crystalline powder. A second crop of **1** was obtained by concentrating the mother liquor and storing at -30 °C (1.55 g, 77%). X-ray quality crystals were grown from a concentrated toluene solution stored at -30 °C.

^{1}H NMR (400 MHz, C_6D_6, 298 K): δ/ppm = 0.27 (12H, s, SiCH_3), 1.18 (72H, s, C(CH_3)$_3$), 1.73 (4H, br m, AlH_2); ^{13}C{^{1}H} NMR (100 MHz, C_6D_6, 298 K): δ/ppm = -3.82 (4C, s, CH$_3$), 20.94 (8C, s, C(CH$_3$)$_3$), 30.74 (24C, s, C(CH$_3$)$_3$); ^{27}Al{^{1}H} NMR (100 MHz, C_6D_6, 298 K) δ/ppm = 100.5 (1Al, br, Si$_2$$AlH_2$)

Note: No signal was observed in the ^{29}Si{^{1}H} NMR spectrum.

[(^{t}Bu$_2$MeSi)$_2$Al(μ-I)]$_2$ (2):

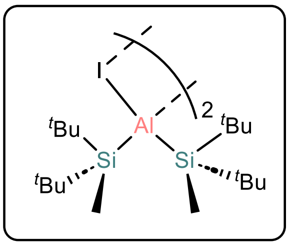

Neat Me_3SiI (0.79 mL, 5.54 mmol) was added dropwise to a solution of **1** (1 g, 1.26 mmol) in toluene cooled to -78 °C. Upon complete addition, the reaction was left to stir and warm slowly overnight. The reaction was then filtered and then all volatiles were removed *in vacuo* from the filtrate and *n*-pentane was added causing incipient precipitation of a white solid. This solid was isolated via filtration and dried under vacuum to yield **2** as a white crystalline powder (1.04 g, 82%). X-ray quality crystals were grown from a concentrated toluene solution stored at -30 °C.

^{1}H NMR (400 MHz, C_6D_6, 298 K): δ/ppm = 0.48 (12H, s, SiCH_3), 1.16 (72H, s, C(CH_3)$_3$); ^{13}C{^{1}H} NMR (100 MHz, C_6D_6, 298 K): δ/ppm = -4.74 (4C, s, CH$_3$), 22.44 (8C, s, C(CH$_3$)$_3$), 30.58 (24C, s, C(CH$_3$)$_3$)

Note: No signal was observed in the ^{29}Si{^{1}H} or ^{27}Al{^{1}H} NMR spectrum.

[(^{t}Bu$_2$MeSi)$_2$Al]$_2$ (3):

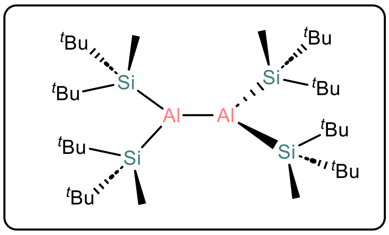

Compound **2** (1.00 g, 1.06 mmol) was added to a suspension of Na/NaCl (20% w/w, 0.61 g, 5.33 mmol) in toluene at ambient temperature, this was then heated to 60 °C overnight. All volatiles were removed, and the residue was extracted with *n*-pentane, the filtrate was then concentrated and stored at -30 °C. After one day crystals formed, which were isolated via filtration and dried under vacuum to yield **3** as a red crystalline powder. A second crop of **3** was isolated from the mother liquor (0.56 g, 77%).

UV-vis (c = 1.90 ·10^{-4} mol L^{-1}, 298K): λ_{max} = 357.0 nm, ε_{max} ≈ 2584 L mol^{-1} cm^{-1}; λ_{max} = 494.0 nm, ε_{max} ≈ 378 L mol^{-1} cm^{-1}

^{1}H NMR (400 MHz, C_6D_6, 298 K): δ/ppm = 0.52 (12H, s, SiCH_3), 1.15 (72H, s, C(CH_3)$_3$); ^{13}C{^{1}H} NMR (100 MHz, C_6D_6, 298 K): δ/ppm = -2.04 (4C, s, CH$_3$), 22.62 (8C, s, C(CH$_3$)$_3$), 31.23 (24C, s, C(CH$_3$)$_3$); ^{29}Si{^{1}H} (80 MHz, C6D6, 298 K): δ/ppm = 15.56 (4Si, s, R$_3$*Si*Al); ^{27}Al{^{1}H} NMR (100 MHz, C_6D_6, 298 K) δ/ppm = 140.17 (2Al, br, Si$_2$*AlAl*Si$_2$).

MS (ESI$^+$) *m/z* calcd: 682.53; m/z found: 682.5281. (**3**)

[(^{t}Bu$_2$MeSi)Al=C(^{t}Bu$_2$MeSi)Si(tBu)$_2$OAlMe(Si^{t}Bu$_2$Me)]$_2$ (4):

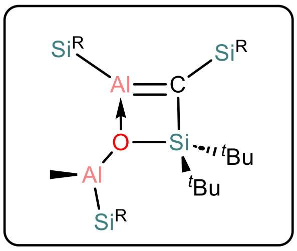

A degassed solution of **3** (0.20 g, 0.29 mmol) in benzene was pressured with CO/^{13}CO (1 bar) and left to stir for 16 hours. All volatiles were removed *in vacuo* and the residue was extracted with *n*-pentane, concentrated, and stored at -30 °C. After 3-4 days crystals formed which were isolated via filtration and dried under vacuum to yield **4** as a yellow crystalline powder. Further crops of **4** could be obtained by leaving the mother liquor at -30 °C (52 mg, 25%).

UV-vis (c = $5.34 \cdot 10^{-4}$ mol L^{-1}, 298K): λ_{max} = 359.0 nm, ε_{max} ≈ 2368 L mol^{-1} cm^{-1}

^{1}H NMR (400 MHz, C_6D_6, 298 K): δ/ppm = 0.06 (3H, s, SiC*H*$_3$), 0.16 (3H, s, SiC*H*$_3$), 0.28 (3H, s, SiC*H*$_3$), 0.32 (3H, s, SiC*H*$_3$), 1.05 (18H, s, C(C*H*$_3$)$_3$), 1.13 (18H, s, C(C*H*$_3$)$_3$), 1.30 (18H, s, C(C*H*$_3$)$_3$), 1.36 (18H, s, C(C*H*$_3$)$_3$); ^{13}C NMR (100 MHz, C_6D_6, 298 K): δ/ppm = -4.42 (s), -2.10 (s), -1.29 (s), 14.29 (s), 21.06 (s), 30.35 (br s), 31.30 (br s), 49.51 (s, *C*=Al); ^{29}Si{^{1}H} (100 MHz, C6D6, 298 K): δ/ppm = 0.52 (1Si, s), 45.59 (1Si, s) *Al-bound Si atoms not observed.*

MS (ESI$^+$) *m/z* calcd: 710.52; m/z found: 710.523. (**4**)

Note: No signal was observed in the ^{27}Al{^{1}H} NMR spectrum.

[(^{t}Bu$_2$MeSi)Al]$_2$OC(^{t}Bu$_2$MeSi)$_2$ (4')

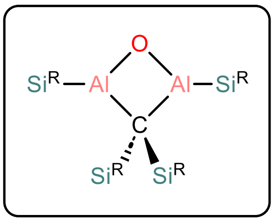

A degassed solution of **3** (20 mg, mmol) in C_6D_6 in a J Young NMR tube was pressured with CO/^{13}CO (1 bar) and its NMR spectrum was measured, revealing the formation of **4'**. Attempts to crystallise **4'** resulted in the isolation of **4**.

^{1}H NMR (400 MHz, C_6D_6, 298 K): δ/ppm = 0.23 (6H, s, SiC*H*$_3$), 0.53 (6H, s, SiC*H*$_3$), 1.23 (36H, s, C(C*H*$_3$)$_3$), 1.24 (36H, s, C(C*H*$_3$)$_3$); ^{13}C NMR (100 MHz, C_6D_6, 298 K): δ/ppm = -4.67 (s), 5.03 (s), 22.21 (s), 26.25 (s, OAl$_2$*C*), 30.36 (s), 32.37 (s); ^{29}Si{^{1}H} (100 MHz, C6D6, 298 K): δ/ppm = 5.64 (2Si, s, C*Si*$_2$), *Al-bound Si atoms not observed.*

Note: No signal was observed in the ^{27}Al{^{1}H} NMR spectrum.

[(Si^R)(^{t}Bu$_2$Si)C$_3$O$_2$Al(Si^R)OAlMe(Si^R)]$_2$ (5)

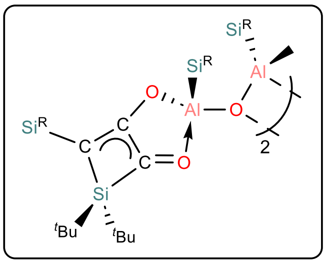

A degassed solution of **3** (0.1 g, 0.15 mmol) in benzene was pressured with CO (1 bar) and left to stir for 16 hours at 60 °C. Yellow crystals (suitable for XRD) were observed in the reaction solution which were isolated via filtration. (31 mg, 28%).

For (Mw: 1534.80 g mol^{-1}) C 61.04, H 11.03; found C 58.72, H 9.98.

The low value for carbon is explained by formation of incombustible silicon carbides.

IR (solid): ṽ [cm^{-1}] = 432 (w), 457 (w), 515 (m), 622 (m), 701 (m), 776 (w), 819 (m), 872 (w), 981 (m), 1148 (s), 1233 (s), 1389 (w), 1468 (m), 1561 (m), 1600 (m), 2855 (m), 2888 (m), 2931 (m).

Further IR discussion for 5 can be found in Section 6, page S29.

*Note: It was not possible to measure an NMR, mass or UV-vis spectrum due to the low solubility of **5**. It is insoluble in pentane, heptane, toluene, diethyl ether, THF and acetonitrile.*

2. X-Ray Crystallography

General Remarks

Either semi-empirical multi-scan absorption corrections or analytical ones were applied to the data.[3] Using Olex2,[4] the structures were solved with SHELXT[5] using intrinsic phasing and refined with SHELXL[6] using least-squares refinement on F^2.[7] The hydrogen atoms were located in idealized positions and refined isotropically with a riding model. Crystallographic data for the structures in this paper have been deposited with the Cambridge Crystallographic Data Centre, CCDC, 12 Union Road, Cambridge CB21EZ, UK. Copies of these data can be obtained free of charge (http://www.ccdc.cam.ac.uk) on quoting the depository number.

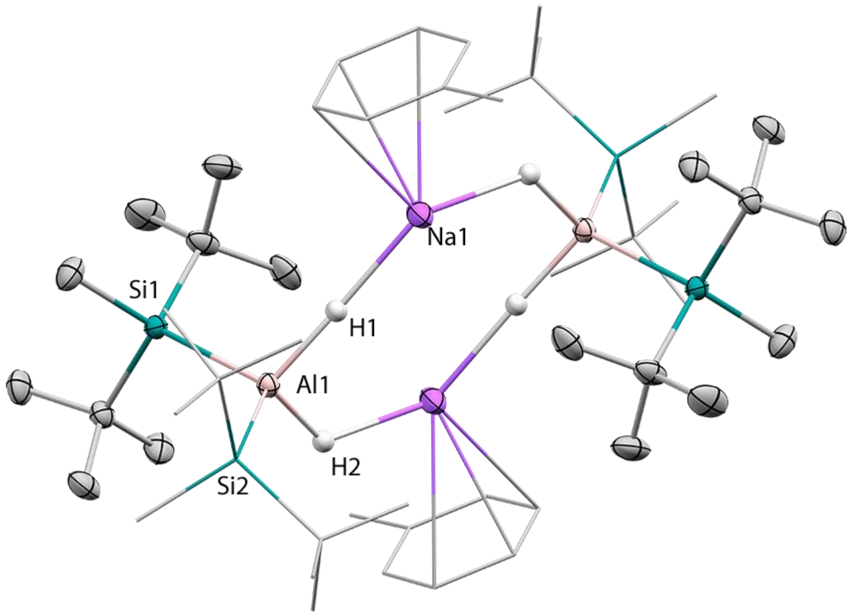

Figure S1. Thermal ellipsoid plots (30% probability surface) of [(tBu$_2$MeSi)$_2$AlH$_2$Na.toluene]$_2$ (**1**). Hydrogen atoms (except H and HA) are omitted for clarity. Selected bond lengths (Å) and angles (°): Al1-Si1 2.4874(8), Al1-Si2 2.4840(7), Si1-Al1-Si2 120.92(3).

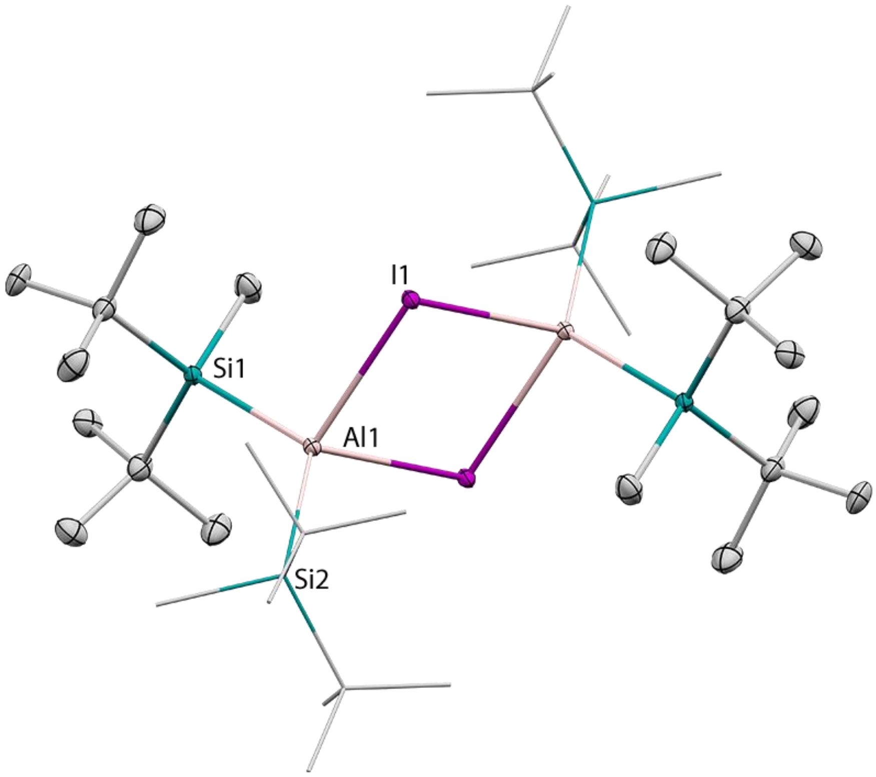

Figure S2. Thermal ellipsoid plots (30% probability surface) of $[(^{t}Bu_2MeSi)_2Al(\mu\text{-}I)]_2$ (**2**). Hydrogen atoms are omitted for clarity. Selected bond lengths (Å) and angles (°): Al1-I1 2.7692(5), Al1-Si1 2.5239(9), Al1-Si2 2.5150(6), Si1-Al1-Si2 126.02(2), Si1-Al1-I1 113.364(18), Si2-Al1-I1 105.335(17).

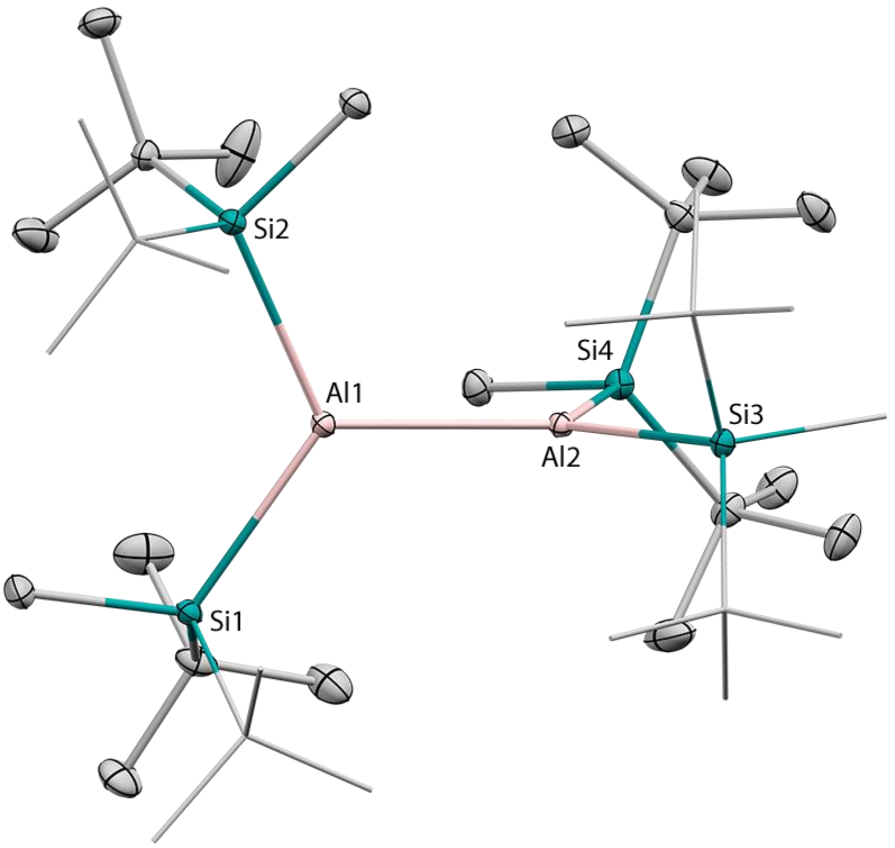

Figure S3. Thermal ellipsoid plots (30% probability surface) of (^{t}Bu$_2$MeSi)$_4$Al$_2$ (**3**). Hydrogen atoms are omitted for clarity. Selected bond lengths (Å) and angles (°): Al1-Al2 2.6300(4), Al1-Si1 2.5249(4), Al1-Si2 2.5292(3), Al2-Si3 2.5281(4), Al2-Si4 2.5284(4), Si1-Al1-Si2 121.359(12), Si1-Al1-Al2 116.591(12), Si3-Al2-Si4 121.474(12).

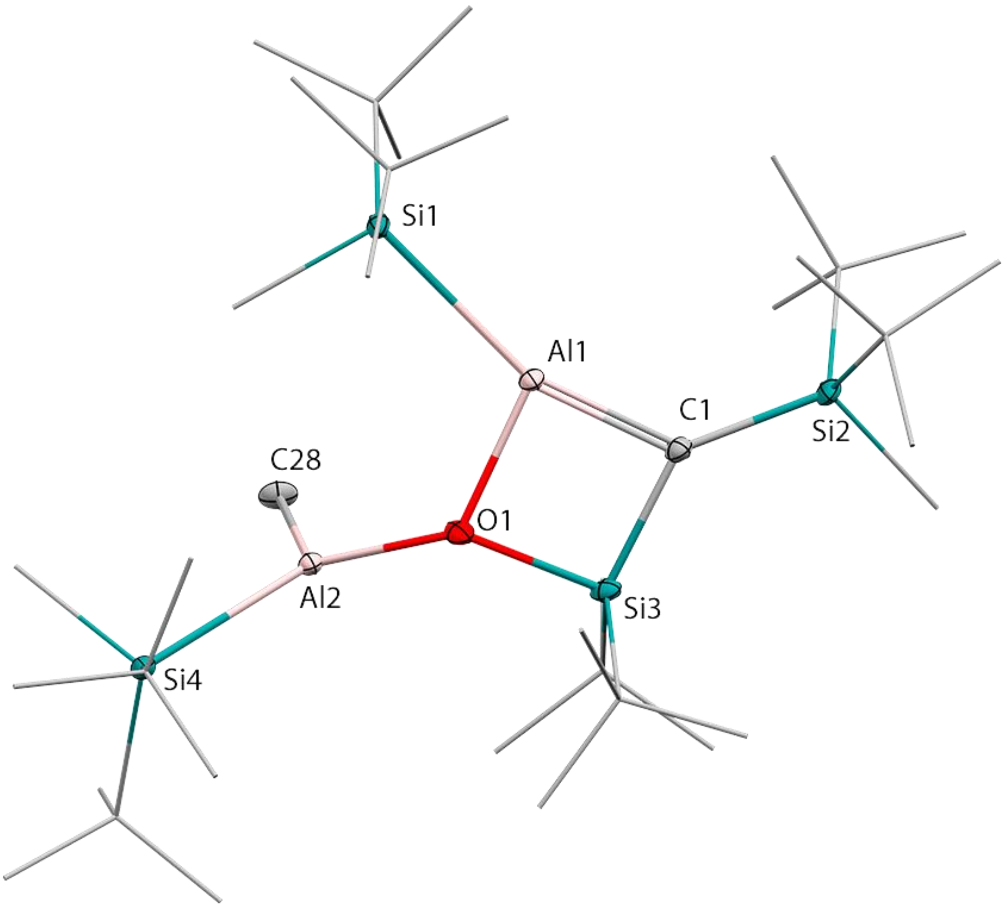

Figure S4. Thermal ellipsoid plots (20% probability surface) of *Si*Al=C*Si*[Si(*t*Bu)$_2$OAl(Me)*Si*] (**4**). Hydrogen atoms are omitted for clarity. Selected bond lengths (Å) and angles (°) for **4**: Al1-C1 1.849(3), Al1-Si1 2.4819(10), Al1-O1 1.917(2), C1-Si2 1.816(3), C1-Si3 1.792(3), Si3-O1 1.786(2), Al2-O1 1.820(3), Al2-C28 1.970(7), Al2-Si4 2.487(3), Si1-Al1-C1 160.35(9), O1-Al1-C1 89.17(10), Si1-Al1-O1 110.43(7), Al1-C1-Si2 133.64(15), Al1-C1-Si3 88.72(12), Si2-C1-Si3 137.55(15), Al1-O1-Si3 86.79(9), Si1-Al1-C1-Si2 7.9(7).

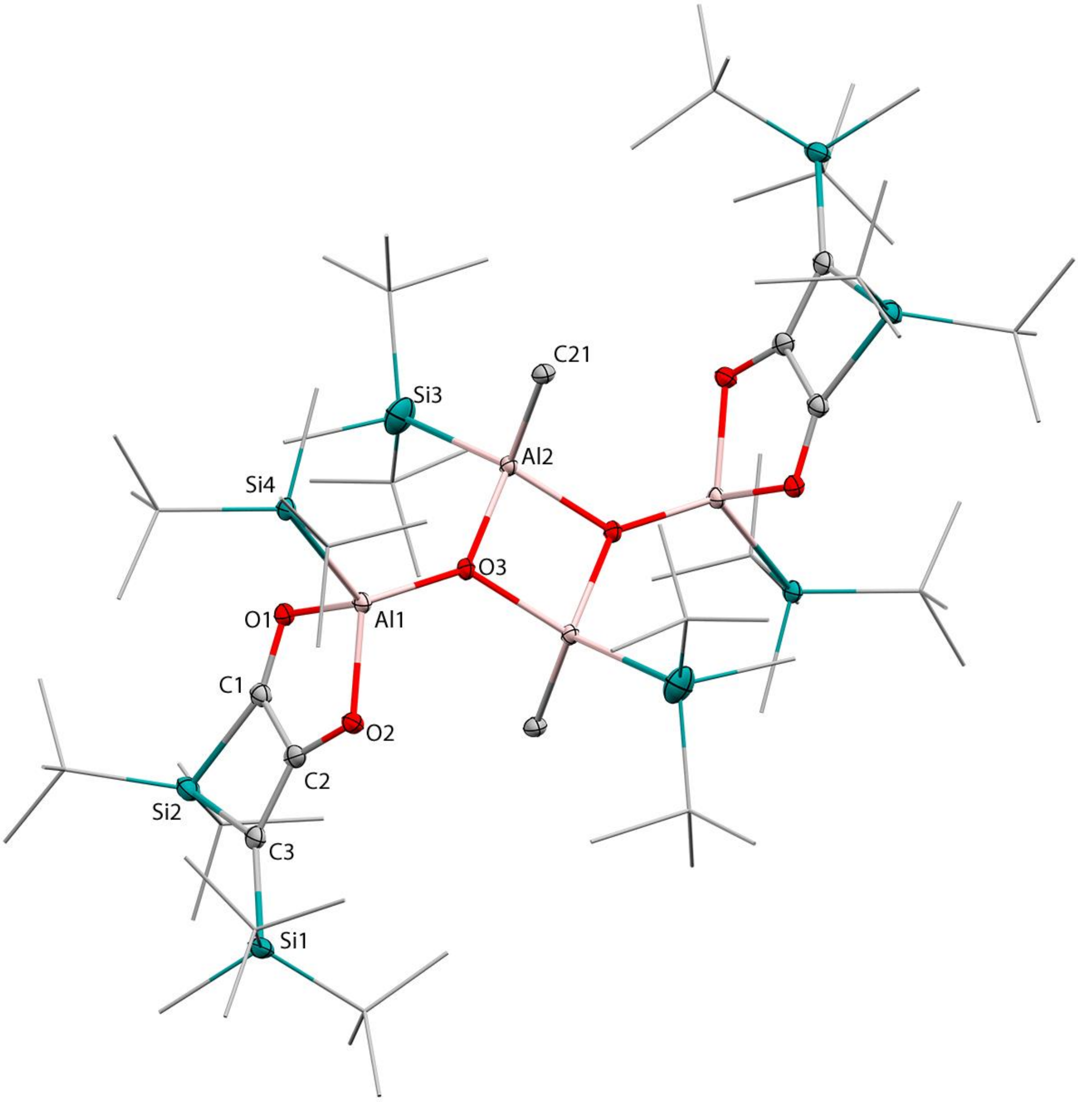

Figure S5. Thermal ellipsoid plots (20% probability surface) of (**5**). Hydrogen atoms are omitted for clarity. Selected bond lengths (Å) and angles (°) for **5**: Al1-O1 1.979(2), Al1-O2 1.821(2), Al1-O3 1.7618(13), Al2-C21 1.968(3), Al2-O3 1.8381(15), C1-C2 1.383(5), C1-O1 1.262(3), C2-O2 1.317(7), C2-C3 1.383(5), C1-Si2 1.929(3), C3-Si2 1.915(5), C3-Si1 1.903(5), C1-C2-C3 105.5(5), O1-C1-C2 118.4(3), C1-C2-O2 115.9(4), C1-Si2-C3 71.88(16), Si2-C3-C2 52.5(3).

Compound	**1**	**2**	**3**
Empirical Formula	$C_{50}H_{104}Al_2Na_2Si_4$	$C_{36}H_{84}Al_2I_2Si_4$	$C_{36}H_{84}Al_2Si_4$
CCDC Number	2404617	2404616	2404620
Formula Weight	917.63	937.15	683.35
Temperature [K]	100	100	100
Crystal System	monoclinic	triclinic	triclinic
Space Group	$P2_1/c$	P-1	P-1
a [å]	13.5207(15)	8.6926(9)	11.7212(11)
b [å]	11.5053(12)	11.7853(13)	12.9634(13)
c [å]	20.518(2)	12.9938(13)	17.1109(17)
α [°]	90	99.391(4)	73.928(4)
β [°]	108.246(4)	102.819(3)	89.585(4)
γ [°]	90	104.749(4)	67.036(4)
Volume [$Å^3$]	3031.3(6)	1220.7(2)	2285.4(4)
Z	2	1	2
P_{calc} [G/Cm^3]	1.005	1.275	0.993
μ [MM^{-1}]	0.17	1.444	0.189
F(000)	1016	488	764
Crystal Size [Mm^3]	0.1 × 0.07 × 0.05	0.07 × 0.05 × 0.02	0.05 × 0.05 × 0.01
Radiation	MoKα (λ = 0.71073)	MoKα (λ = 0.71073)	MoKα (λ = 0.71073)
2θ Range For Data Collection [°]	4.112 to 55.068	5.042 to 55.002	3.7 to 90.772
Index Ranges	-17 ≤ h ≤ 17, -14 ≤ k ≤ 14, -26 ≤ l ≤ 26	-11 ≤ h ≤ 11, -15 ≤ k ≤ 15, -16 ≤ l ≤ 16	-23 ≤ h ≤ 23, -25 ≤ k ≤ 25, -34 ≤ l ≤ 34
Reflections Collected	81116	78058	447308
Independent Reflections	6966 [R_{int} = 0.0441, R_{sigma} = 0.0246]	5603 [R_{int} = 0.0398, R_{sigma} = 0.0154]	38045 [R_{int} = 0.0846, R_{sigma} = 0.0433]
Data / Restraints / Parameters	6966/334/344	5603/80/297	38045/1044/525
Goodness-Of-Fit On F^2	1.162	1.081	1.127
Final R Indexes [I>=2σ (I)]	R_1 = 0.0584, wR_2 = 0.1315	R_1 = 0.0141, wR_2 = 0.0369	R_1 = 0.0510, wR_2 = 0.1232
Final R Indexes [All Data]	R_1 = 0.0724, wR_2 = 0.1516	R_1 = 0.0145, wR_2 = 0.0370	R_1 = 0.0818, wR_2 = 0.1395
Largest Diff. Peak/Hole [E $Å^{-3}$]	1.16/-0.42	0.66/-0.34	1.50/-0.60

Compound	**4**	**5**
Empirical Formula	$C_{37}H_{84}Al_2OSi_4$	$C_{78}H_{168}Al_4O_6Si_8$
CCDC Number	2404619	2404618
Formula Weight	711.48	1534.75
Temperature [K]	100	100
Crystal System	monoclinic	triclinic
Space Group	C2/c	P-1
a [å]	22.4648(13)	13.237(2)
b [å]	8.9967(4)	13.274(3)
c [å]	45.959(2)	14.308(2)
α [°]	90	73.701(8)
β [°]	98.381(4)	83.790(9)
γ [°]	90	79.456(9)
Volume [Å^3]	9189.6(8)	2367.9(7)
Z	8	1
P_{calc} [G/Cm^3]	1.029	1.076
μ [MM^{-1}]	0.192	0.194
F(000)	3169	848
Crystal Size [Mm^3]	0.06 × 0.02 × 0.01	0.07 × 0.04 × 0.03
Radiation	MoKα (λ = 0.71073)	MoKα (λ = 0.71073)
2θ Range For Data Collection [°]	3.84 to 52.28	4.188 to 58.622
Index Ranges	-27 ≤ h ≤ 27, -11 ≤ k ≤ 11, -56 ≤ l ≤ 56	-18 ≤ h ≤ 18, -18 ≤ k ≤ 18, -19 ≤ l ≤ 19
Reflections Collected	187517	135696
Independent Reflections	9124 [R_{int} = 0.0556, R_{sigma} = 0.0181]	12829 [R_{int} = 0.0578, R_{sigma} = 0.0298]
Data / Restraints / Parameters	9124/739/736	12829/1667/808
Goodness-Of-Fit On F^2	1.098	1.088
Final R Indexes [I>=2σ (I)]	R_1 = 0.0657, wR_2 = 0.1515	R_1 = 0.0610, wR_2 = 0.1586
Final R Indexes [All Data]	R_1 = 0.0703, wR_2 = 0.1547	R_1 = 0.0808, wR_2 = 0.1731
Largest Diff. Peak/Hole [E Å^{-3}]	0.66/-0.51	1.30/-0.56

3. NMR Spectroscopy

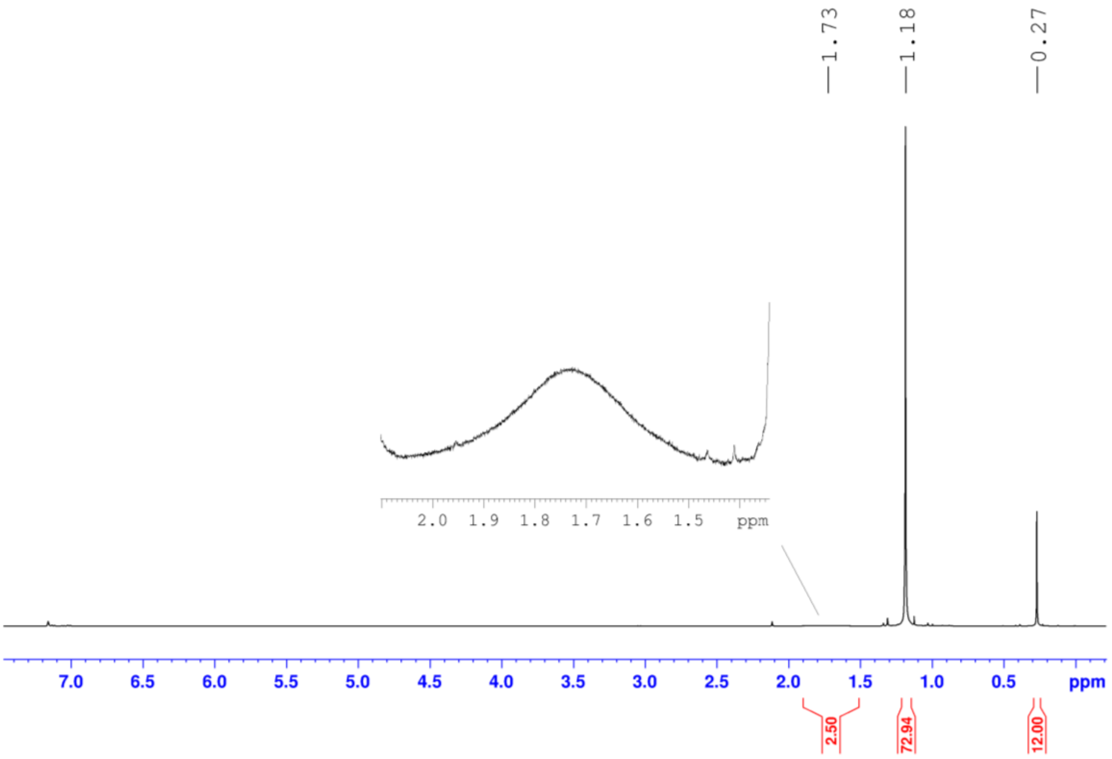

Figure S6. ^{1}H NMR Spectrum of (**1**) in C_6D_6 at ambient temperature.

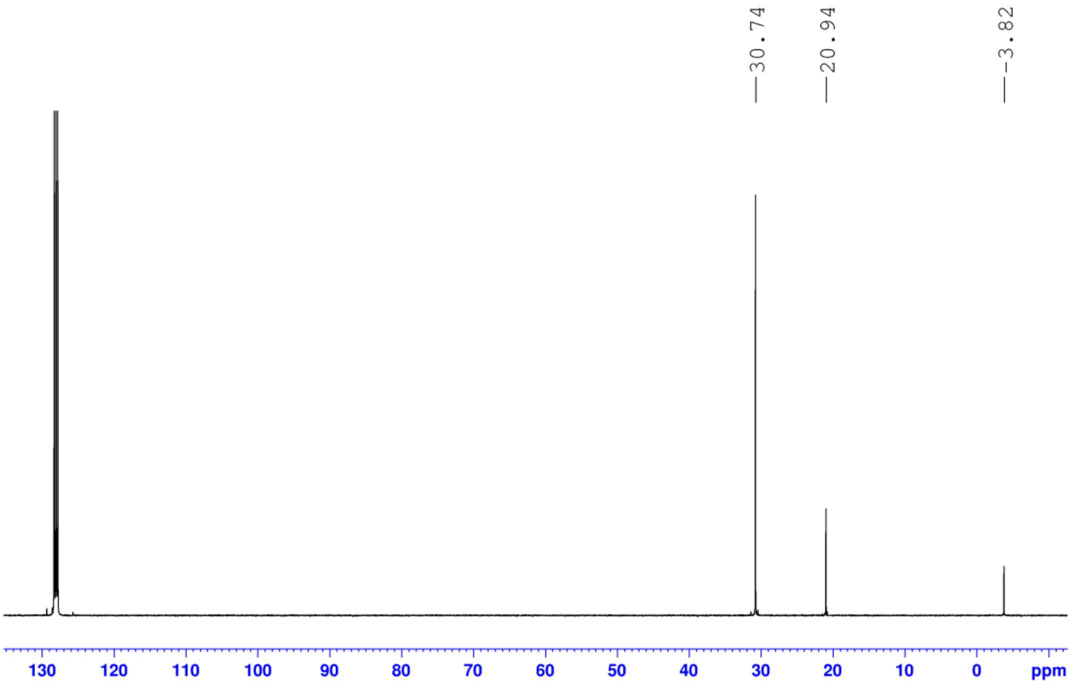

Figure S7. $^{13}C\{^1H\}$ NMR spectrum of **1** in C_6D_6 at ambient temperature.

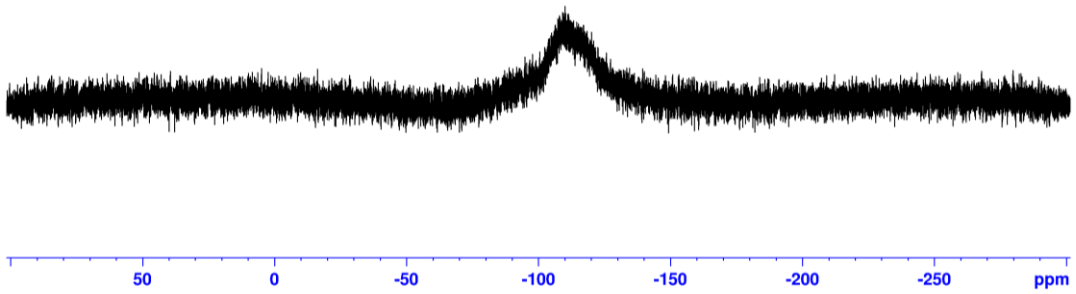

Figure S8. $^{29}Si\{^1H\}$ NMR spectrum of **1** in C_6D_6 at ambient temperature.

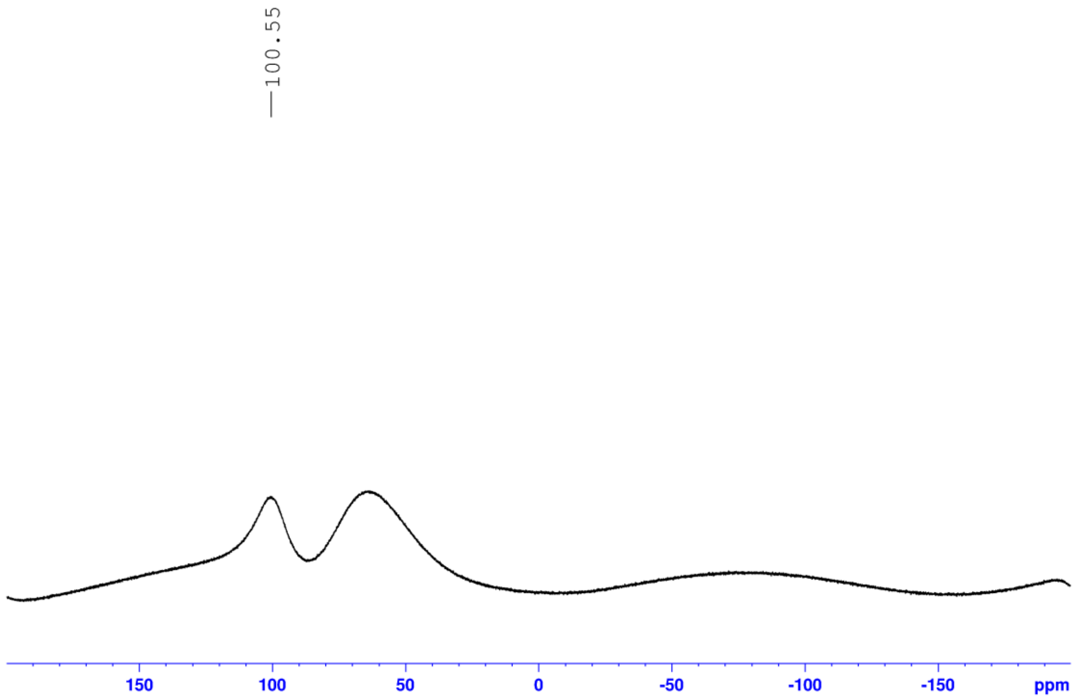

Figure S9. $^{27}Al\{^1H\}$ NMR spectrum of **1** in C_6D_6 at ambient temperature.

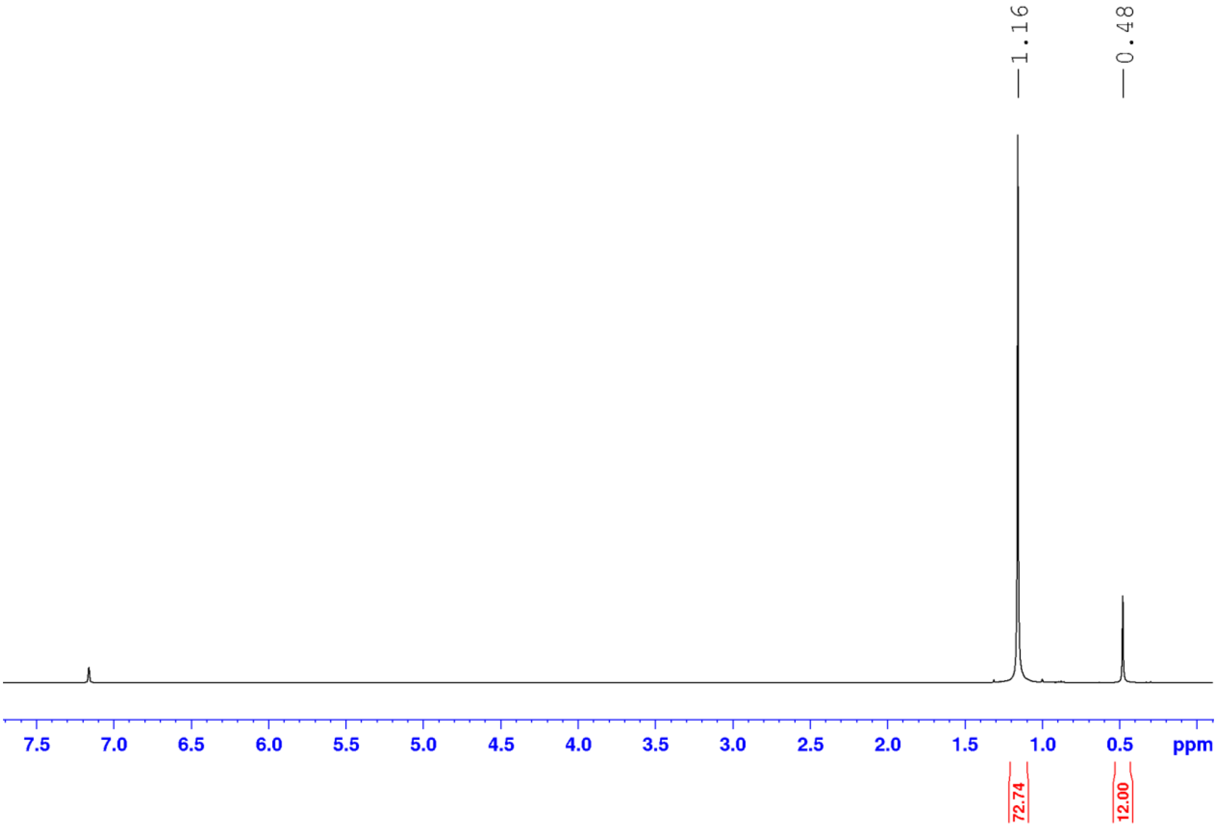

Figure S10. ^{1}H NMR Spectrum of (**2**) in C_6D_6 at ambient temperature.

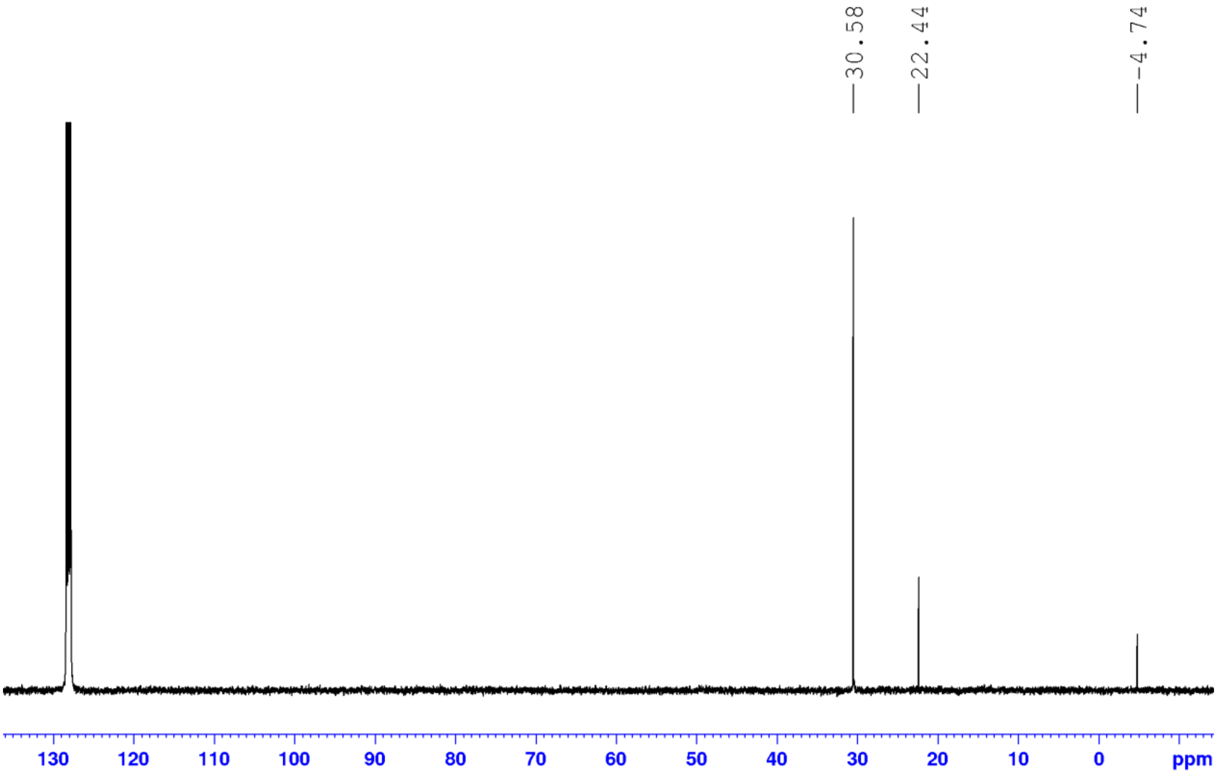

Figure S11. $^{13}C\{^1H\}$ NMR spectrum of **2** in C_6D_6 at ambient temperature.

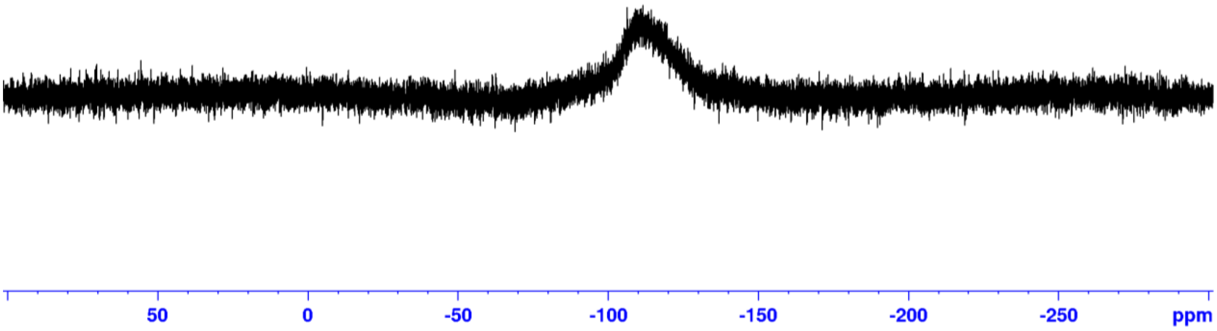

Figure S12. $^{29}Si\{^{1}H\}$ NMR spectrum of **2** in C_6D_6 at ambient temperature.

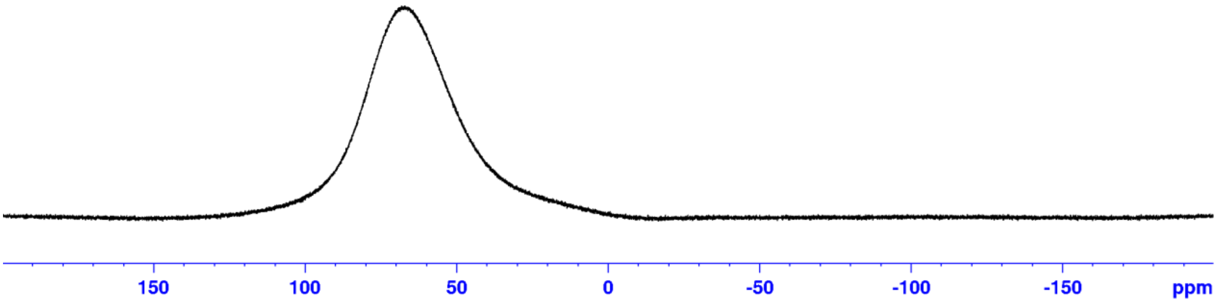

Figure S13. ^{27}Al NMR spectrum of **2** in C_6D_6 at ambient temperature.

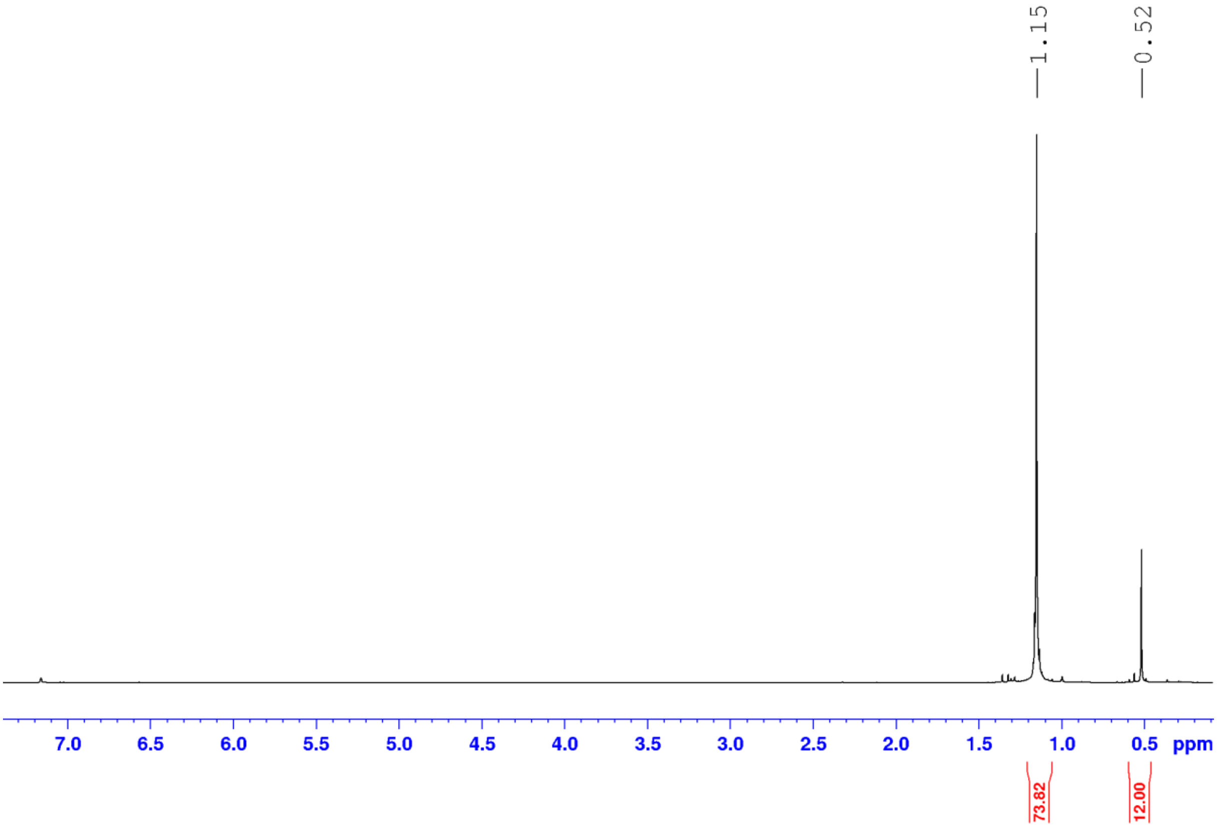

Figure S14. ^{1}H NMR Spectrum of (**3**) in C_6D_6 at ambient temperature.

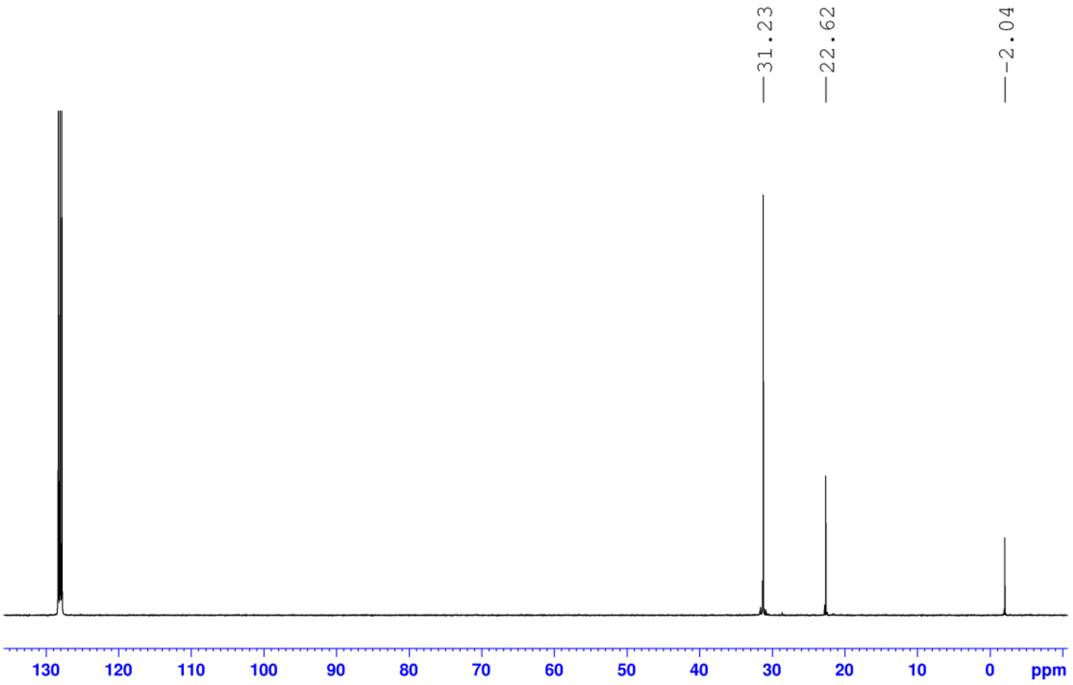

Figure S15. ^{13}C{^{1}H} NMR spectrum of **3** in C_6D_6 at ambient temperature.

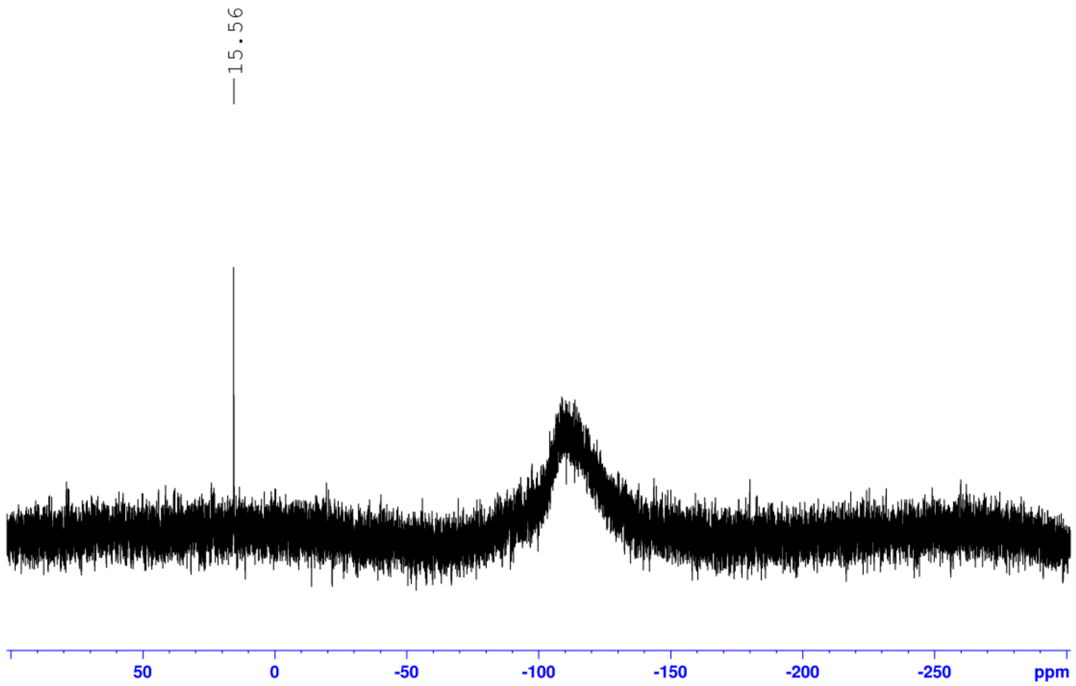

Figure S16. $^{29}Si\{^{1}H\}$ NMR spectrum of **3** in C_6D_6 at ambient temperature.

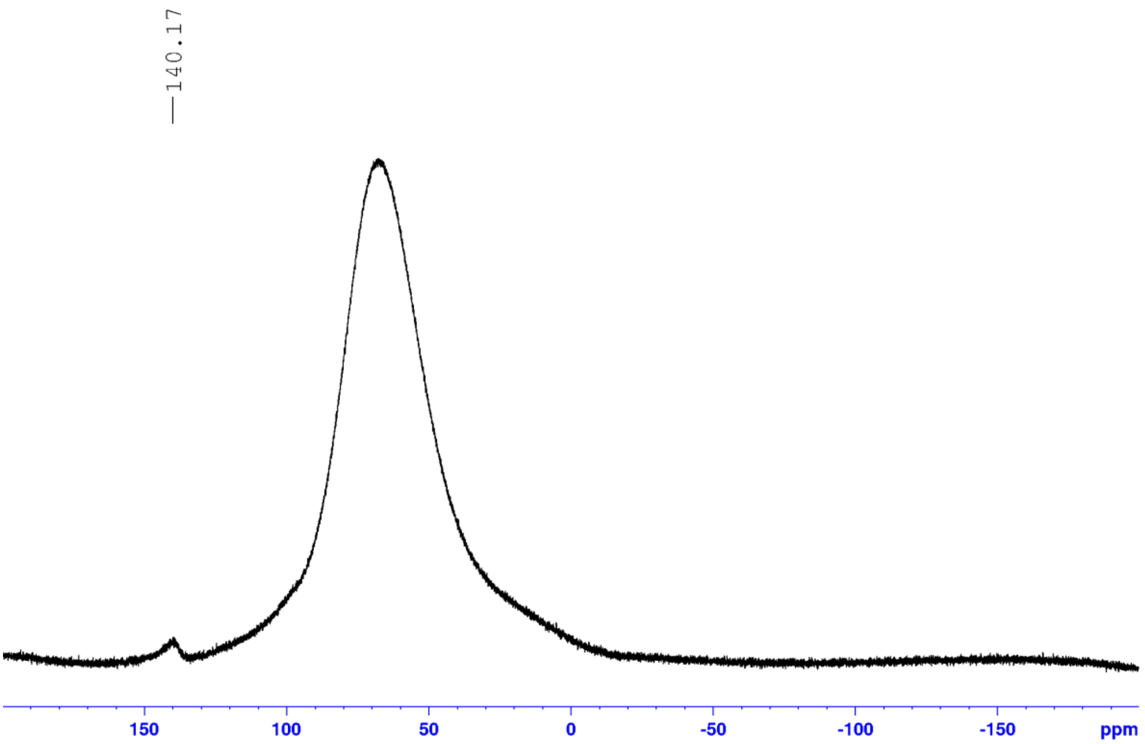

Figure S17. ^{27}Al NMR spectrum of **3** in C_6D_6 at ambient temperature.

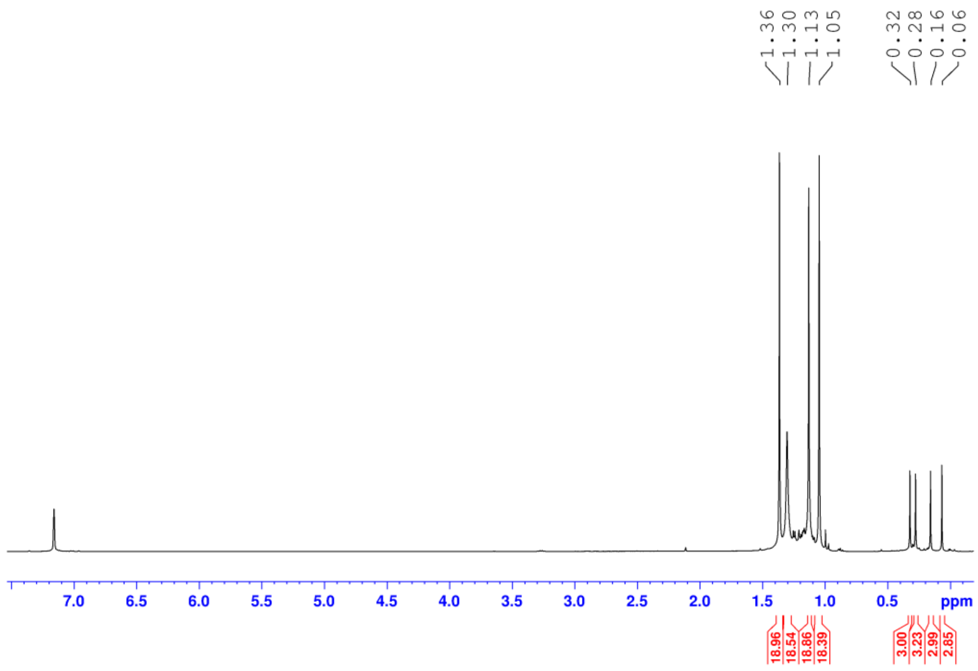

Figure S18. ^{1}H NMR Spectrum of (**4**) in C_6D_6 at ambient temperature.

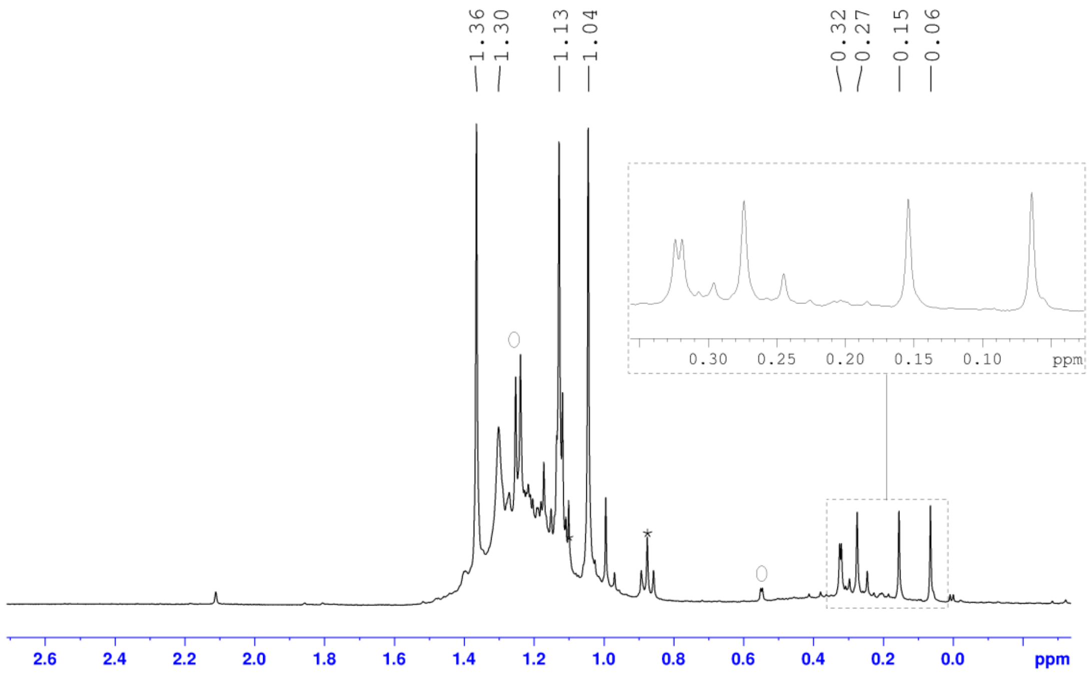

Figure S19. ^{1}H NMR Spectrum of (**4**) in C_6D_6 (from ^{13}CO) within the range -0.1 – 2.7 ppm at ambient temperature. (* = OEt_2, ○ = **4’**).

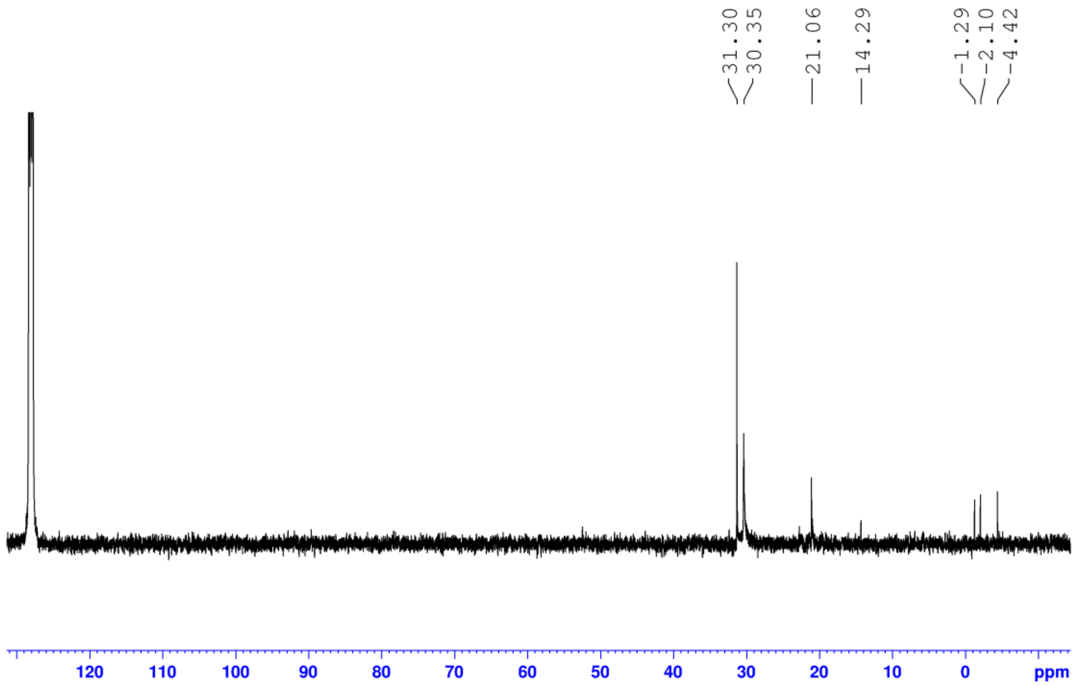

Figure S20. $^{13}C\{^{1}H\}$ NMR spectrum of **4** in C_6D_6 at ambient temperature.

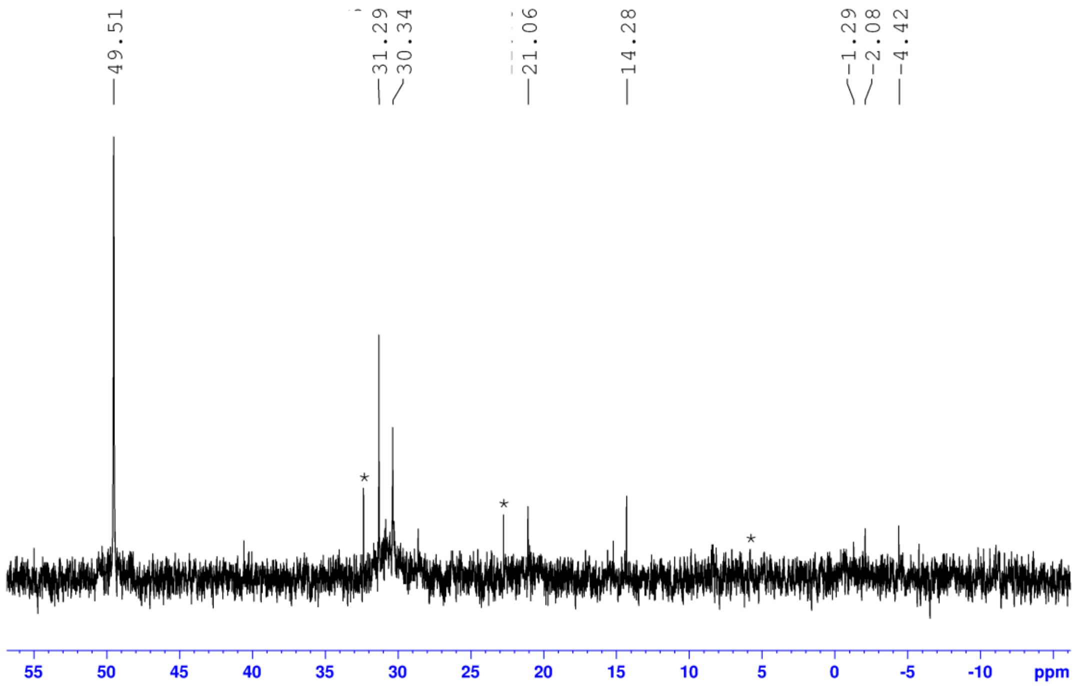

Figure S21. ^{13}C NMR Spectrum of (**4**) in C_6D_6 (from ^{13}CO) within the range -15 – 55 ppm at ambient temperature. (* = **4'**).

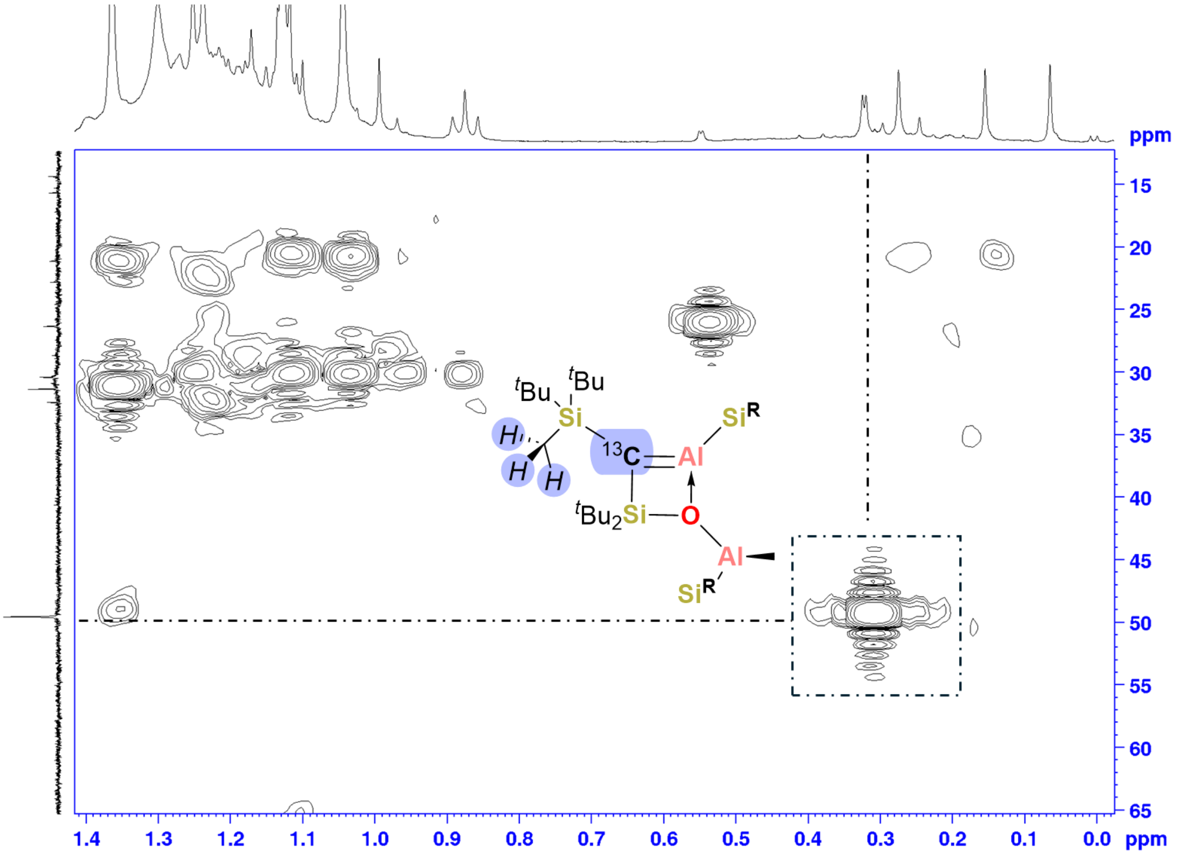

Figure S22. HMBC (^{1}H-^{13}C) NMR spectrum of **4** (from ^{13}CO), within the range 1.4 – 0/10 – 65 ppm highlighting the alumene carbon coupling to the methyl group of the silyl ligand. (^{1}H NMR spectrum = top/ ^{13}C NMR spectrum = left)

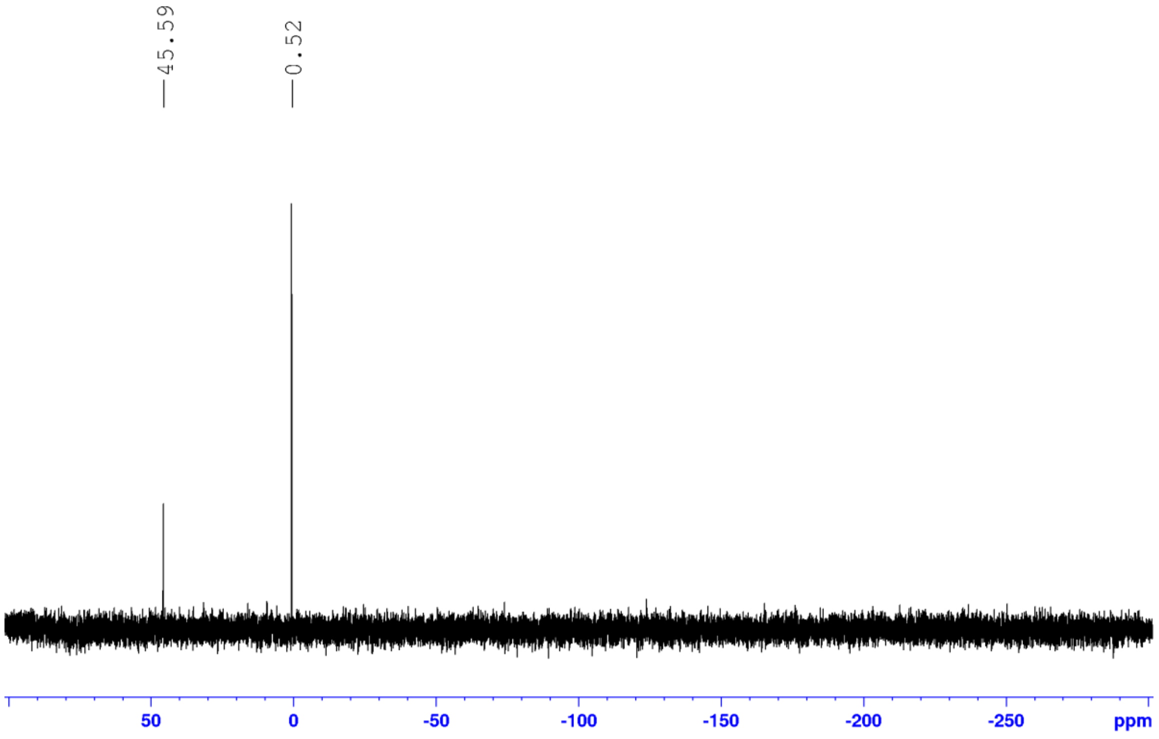

Figure S23. $^{29}Si\{^{1}H\}$ NMR spectrum of **4** in C_6D_6 at ambient temperature.

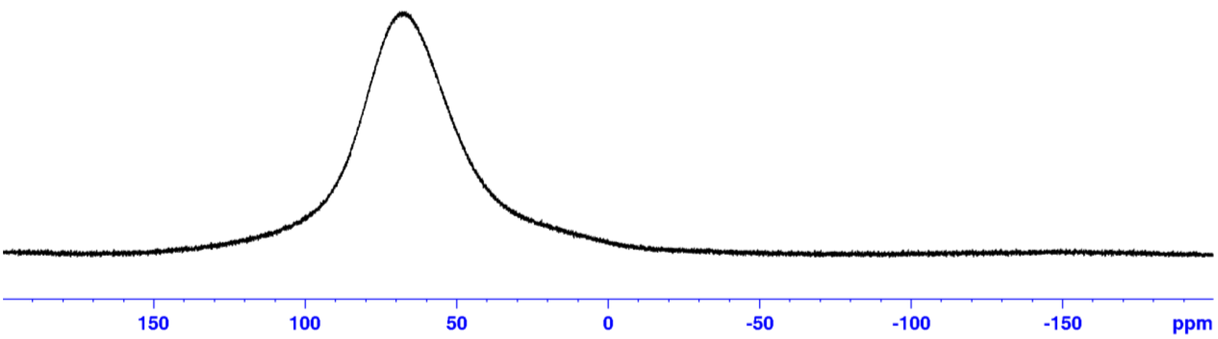

Figure S24. ^{27}Al NMR spectrum of **4** in C_6D_6 at ambient temperature.

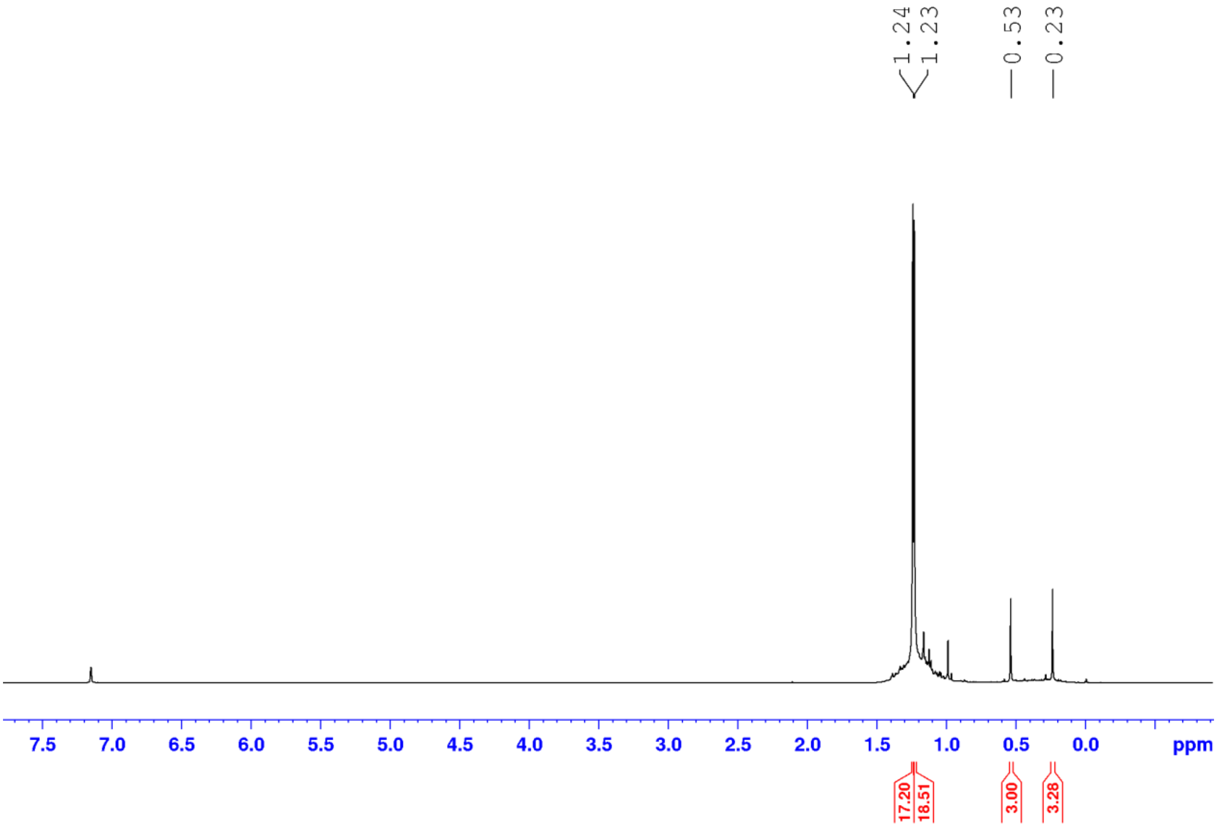

Figure S25. ^{1}H NMR Spectrum of **4’** in C_6D_6 at ambient temperature.

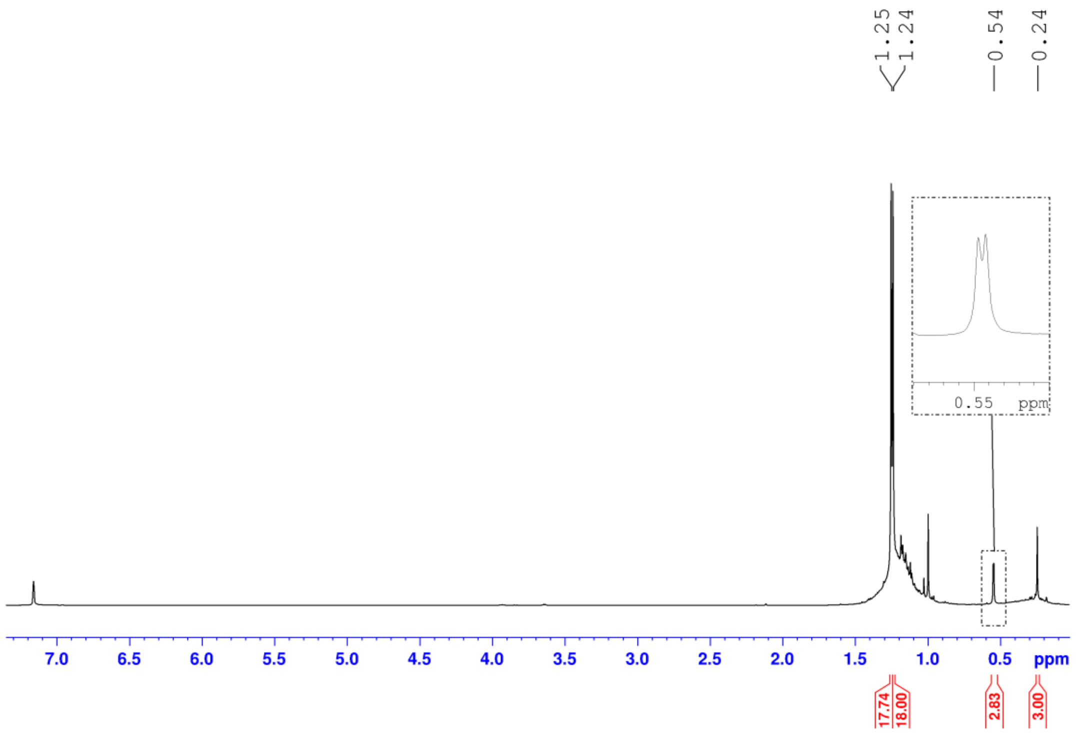

Figure S26. ^{1}H NMR Spectrum of **4'** (from ^{13}CO) in C_6D_6 at ambient temperature.

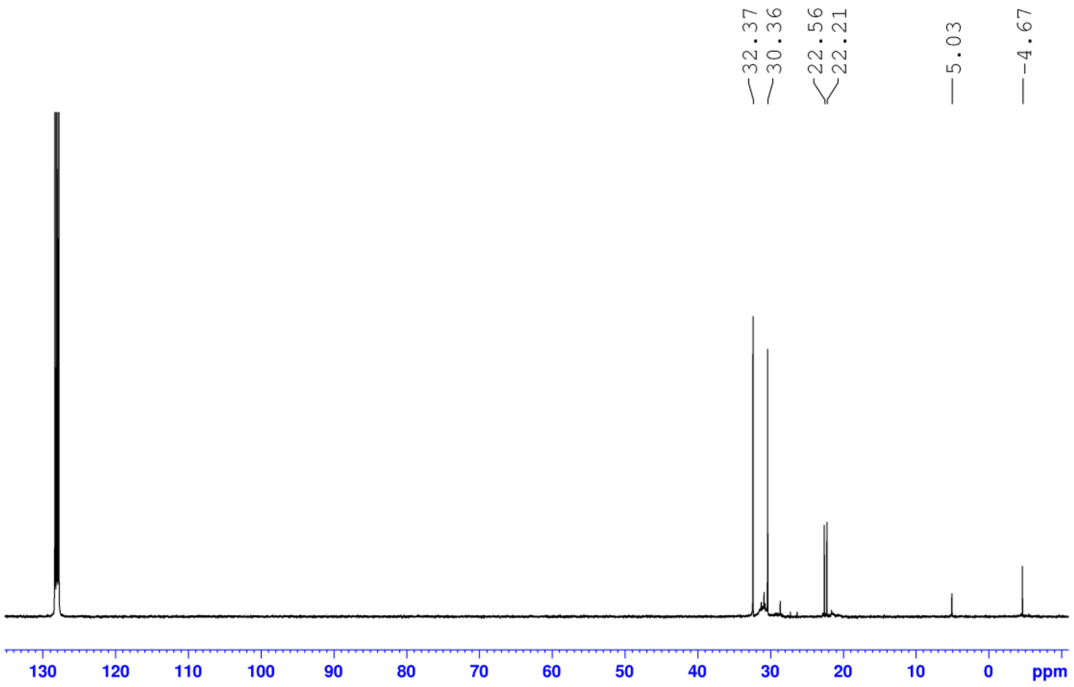

Figure S27. $^{13}C\{^{1}H\}$ NMR spectrum of **4'** in C_6D_6 at ambient temperature.

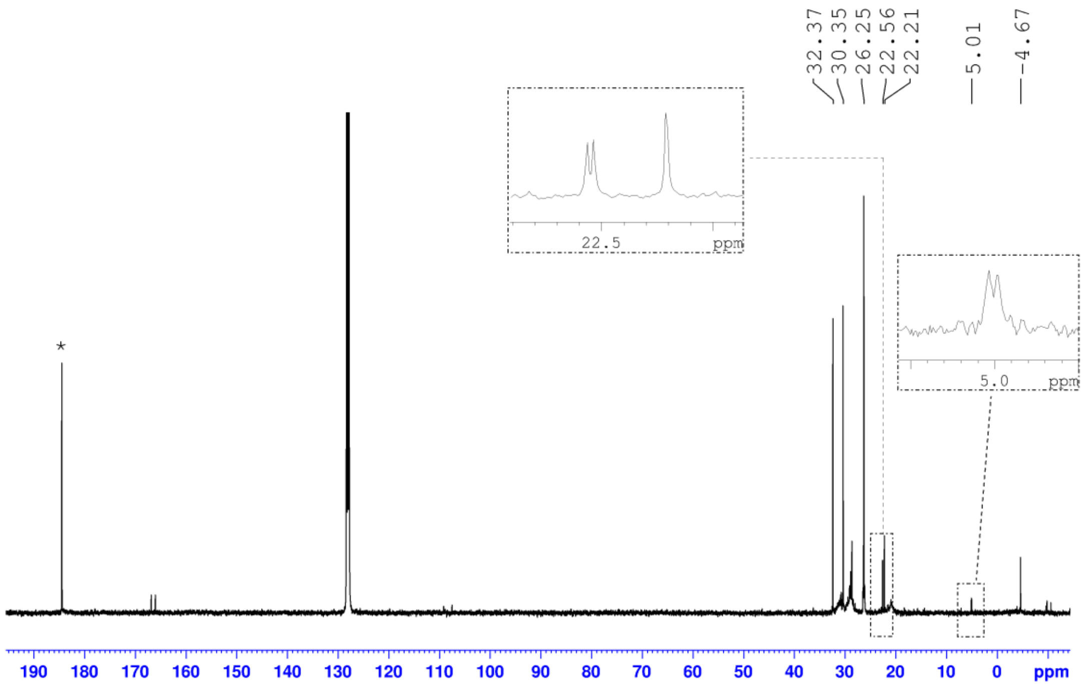

Figure S28. $^{13}C\{^{1}H\}$ NMR spectrum of **4'** (from ^{13}CO) in C_6D_6 at ambient temperature (* = free ^{13}CO).

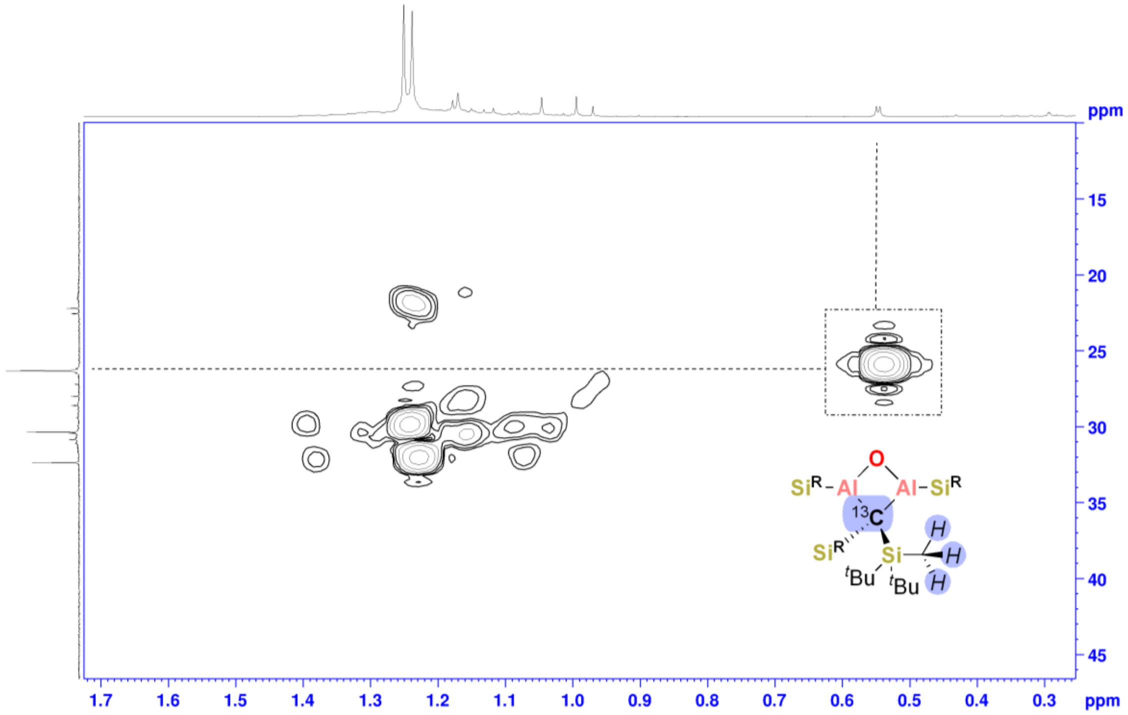

Figure S29. HMBC (^{1}H-^{13}C) NMR spectrum of **4'** (from ^{13}CO), within the range 1.7 – 0.25/10 – 46 ppm highlighting the endocyclic carbon coupling to the silyl ligand methyl group. (^{1}H NMR spectrum = top/ ^{13}C NMR spectrum = left)

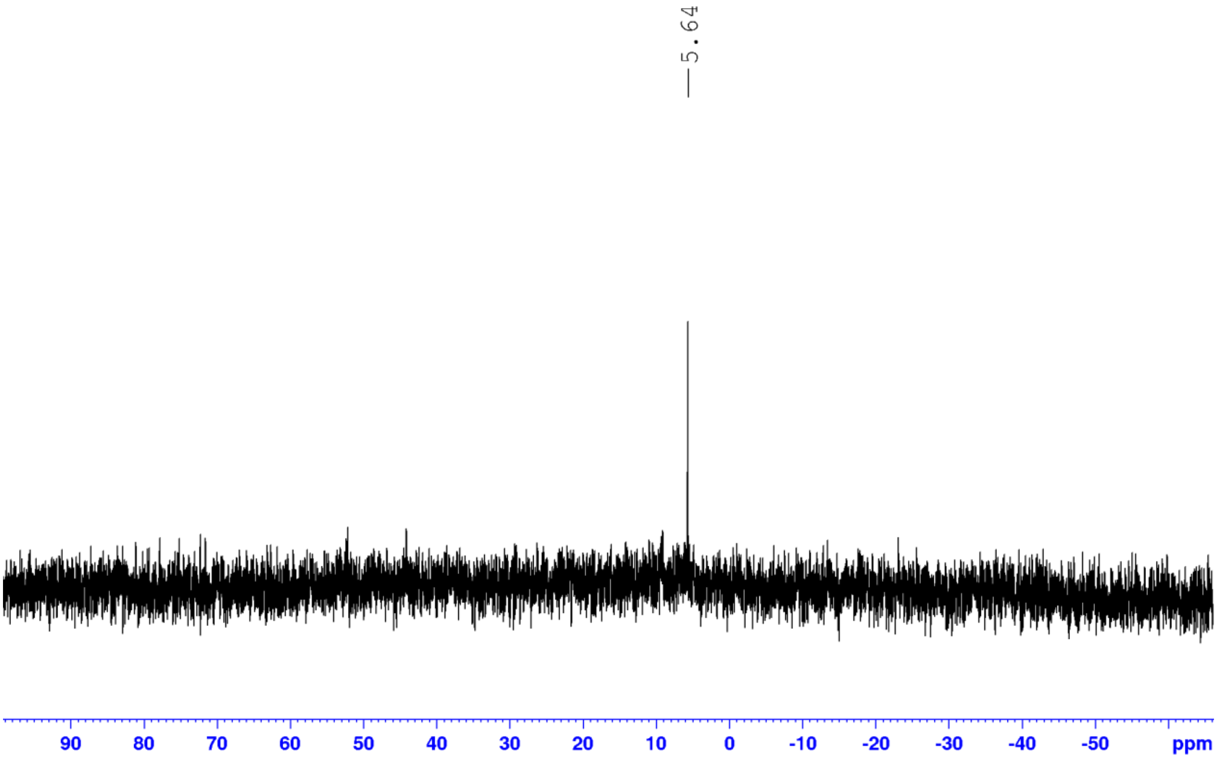

Figure S30. $^{29}Si\{^{1}H\}$ NMR spectrum of **4'** in C_6D_6 at ambient temperature.

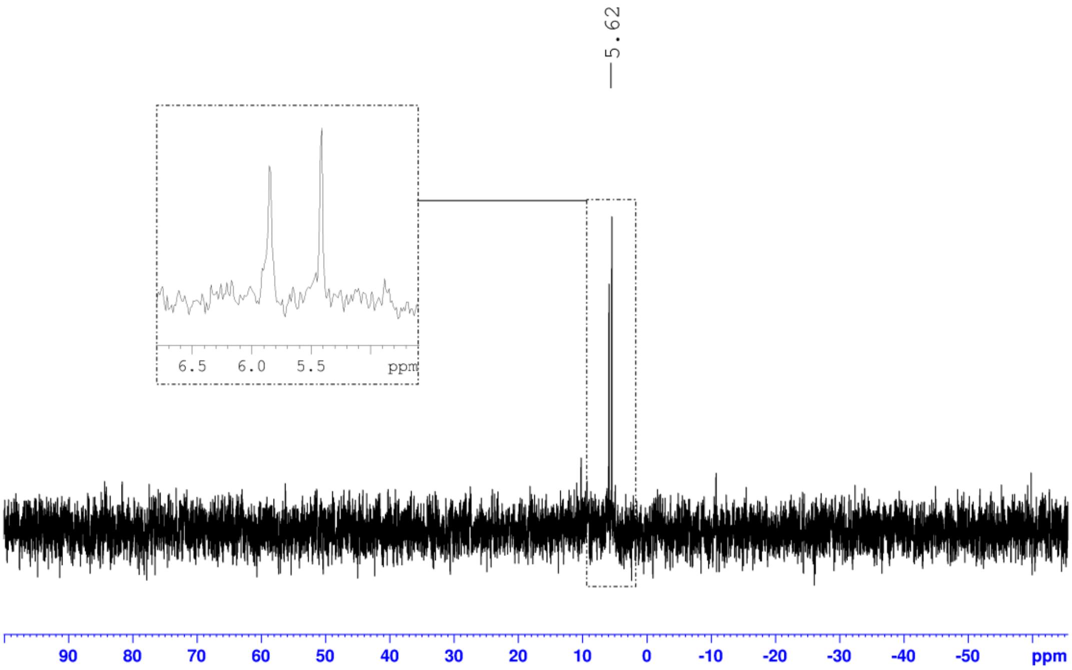

Figure S31. $^{29}Si\{^{1}H\}$ NMR spectrum of **4'** (from ^{13}CO) in C_6D_6 at ambient temperature.

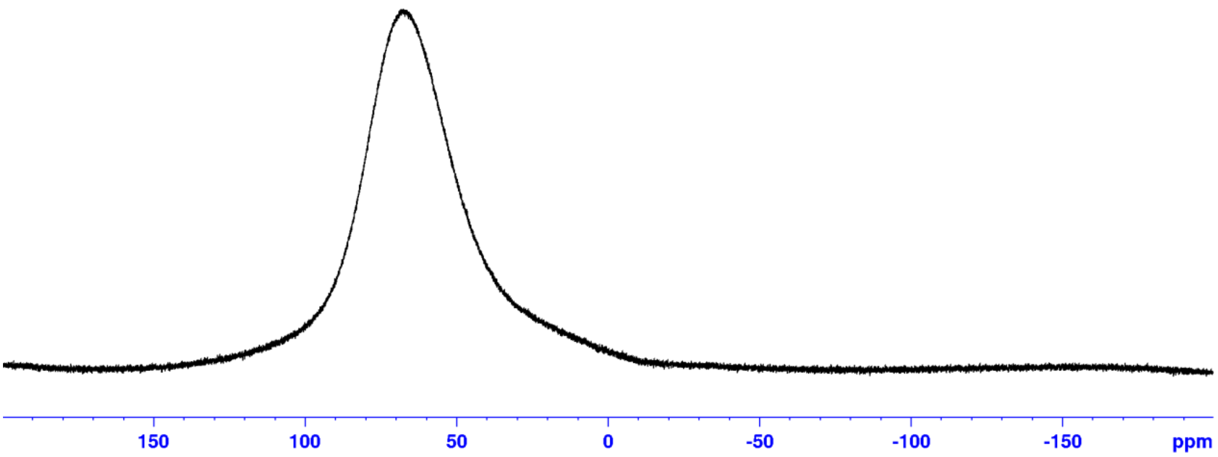

Figure S32. ^{27}Al NMR spectrum of **4’** in C_6D_6 at ambient temperature.

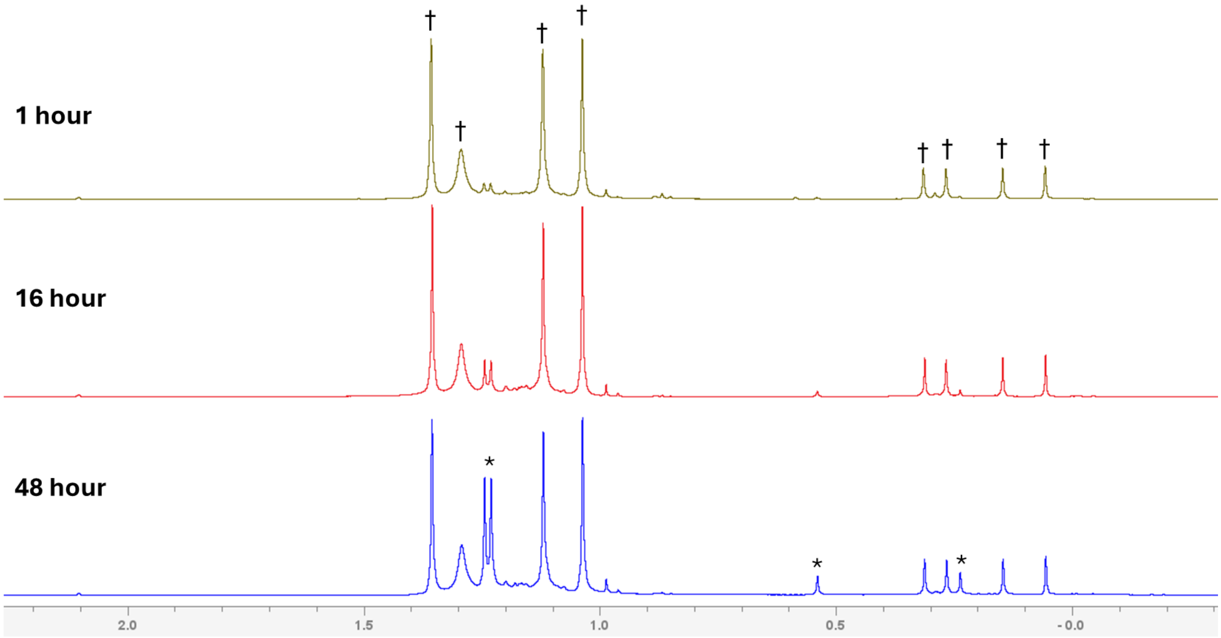

Figure S33. ^{1}H NMR spectrum of **4** in C_6D_6 at ambient temperature after 1/16/48 hours, showing over time the formation of 4’ († = **4**, * = **4’**)

4. UV-Vis Spectroscopy

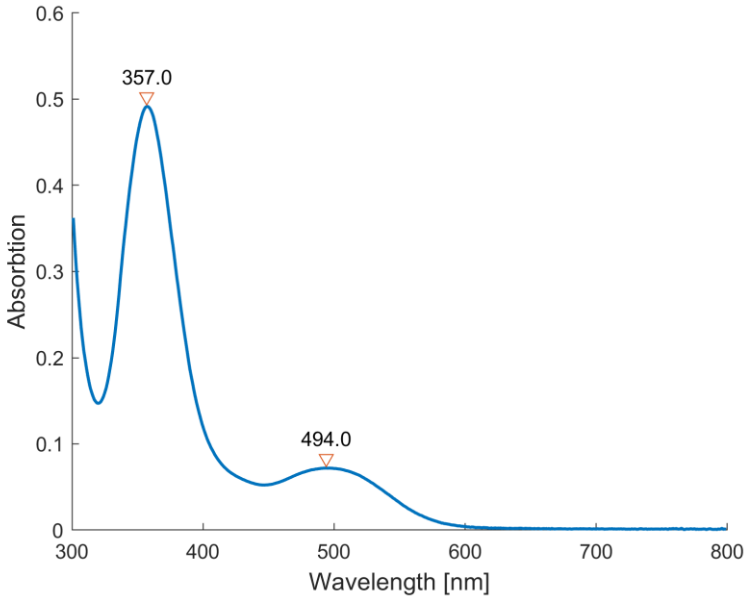

Figure S34. UV-vis spectrum of **3** in toluene.

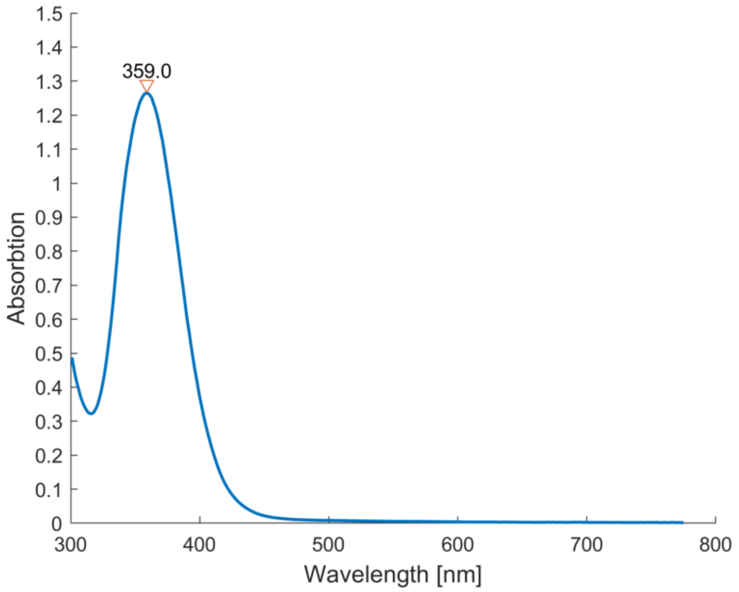

Figure S35. UV-vis spectrum of **4** in toluene.

5. Mass Spectrometry

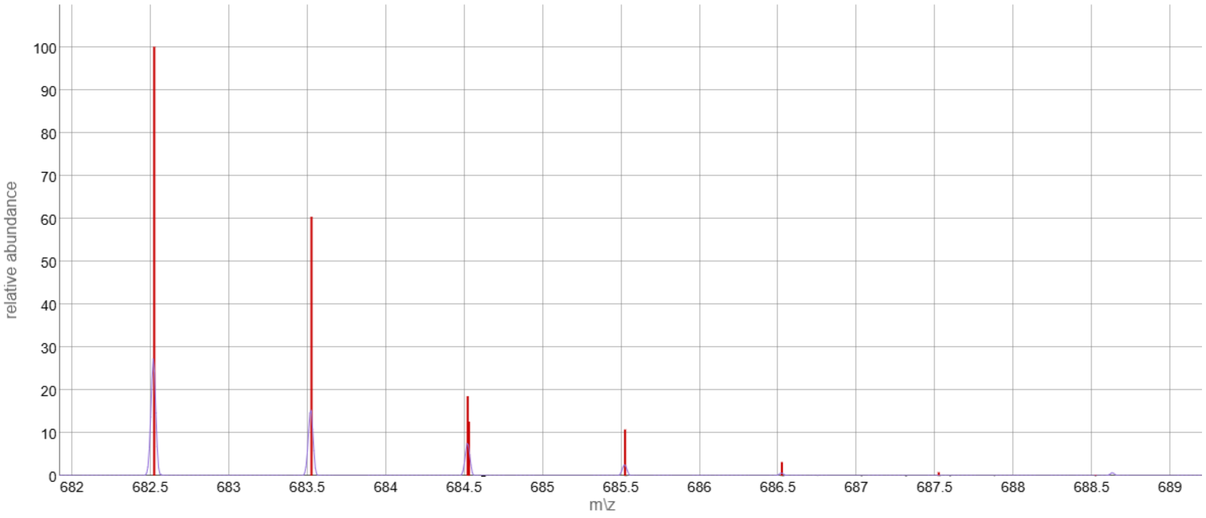

Figure S36. LIFDI-MS spectrum (isotope patter) of **3** (measured: violet, simulated: red).

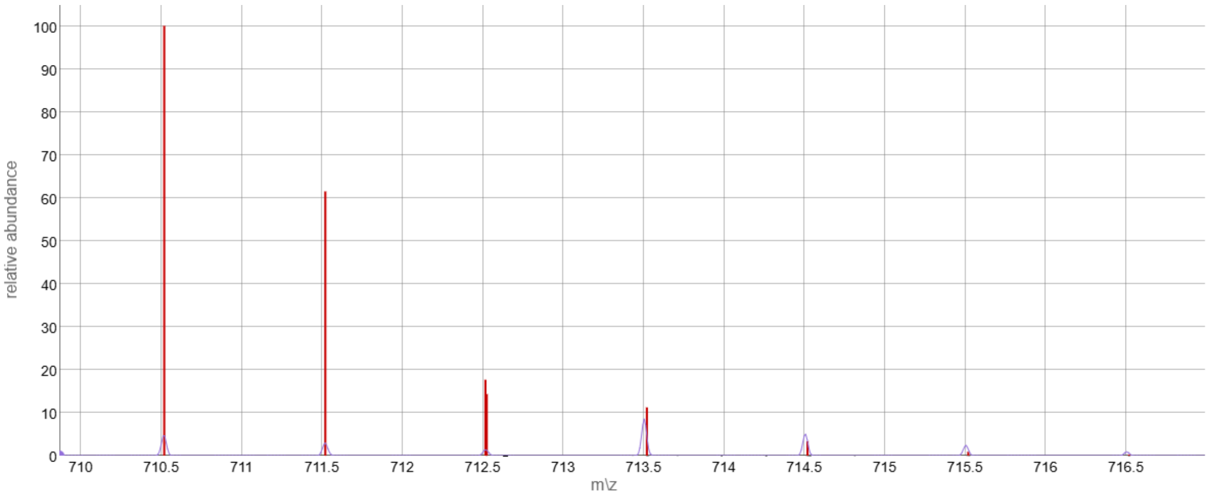

Figure S37. LIFDI-MS spectrum (isotope pattern) of **4** (measured: violet, simulated: red).

6. IR Spectroscopy

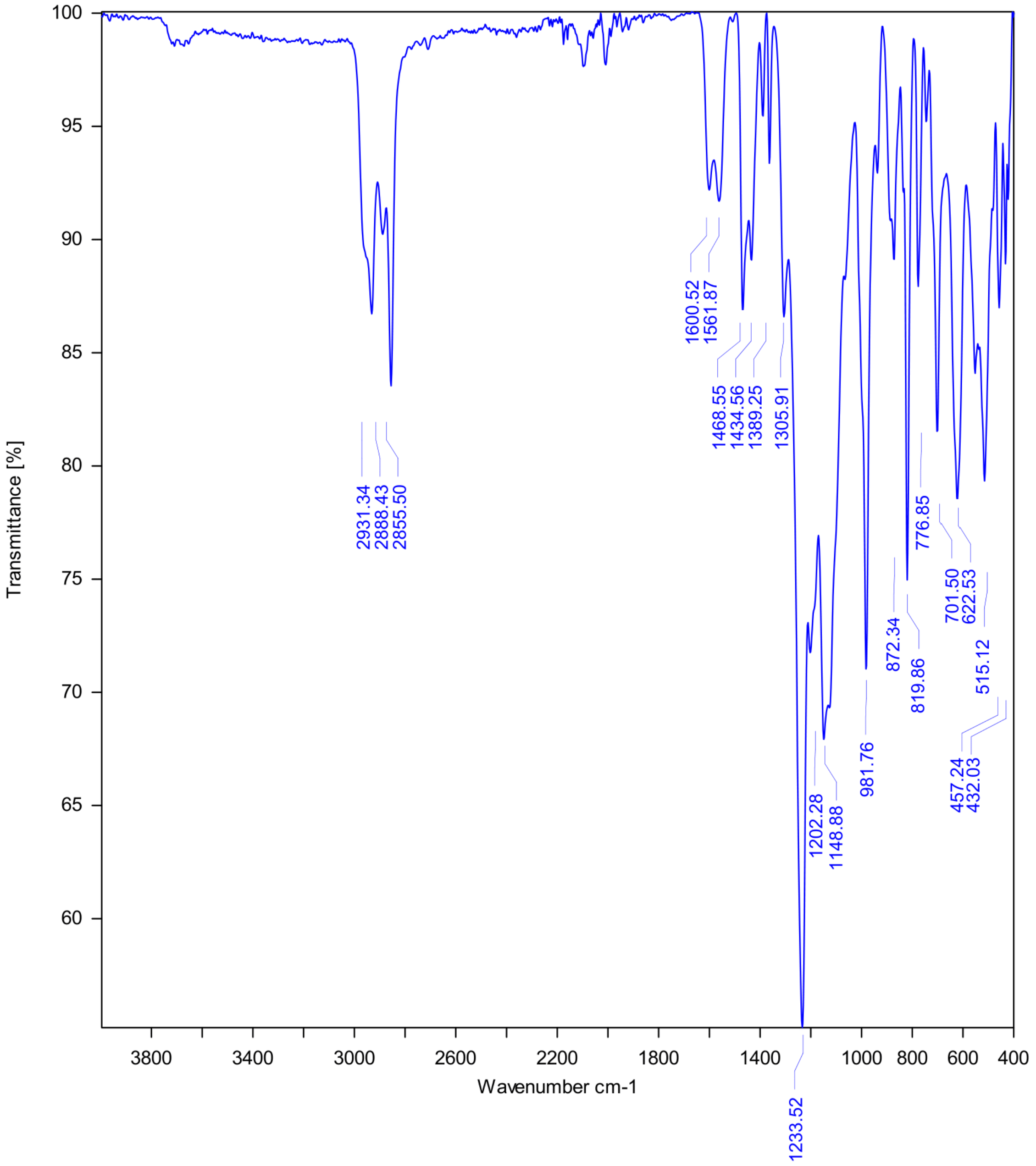

Figure S38. Experimental IR spectrum of compound **5**.

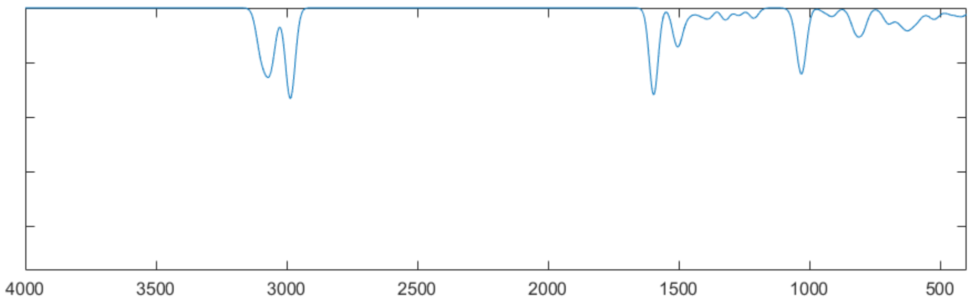

Figure S39. r^2SCAN-3c//r^2SCAN-3c calculated IR spectrum of **5'**, plotted by orca_mapspc (-w40 -x0400 -x4000).

Analytical frequencies of the monomer of **5** (**5'**) were calculated and the IR spectrum was plotted (Figure S39) showing a characteristic vibration at 1596.7 cm^{-1}, which corresponds to the C-C and C-O displacement. The displacement vectors of vibrational frequency at 1596.7 cm^{-1} are shown in Figure S40. The atoms that are most strongly involved in this vibration are the three carbon atoms of the four membered ring, as well as the two endocyclic oxygen atoms. This vibration corresponds to the experimentally observed peaks in the 1600 region.

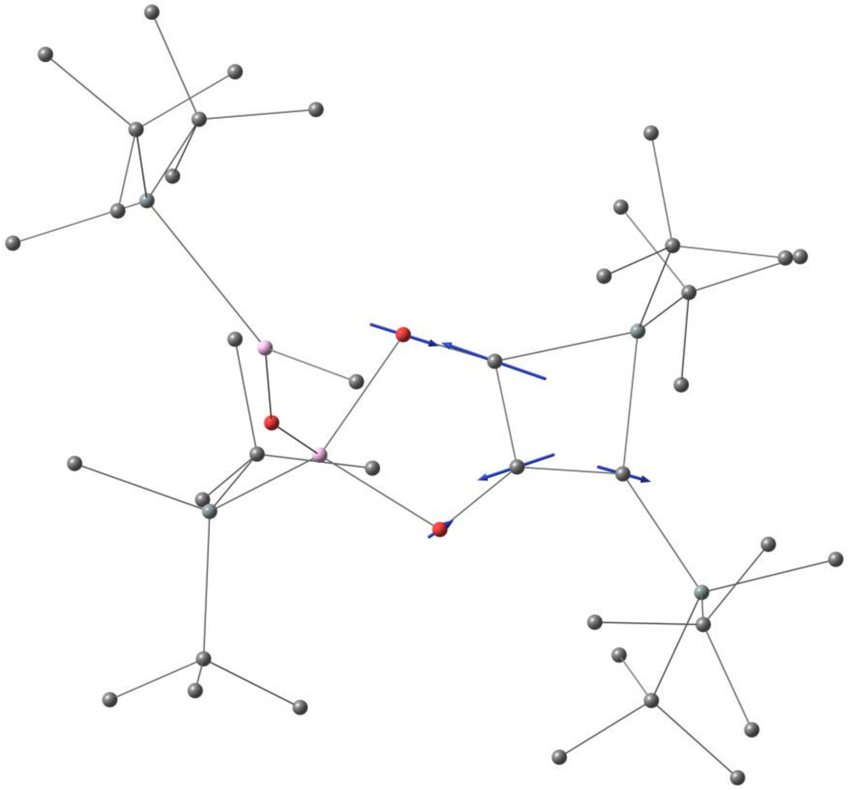

Figure S40. Optimized geometry of **5** monomer (**5'**) (hydrogen are omitted for clarity). The displacement vectors for the vibrational frequency at 1596.7 cm^{-1} are shown as blue arrows.

7. Quantum Chemical Calculations

Calculations were carried out using ORCA 5.0.4 software.[8] Geometry optimizations were carried using the r^2SCAN-3c composite method, utilizing the regularized and restored SCAN functional,[9,10] geometrical counterpoise correction gCP,[11] the atom-pairwise dispersion correction based on tight binding partial charges (D4),[12,13,14] the def2-mTZVPP basis set and def2-mTZVPP/J auxiliary basis set.[15] The optimized geometries were verified as minima or transition states by analytical frequency calculations. The transition states were additionally verified by IRC calculations. Single point calculations of the optimized geometries were carried out at the r^2SCAN-3c level using the SMD solvation module to obtain electrostatic contribution and the cavity term, in order to account for the solvent effects.[16] To get more accurate electronic energies for the mechanistic investigations, single point calculations of the r^2SCAN-3c optimized geometries were carried using the PW6B95 functional,[17] with D4 dispersion correction, the def2-QZVPP[18] basis set and def2/J[19] and def2-QZVPP/C[20] auxiliary basis sets. The method is denoted as (SMD=Benzene)PW6B95-D4/def2-QZVPP //r^2SCAN-3c. The summary of the thermochemistry results is presented in Table S1. The NBO analysis was done using the NBO7 software,[21] at the PBE0[22]/def2-TZVP[18]/r^2SCAN-3c level of theory. ^{13}C NMR chemical shift of was calculated at the PBE0/6-311+G(2d,p)[23]/ (CPCM=Benzene) level using the r^2SCAN-3c optimized geometries. The intrinsic atomic orbitals and intrinsic bond orbitals (IAOIBO) calculation,[24] was carried out at the PBE0/def2-TZVP/r^2SCAN-3c level of theory. In order to demonstrate the suitability of r^2SCAN-3c on the molecular systems described in the article we compared the X-ray structures of compounds **3** and **4** with the r^2SCAN-3c optimized geometries. Taking into consideration all the bond and angles (excluding hydrogen atoms), the following mean square errors (RMSEs) were obtained: In **3** the RMSE for bonds is 0.011 Å and for angles it is 0.95°; in **4** the RMSE for bonds is 0.019 Å and for angles it is 0.14°. These results suggest that r^2SCAN-3c is a suitable method for obtaining geometries of species featured in this article.

Table S1. Calculated energies (Eh). E_{PW6B95} - electronic energy at the PW6B95-D4/def2-QZVPP//r^2SCAN-3c level; G-E_{el} - Gibbs energy minus the electronic energy at the r^2SCAN-3c// r^2SCAN-3c level; G_{cds} (cavity term) and G_{enp} (electrostatic contribution) at r^2SCAN-3c (SMD=Benzene)// r^2SCAN-3c level; G_{conc} - concentration-induced free-energy shift (G_{conc} = RTln(24.5)); G – free energy at the (SMD=Benzene)PW6B95-D4/def2-QZVPP//r^2SCAN-3c level, G = E_{PW6B95} + [G-E_{el}] + G_{cds} + G_{enp} + G_{conc}. Thermochemistry at 298.15 K.

Compound	ID	E_{PW6B95}	G-E_{el}	G_{cds}	G_{enp}	G_{conc}	G
**3**	3726660	-3069.56048	1.03832	-0.01322	-0.01405	0.00302	-3068.54641
**4**	3726661	-3183.21334	1.04713	-0.01396	-0.01422	0.00302	-3182.19137
**TS1**	3759199	-3183.05127	1.04330	-0.01326	-0.01466	0.00302	-3182.03288
**INT1**	3759410	-3183.07454	1.04723	-0.01205	-0.01458	0.00302	-3182.05091
**TS2**	3743193	-3183.05827	1.04810	-0.01060	-0.01397	0.00302	-3182.03173

**INT2**	3747364	-3183.06137	1.04722	-0.01066	-0.01409	0.00302	-3182.03588
**TS3**	3759220	-3183.05617	1.04864	-0.01241	-0.01402	0.00302	-3182.03094
**INT3**	3760203	-3183.08377	1.04915	-0.01291	-0.01428	0.00302	-3182.05879
**TS4**	3769930	-3183.07184	1.05044	-0.01242	-0.01408	0.00302	-3182.04488
**INT4**	3771479	-3183.07829	1.04929	-0.01261	-0.01404	0.00302	-3182.05263
**TS5**	3776176	-3183.06415	1.04670	-0.01585	-0.01460	0.00302	-3182.04488
**INT5**	3762379	-3183.10395	1.04671	-0.01334	-0.01461	0.00302	-3182.08218
**TS6**	3760793	-3183.09615	1.04785	-0.01254	-0.01464	0.00302	-3182.07246
**INT6 (4')**	3760204	-3183.21952	1.05469	-0.01158	-0.01461	0.00302	-3182.18800
**TS7**	3759407	-3183.17965	1.05174	-0.01126	-0.01467	0.00302	-3182.15082
**INT7**	3738342	-3183.18251	1.04733	-0.01198	-0.01514	0.00302	-3182.15929
**TS8**	3737383	-3183.18106	1.04714	-0.01302	-0.01423	0.00302	-3182.15815
**TS9**	3864112	-3296.71099	1.05337	-0.01402	-0.01389	0.00302	-3295.68250
**INT9**	3868440	-3296.71287	1.05449	-0.01329	-0.01447	0.00302	-3295.68313
**TS10**	3863108	-3296.69877	1.05673	-0.01403	-0.01434	0.00302	-3295.66739
**INT10**	3877276	-3296.70617	1.05804	-0.01592	-0.01432	0.00302	-3295.67535
**TS11**	3873752	-3296.70439	1.05866	-0.01638	-0.01446	0.00302	-3295.67355
**INT11**	627118	-3296.72013	1.05706	-0.01988	-0.01465	0.00302	-3295.69459
**TS12**	3869124	-3296.70944	1.05568	-0.01790	-0.01498	0.00302	-3295.68361
**INT12**	3872548	-3296.75618	1.05042	-0.01275	-0.01566	0.00302	-3295.73115
**INT13**	3898761	-3410.26646	1.05447	-0.01182	-0.01625	0.00302	-3409.23703
**TS13**	3897069	-3410.24829	1.05590	-0.01294	-0.01616	0.00302	-3409.21848
**INT14**	3890806	-3410.25312	1.05720	-0.01379	-0.01590	0.00302	-3409.22260
**TS14**	3882217	-3410.24161	1.05856	-0.01295	-0.01596	0.00302	-3409.20894
**5'**	3842322	-3410.32720	1.05832	-0.01342	-0.01596	0.00302	-3409.29524
**4''**	1641308	-3183.18624	1.04249	-0.01641	-0.01448	0.00302	-3182.17162
CO	612445	-113.50052	-0.01413	-0.00046	0.00476	0.00302	-113.50733

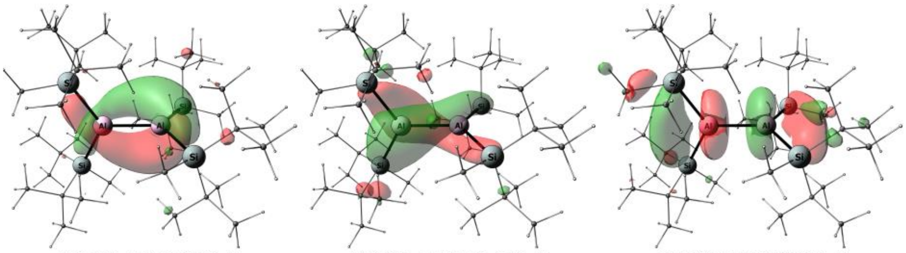

MO 192. LUMO -2.03 eV		
Bonding	N.B.	A.B.
5.6%	75.3%	19.1%

-0.733*[192] LV (1) Al2	53.7%
0.431*[193] LV (1) Al3	18.6%

MO 193. LUMO+1 -1.74 eV		
Bonding	N.B.	A.B.
6.1%	72.4%	21.5%

-0.720*[193] LV (1) Al3	51.8%
-0.420*[192] LV (1) Al2	17.6%

MO 194. LUMO+2 0.22 eV		
Bonding	N.B.	A.B.
2.1%	14.4%	83.5%

-0.576*[198] BD*(1) Al2-Al3	33.2%
0.269*[207] BD*(1) Si5-C51	7.2%
-0.260*[209] BD*(1) Si6-C93	6.8%

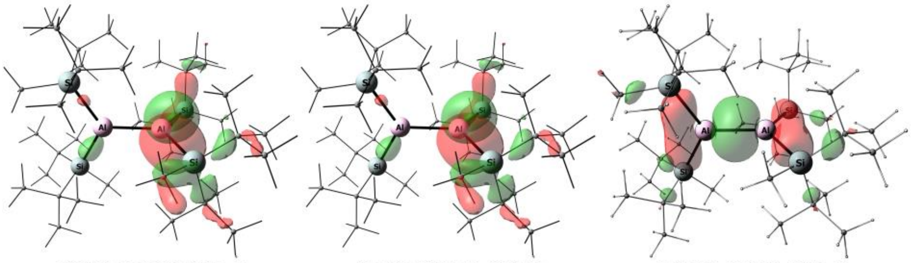

MO 189. HOMO-2 -6.05 eV		
Bonding	N.B.	A.B.
95.1%	2.5%	2.5%

-0.554*[72] BD(1) Al2-Si5	30.7%
0.541*[67] BD(1) Si1-Al2	29.3%

MO 190. HOMO-1 -6.02 eV		
Bonding	N.B.	A.B.
94.9%	2.5%	2.6%

0.553*[74] BD(1) Al3-Si6	30.6%
-0.541*[73] BD(1) Al3-Si4	29.3%

MO 191. HOMO -5.45 eV		
Bonding	N.B.	A.B.
95.5%	1.3%	3.2%

-0.664*[71] BD(1) Al2-Al3	44.1%
0.329*[67] BD(1) Si1-Al2	10.8%
0.309*[73] BD(1) Al3-Si4	9.5%
0.297*[74] BD(1) Al3-Si6	8.8%
0.283*[72] BD(1) Al2-Si5	8.0%

Figure S41. NBO analysis of selected canonical molecular orbitals of **3**.

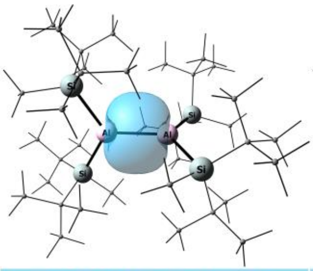

NBO 71. BD(1) Al2-Al3 occ. 1.92	
49.45% Al2 sp1.91	
50.55% Al3 sp1.91	
Acceptor	E(2)(kcal mol^{-1})
199. BD*(1) Al2-Si5	3.3
201. BD*(1) Al3-Si6	3.1
194. BD*(1) Si1-Al2	3.0
200. BD*(1) Al3-Si4	2.9
209. BD*(1) Si6-C93	2.0

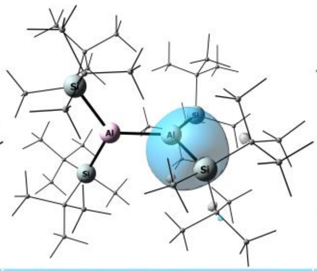

NBO 67. BD(1) Si1-Al2 occ. 1.91	
64.08% Si1 sp2.42	
35.92% Al2 sp2.08	
Acceptor	E(2)(kcal mol^{-1})
199. BD*(1) Al2-Si5	4.2
309. BD*(1) C114-C123	3.0
1694. RY (1) C114	2.5
215. BD*(1) C11-C17	2.4
305. BD*(1) C110-H112	2.2

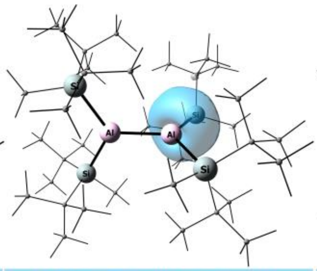

NBO 72. BD(1) Al2-Si5 occ. 1.91	
35.73% Al2 sp2.01	
64.27% Si5 sp2.42	
Acceptor	E(2)(kcal mol^{-1})
194. BD*(1) Si1-Al2	4.5
243. BD*(1) C38-C47	3.1
198. BD*(1) Al2-Al3	2.6
229. BD*(1) C25-C26	2.4
852. RY (1) C38	2.4

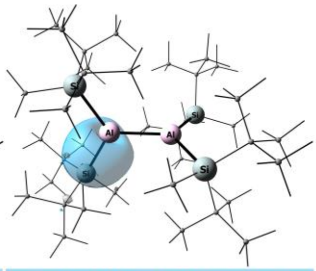

NBO 73. BD(1) Al3-Si4 occ. 1.91	
36.37% Al3 sp2.05	
63.63% Si4 sp2.40	
Acceptor	E(2)(kcal mol^{-1})
201. BD*(1) Al3-Si6	3.8
192. LV (1) Al2	2.7
266. BD*(1) C67-C72	2.5
218. BD*(1) C12-C59	2.5
257. BD*(1) C55-H57	2.3

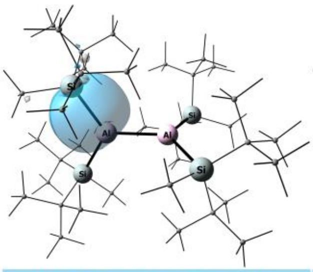

NBO 74. BD(1) Al3-Si6 occ. 1.91	
36.06% Al3 sp2.03	
63.94% Si6 sp2.39	
Acceptor	E(2)(kcal mol^{-1})
200. BD*(1) Al3-Si4	4.0
294. BD*(1) C97-C106	2.9
279. BD*(1) C80-C89	2.6
1504. RY (1) C97	2.4
192. LV (1) Al2	2.3

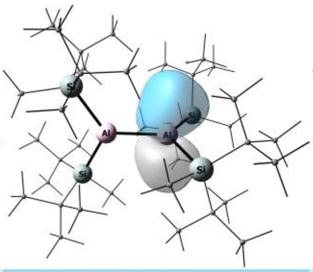

NBO 192. LV(1) Al2 occ. 0.10	
sp99.99	
Donor	E(2)(kcal mol^{-1})
73. BD(1) Al3-Si4	2.7
108. BD(1) C30-H31	2.6
74. BD(1) Al3-Si6	2.3
122. BD(1) C43-H46	2.1
99. BD(1) C21-H22	2.0

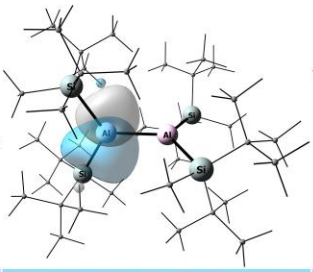

NBO 193. LV(1) Al3 occ. 0.11	
sp99.99	
Donor	E(2)(kcal mol^{-1})
94. BD(1) C13-H15	5.7
153. BD(1) C81-H82	5.1
72. BD(1) Al2-Si5	2.3
117. BD(1) C39-H40	1.9
95. BD(1) C13-H16	1.1

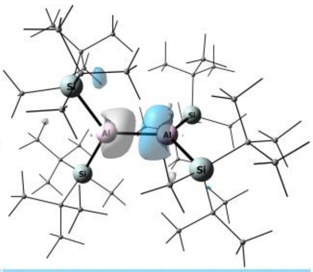

NBO 198. BD*(1) Al2-Al3 occ. 0.04	
50.55% Al2 sp1.91	
49.45% Al3 sp1.91	
Donor	E(2) (kcal mol-1)
72. BD(1) Al2-Si5	2.6
74. BD(1) Al3-Si6	2.3
67. BD(1) Si1-Al2	2.1
73. BD(1) Al3-Si4	2.1

Figure S42. Selected NBOs of **3** , their atomic orbital compositions and five largest donor-acceptor interactions, according to the second-order perturbation theory.

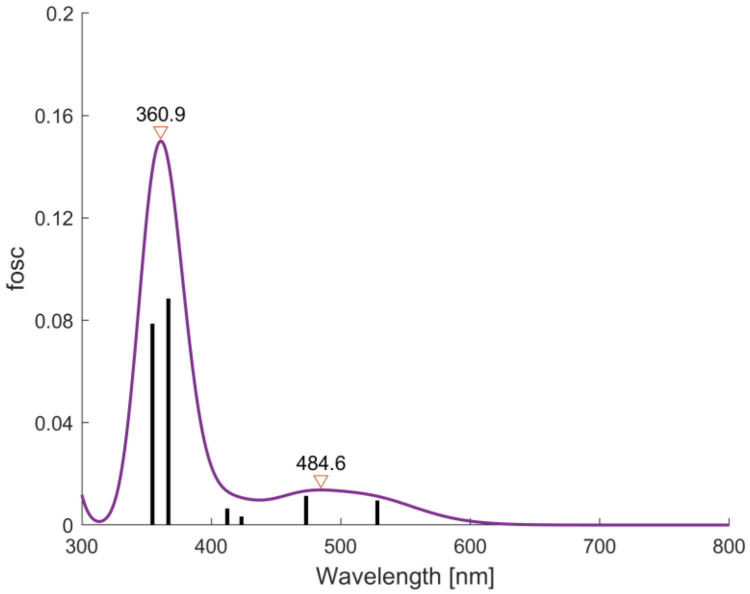

Figure S43. Simulated absorption spectrum of **3** in 300-800 nm region, based on the TDDFT calculation at the PBE0/def2-TZVP(CPCM=Toluene)//r^{2}SCAN-3c level of theory. The corresponding excitations are shown as black vertical line.

Table S2. TD-DFT calculated excites states (S1-S6) and the corresponding Natural Transition Orbitals (NTOs).

State	Transitions	Wavelength (nm)	fosc	NTOs
1	191a* → 192a* : 0.991343	528.2	0.009592587	191a* → 192a* : n= 0.99843327<br>190a* → 193a* : n= 0.00282056<br>189a* → 193a : n= 0.00190287
2	191a* → 193a* : 0.989364	473.2	0.011406543	191a* → 192a* : n= 0.99643701<br>190a* → 193a* : n= 0.00402930<br>189a* → 193a : n= 0.00179308
3	189a* → 192a* : 0.820721<br>189a* → 193a* : 0.073069<br>190a* → 192a* : 0.061288<br>190a* → 193a* : 0.039188	423.3	0.003308772	191a* → 192a* : n= 0.98713702<br>190a* → 193a* : n= 0.01326142
4	189a* → 192a* : 0.024869<br>189a* → 193a* : 0.042680<br>190a* → 192a* : 0.626501<br>190a* → 193a* : 0.300374	412.3	0.006469624	191a* → 192a* : n= 0.99360402<br>190a* → 193a* : n= 0.00631438<br>189a* → 193a : n= 0.00145522
5	189a* → 192a* : 0.056347<br>189a* → 193a* : 0.061227<br>190a* → 192a* : 0.291631<br>190a* → 193a* : 0.575556	366.8	0.08849334	191a* → 192a* : n= 0.98520730<br>190a* → 193a* : n= 0.01284178<br>189a* → 193a : n= 0.00282285
6	189a* → 192a* : 0.087290<br>189a* → 193a* : 0.811917<br>190a* → 192a* : 0.012241<br>190a* → 193a* : 0.072285	354.5	0.078702413	191a* → 192a* : n= 0.98575549<br>190a* → 193a* : n= 0.01399585<br>189a* → 193a : n= 0.00101174

The TDDFT calculations (Figure S43, Table S2) reproduce well the experimental UV-Vis spectrum of **3** (Figure S43), showing two peaks in the visible (experimental 494.0 nm, simulated 484.6 nm) and the UV region (experimental 357.0 nm, simulated 360.9 nm). According to the calculations there are 6 excited states in the 300-800 nm region. The peak in the visible region, from which the red colour of **3** stems, originates predominantly from excited states 1,2,3 and 4 with the corresponding excitations at 528.2, 473.2, 423.3 and 412.3. The peak in the UV region originated for the excitations at 366.8 and 354.5 nm. All of the excitation in the 300-800 region correspond

to the transitions from σ(Al-Al) and σ(Al-Si) to p orbitals of the Al centres. The corresponding Natural Transition Orbitals are presented in Figure S44.

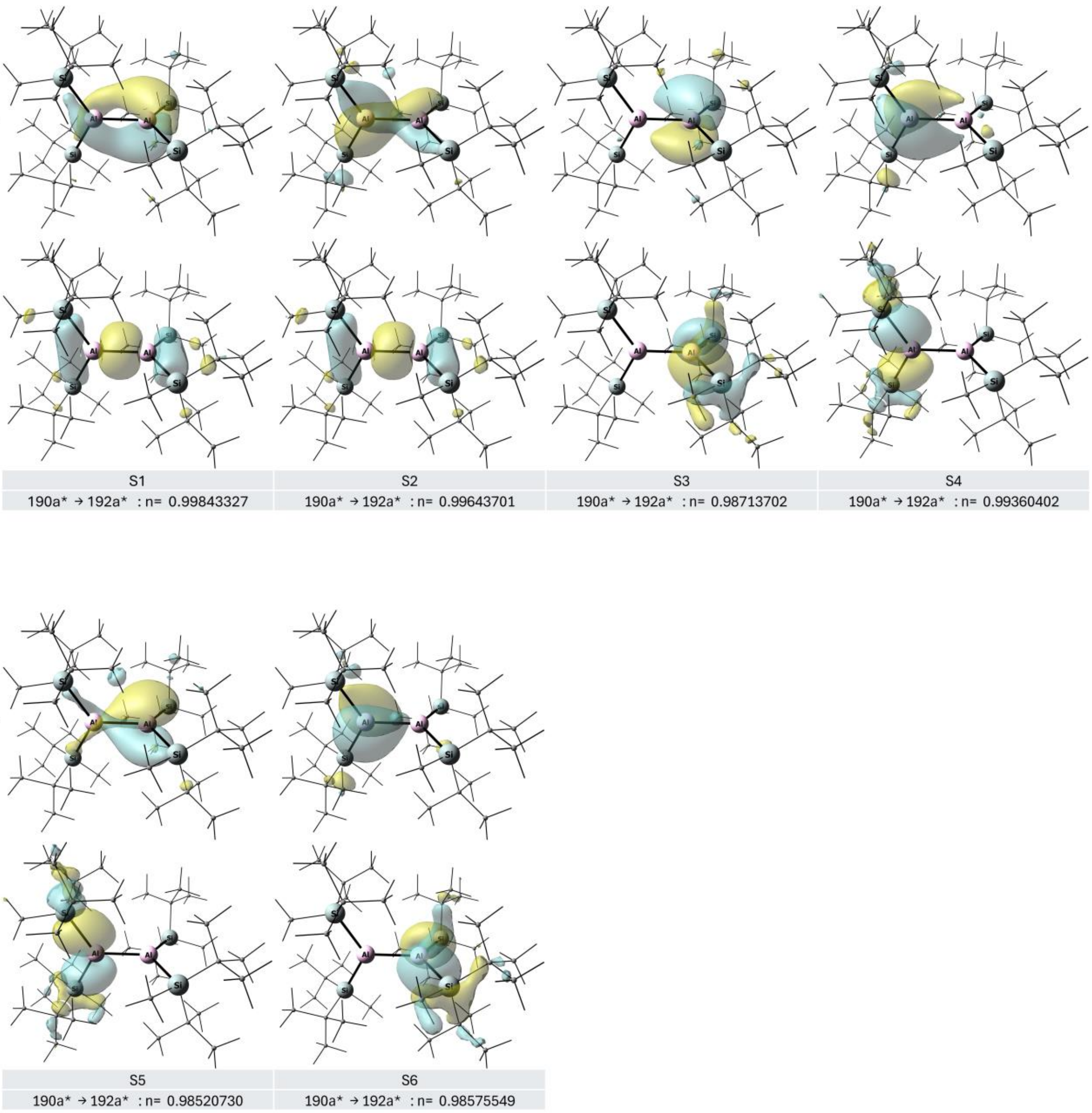

Figure S44. Natural transition orbital of **3** for the excited states S_1-S_6. For each state the donor orbital is shown in the bottom and the acceptor orbital on the top.

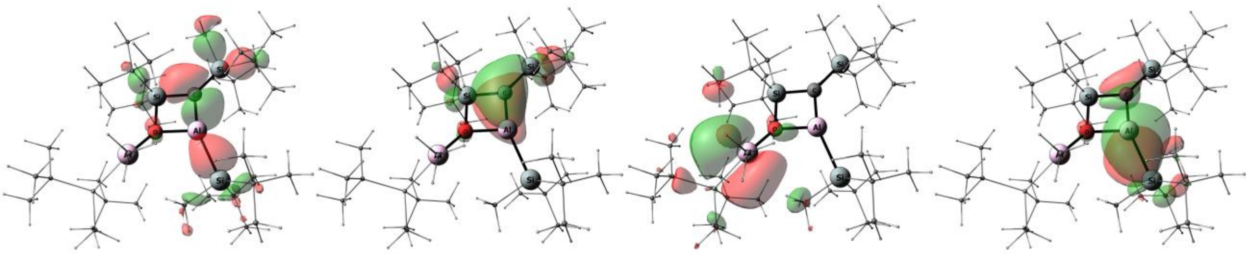

MO 197. HOMO-1 -6.24 eV

Bonding	N.B.	A.B.
93.5%	1.7%	4.9%

-0.554*[84]	BD(1) Al4-C8	30.7%
0.444*[72]	BD(1) Si1-Al4	19.7%
0.325*[77]	BD(1) Si2-C8	10.6%
-0.273*[83]	BD(1) Si3-C44	7.5%
0.242*[81]	BD(1) Si3-C26	5.9%

MO 198. HOMO -5.07 eV

Bonding	N.B.	A.B.
89.5%	2.7%	7.8%

0.866*[85]	BD(2) Al4-C8	75.0%

MO 199. LUMO -1.07 eV

Bonding	N.B.	A.B.
4.8%	64.8%	30.4%

0.545*[200]	LV (1) Al5	29.7%
-0.487*[201]	LV (2) Al5	23.7%
-0.293*[219]	BD*(1) Si6-C72	8.6%

MO 200. LUMO+1 -0.72 eV

Bonding	N.B.	A.B.
3.2%	10.5%	86.3%

0.782*[215]	BD*(2) Al4-C8	61.2%
0.301*[203]	BD*(1) Si1-C9	9.1%
-0.269*[204]	BD*(1) Si1-C11	7.2%

Figure S45. NBO analysis of selected canonical molecular orbitals of **4**.

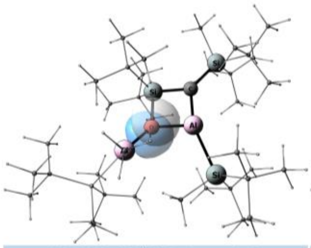

NBO 69. LP(1) O7 occ. 1.94

sp99.99

Acceptor	E(2)(kcal mol^{-1})
209. BD*(1) Si2-C17	3.6
215. BD*(2) Al4-C8	3.4
208. BD*(1) Si2-C10	2.1
443. RY (3) Al5	2.0
216. BD*(1) Al5-Si6	1.4

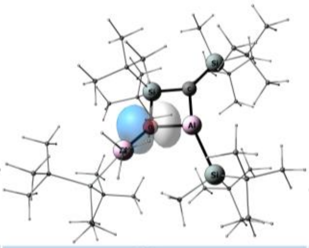

NBO 70. LP(2) O7 occ. 1.93

sp99.99

Acceptor	E(2)(kcal mol^{-1})
201. LV (2) Al5	8.4
217. BD*(1) Al5-C18	4.5
442. RY (2) Al5	4.3
216. BD*(1) Al5-Si6	3.8
415. RY (3) Al4	2.5

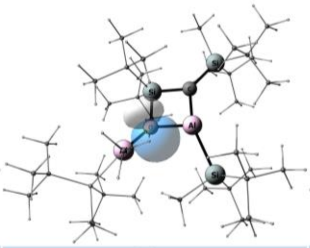

NBO 71. LP(3) O7 occ. 1.89

sp5.41

Acceptor	E(2)(kcal mol^{-1})
199. LV (1) Al4	19.2
200. LV (1) Al5	17.0
206. BD*(1) Si2-O7	9.3
217. BD*(1) Al5-C18	4.6
359. RY (3) Si2	4.3

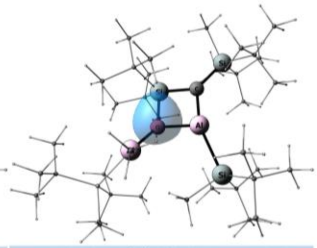

NBO 76. BD(1) Si2-O7 occ. 1.92

8.33% Si2 sp9.45

91.67% O7 sp0.21

Acceptor	E(2)(kcal mol^{-1})
199. LV (1) Al4	28.9
201. LV (2) Al5	17.5
200. LV (1) Al5	12.6
217. BD*(1) Al5-C18	2.9
214. BD*(1) Al4-C8	2.1

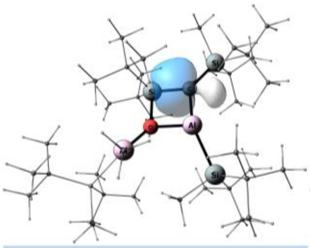

NBO 77. BD(1) Si2-C8 occ. 1.93

22.72% Si2 sp2.02

77.28% C8 sp1.78

Acceptor	E(2)(kcal mol^{-1})
210. BD*(1) Si3-C8	4.9
214. BD*(1) Al4-C8	4.8
199. LV (1) Al4	2.9
209. BD*(1) Si2-C17	2.5
208. BD*(1) Si2-C10	2.3

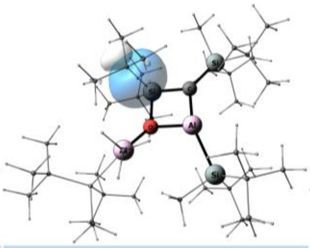

NBO 78. BD(1) Si2-C10 occ. 1.91

22.93% Si2 sp2.44

77.07% C10 sp3.00

Acceptor	E(2)(kcal mol^{-1})
209. BD*(1) Si2-C17	5.1
261. BD*(1) C40-H42	3.9
294. BD*(1) C81-H83	3.8
258. BD*(1) C36-H38	3.7
207. BD*(1) Si2-C8	3.0

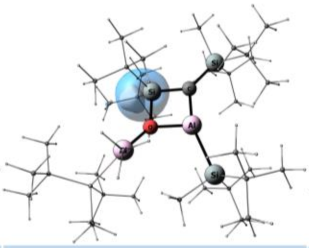

NBO 79. BD(1) Si2-C17 occ. 1.91

23.12% Si2 sp2.42

76.88% C17 sp3.04

Acceptor	E(2)(kcal mol^{-1})
208. BD*(1) Si2-C10	5.1
291. BD*(1) C77-H79	3.9
284. BD*(1) C68-H70	3.9
267. BD*(1) C48-H50	3.8
207. BD*(1) Si2-C8	2.9

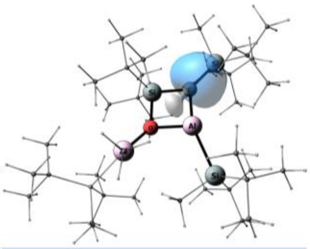

NBO 80. BD(1) Si3-C8 occ. 1.94

23.28% Si3 sp2.48

76.72% C8 sp1.42

Acceptor	E(2)(kcal mol^{-1})
214. BD*(1) Al4-C8	4.8
207. BD*(1) Si2-C8	3.5
212. BD*(1) Si3-C31	1.7
211. BD*(1) Si3-C26	1.7
213. BD*(1) Si3-C44	1.2

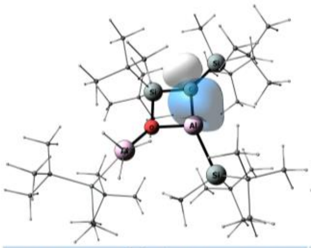

NBO 84. BD(1) Al4-C8 occ. 1.87

11.79% Al4 sp1.06

88.21% C8 sp3.44

Acceptor	E(2)(kcal mol^{-1})
202. BD*(1) Si1-Al4	9.1
206. BD*(1) Si2-O7	8.4
386. RY (2) Si3	5.3
358. RY (2) Si2	4.8
213. BD*(1) Si3-C44	3.7

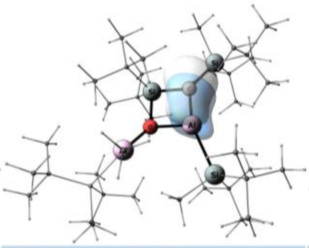

NBO 85. BD(2) Al4-C8 occ. 1.81

11.63% Al4 sp99.99

88.37% C8 sp99.99

Acceptor	E(2)(kcal mol^{-1})
208. BD*(1) Si2-C10	13.5
209. BD*(1) Si2-C17	13.4
212. BD*(1) Si3-C31	7.3
385. RY (1) Si3	5.0
211. BD*(1) Si3-C26	4.6

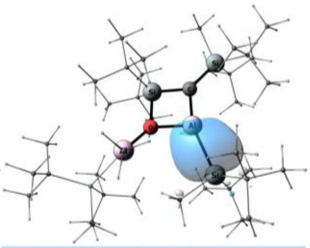

NBO 72. BD(1) Si1-Al4 occ. 1.89

66.78% Si1 sp2.64

33.22% Al4 sp1.13

Acceptor	E(2)(kcal mol^{-1})
214. BD*(1) Al4-C8	22.6
200. LV (1) Al5	3.2
221. BD*(1) C9-C32	3.0
234. BD*(1) C13-H15	3.0
203. BD*(1) Si1-C9	2.4

Figure S46. Selected NBOs of **4** , their atomic orbital compositions and five largest donor-acceptor interactions, according to the second-order perturbation theory.

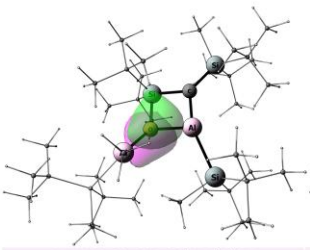

NLMO 69. 97.0% LP(1)O7
97.0% O7 s(0.9%)p(99.1%)d(0.0%)

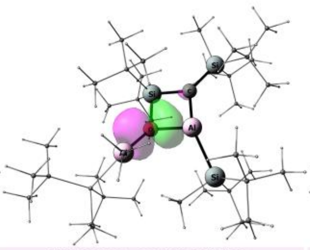

NLMO 70. 96.4% LP(2)O7
96.4% O7 s(1.0%)p(99.0%)d(0.0%)
1.7% Al5 s(62.4%)p(31.2%)d(6.0%)

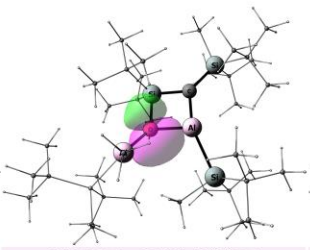

NLMO 71. 94.1% LP(3)O7
94.2% O7 s(17.0%)p(83.0%)d(0.1%)
2.0% Al4 s(31.1%)p(65.8%)d(3.1%)
1.9% Al5 s(31.8%)p(63.3%)d(4.7%)
1.2% Si2 s(43.5%)p(49.3%)d(6.8%)

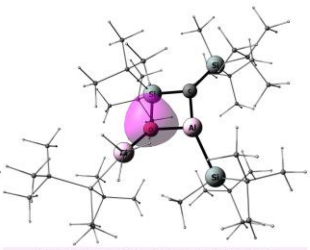

NLMO 76. 95.8% BD(1)Si2-O7
87.7% O7 s(80.9%)p(19.0%)d(0.1%)
8.4% Si2 s(22.5%)p(75.9%)d(1.5%)
1.8% Al5 s(20.6%)p(78.5%)d(0.8%)
1.6% Al4 s(14.9%)p(84.1%)d(0.9%)

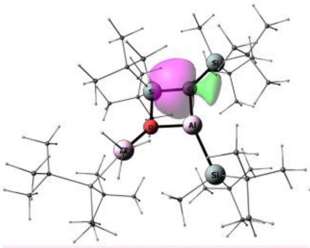

NLMO 77. 96.6% BD(1)Si2-C8
74.7% C8 s(32.6%)p(67.1%)d(0.3%)
22.6% Si2 s(47.3%)p(52.1%)d(0.6%)

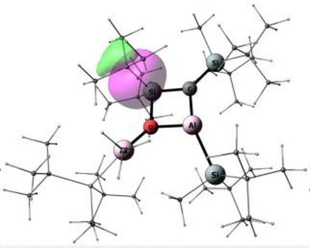

NLMO 78. 95.6% BD(1)Si2-C10
73.7% C10 s(23.6%)p(76.2%)d(0.2%)
22.8% Si2 s(46.8%)p(52.6%)d(0.6%)

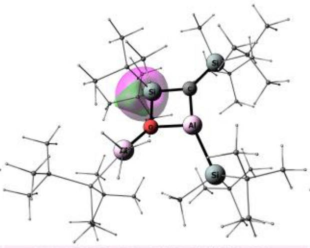

NLMO 79. 95.6% BD(1)Si2-C17
73.6% C17 s(23.4%)p(76.4%)d(0.2%)
22.9% Si2 s(46.8%)p(52.6%)d(0.6%)

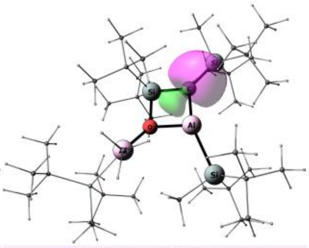

NLMO 80. 97.0% BD(1)Si3-C8
74.5% C8 s(37.1%)p(62.7%)d(0.1%)
23.0% Si3 s(38.6%)p(60.9%)d(0.5%)

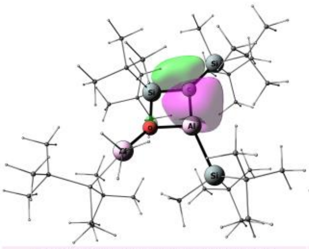

NLMO 84. 93.5% BD(1)Al4-C8
82.5% C8 s(19.1%)p(80.7%)d(0.2%)
12.0% Al4 s(72.6%)p(26.9%)d(0.4%)
2.6% Si2 s(0.5%)p(95.2%)d(4.3%)
1.3% Si3 s(3.8%)p(86.1%)d(9.9%)

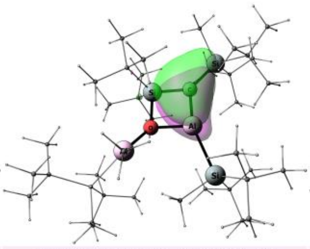

NLMO 85. 90.5% BD(2)Al4-C8
79.7% C8 s(0.0%)p(99.8%)d(0.1%)
10.8% Al4 s(0.0%)p(98.4%)d(1.4%)
4.4% Si2 s(0.0%)p(93.2%)d(6.1%)
2.0% Si3 s(0.1%)p(87.6%)d(12.1%)

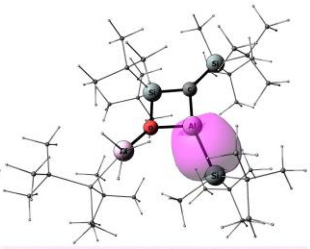

NLMO 72. 94.4% BD(1)Si1-Al4
63.3% Si1 s(34.1%)p(64.9%)d(0.9%)
33.6% Al4 s(70.4%)p(29.4%)d(0.1%)

Figure S47. Selected NLMOs of **4** and compositions.

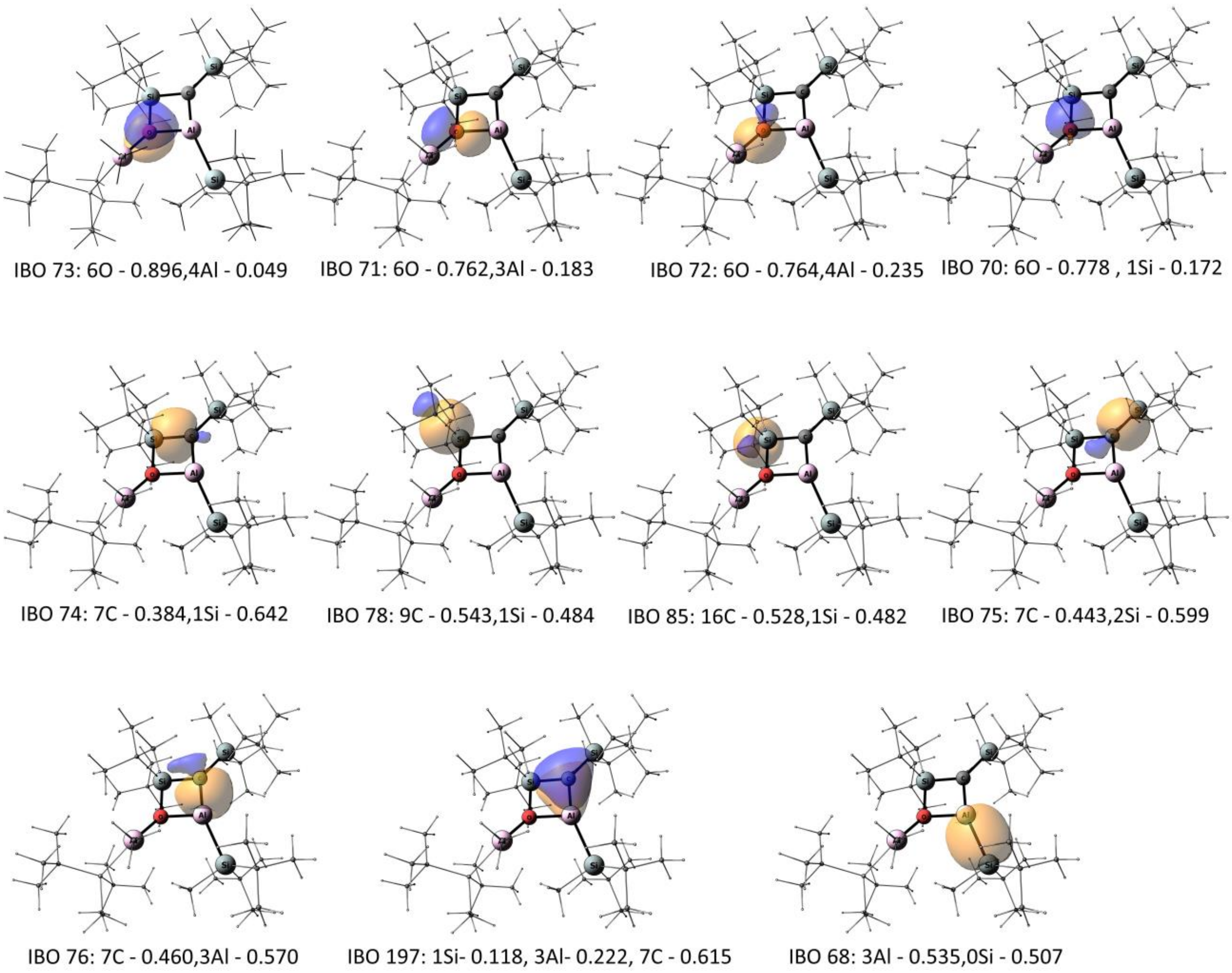

Figure S48. Selected Intrinsic Bond Orbitals of **4** and their Mulliken populations on each atom. Strongly localized orbitals have populations of >=0.950 on one atom; two center bond orbitals have populations of >=0.850 on two atoms; other orbitals are considered to be 'delocalized'.

The picture obtained from the IBO calculation is essentially similar to that obtained from the NBO/NLMO analysis. The bond like localized orbital IBO 76 corresponds to the σ(Al-C) bond while the IBO 197 is the π(Al-C) delocalized to the geminal Si center, due to the favorable interactions with the σ*(Si-C) orbitals.

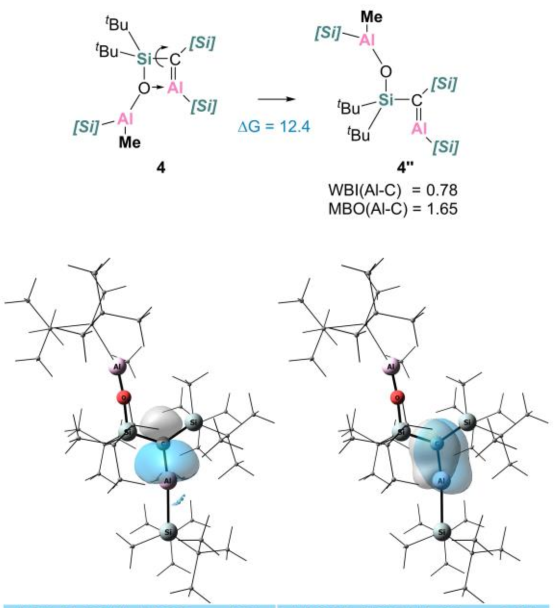

NBO 84. BD(1) Al4-C8 occ. 1.88		NBO 85. BD(2) Al4-C8 occ. 1.85	
12.37% Al4 sp0.79		13.01% Al4 sp99.99	
87.63% C8 sp3.51		86.99% C8 sp1.00	
Acceptor	E(2)(kcal mol^{-1})	Acceptor	E(2)(kcal mol^{-1})
202. BD*(1) Si1-Al4	20.9	212. BD*(1) Si3-C31	7.3
206. BD*(1) Si2-O7	5.7	209. BD*(1) Si2-C17	6.2
385. RY (1) Si3	5.6	208. BD*(1) Si2-C10	5.4
357. RY (1) Si2	4.5	211. BD*(1) Si3-C26	4.6
213. BD*(1) Si3-C44	4.3	386. RY (2) Si3	3.7

Figure S49. Conversion of the cyclic **4** to the open chain **4''**, and the selected NBOs of **4''**.

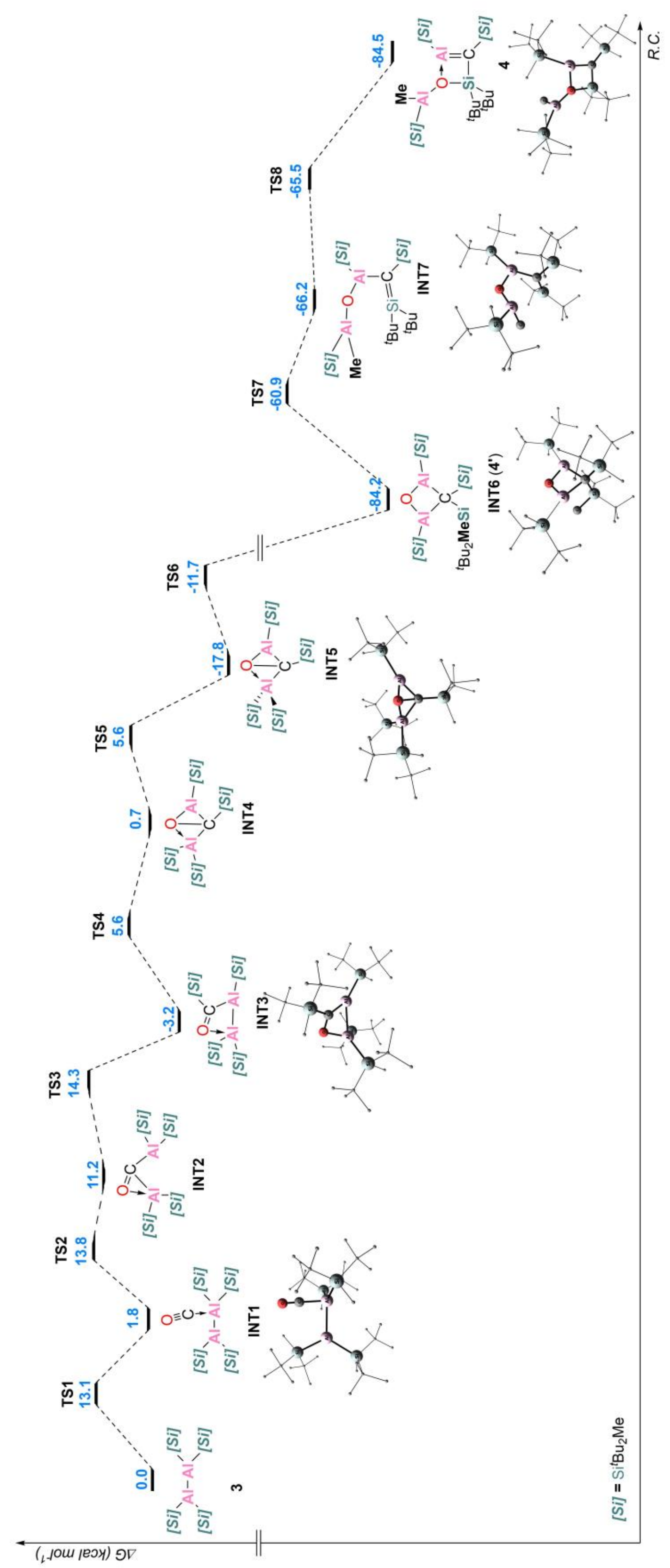

Figure S50. Free energy reaction coordinate diagram for the proposed mechanism of the formation of **4** from **3** in the presence of CO at the (SMD=Benzene)PW6B95-D4/def2-QZVPP//r^{2}SCAN-3c level of theory.

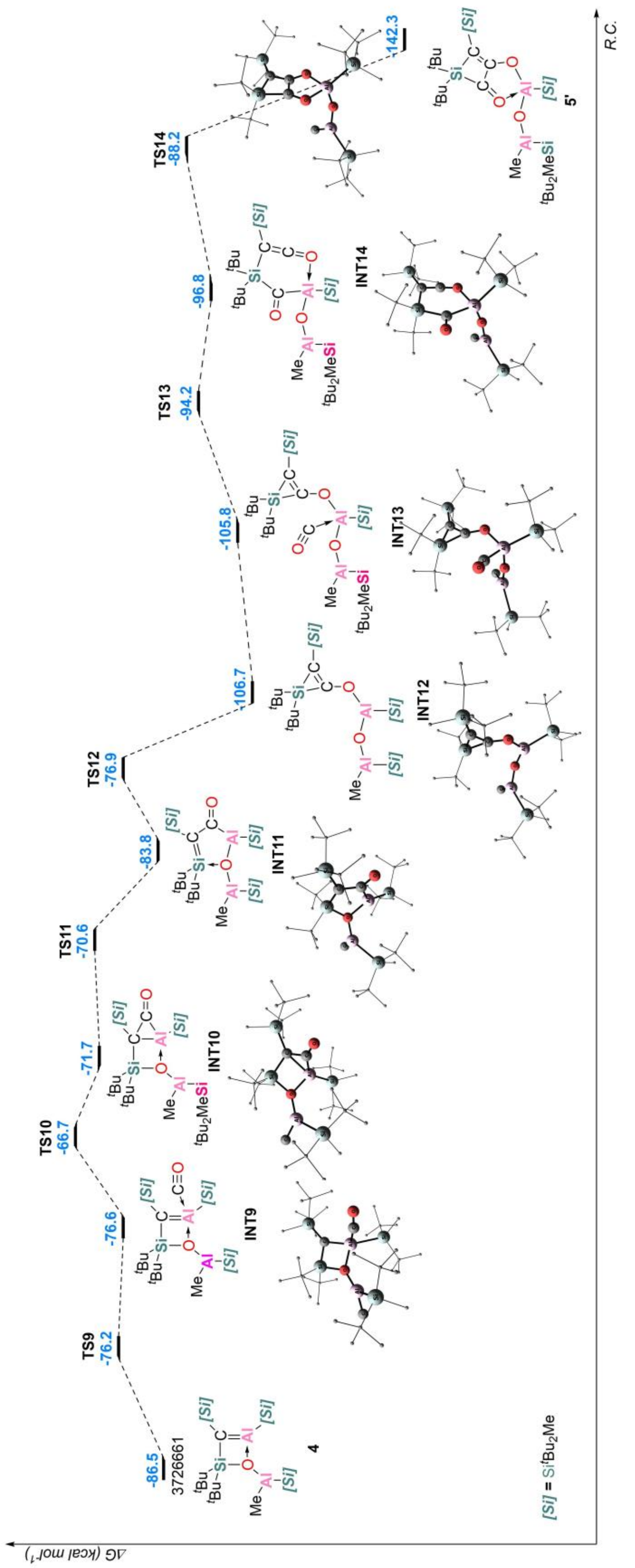

Figure S51. Free energy reaction coordinate diagram for the proposed mechanism of the formation of **5** from **4** in the presence of two equivalents of CO at the (SMD=Benzene)PW6B95-D4/def2-QZVPP//r^{2}SCAN-3c level of theory.

Cartesian coordinates and energies of the optimized geometries at the r2SCAN-3c level.

Calculated energies and coordinates of **3**

Electronic energy	...	-3064.81982721 Eh
Total Enthalpy	...	-3063.62889961 Eh
Final Gibbs free energy	...	-3063.78150549 Eh

CARTESIAN COORDINATES (ANGSTROEM)

Si	3.450861	12.075013	13.196390
Al	5.222754	10.878661	11.832829
Al	5.908443	8.501895	12.676088
Si	7.775853	8.371845	14.376350
Si	6.432447	11.998394	9.902743
Si	4.630017	6.456550	11.925982
C	2.459044	14.038732	11.327755
H	2.628899	14.887037	11.999147
H	1.679895	14.339616	10.611802
H	3.381894	13.865755	10.763434
C	2.020857	12.782533	12.089110
C	6.886536	8.025198	16.074041
C	5.525173	8.736182	16.013270
H	4.989438	8.644533	16.970436
H	4.876509	8.287332	15.245169
H	5.628148	9.804595	15.793597
C	0.765491	13.104451	12.917552
H	0.377881	12.217242	13.429312
H	-0.030201	13.474780	12.254078
H	0.948132	13.875371	13.671912
C	1.641739	11.700799	11.064356
H	2.485241	11.444845	10.412000
H	0.825652	12.058628	10.419157
H	1.297802	10.780247	11.547681
C	5.903607	11.229292	8.198452
C	6.218630	12.179100	7.029921
H	5.677842	13.126881	7.115742
H	5.916041	11.711800	6.081086
H	7.286886	12.407437	6.957790
C	4.381538	11.014843	8.238569
H	4.094119	10.287014	9.008966
H	4.021733	10.626008	7.274298
H	3.841042	11.947872	8.440329
C	6.585262	9.881692	7.934124
H	7.655349	10.001994	7.735912
H	6.140345	9.397907	7.051964
H	6.473479	9.191973	8.777082
C	8.334522	11.878747	10.281665
C	8.781400	10.443799	10.588512
H	8.199074	10.011202	11.412384
H	9.838279	10.430913	10.893493
H	8.680147	9.778881	9.725656
C	8.593573	12.734594	11.534142
H	8.350808	13.790237	11.371077
H	9.653716	12.676132	11.821589
H	8.008303	12.383482	12.395356
C	9.191938	12.425887	9.129441
H	9.116225	11.801296	8.233236
H	10.251199	12.442738	9.426147
H	8.913741	13.449511	8.855248
C	6.046752	13.855826	9.745011
H	5.057174	14.013693	9.303797
H	6.774772	14.349743	9.089975
H	6.059941	14.369450	10.709553
C	8.591240	10.081848	14.564026
H	8.987796	10.447309	13.612473
H	9.418295	10.058287	15.283849
H	7.869272	10.824340	14.922106
C	7.677469	8.600414	17.259853
H	7.147802	8.387562	18.200351
H	7.790830	9.686913	17.185015
H	8.678361	8.163417	17.339589
C	6.634361	6.532828	16.316354
H	7.564701	5.981994	16.487266
H	6.116284	6.067059	15.470814
H	6.005200	6.397337	17.208549
C	9.210218	7.136631	13.953289
C	8.685219	5.739853	13.598606
H	9.518524	5.087327	13.298656
H	7.982326	5.789270	12.761176
H	8.170674	5.257836	14.434329
C	10.206399	7.018463	15.118016
H	9.751919	6.556719	16.000749
H	10.612731	7.991975	15.414687
H	11.056077	6.386198	14.820124
C	9.957166	7.684259	12.725277
H	10.409941	8.662189	12.919461
H	9.292896	7.786375	11.857736
H	10.765148	6.994491	12.441013
C	5.198403	6.113849	10.096900
C	6.707112	6.404230	10.012382
H	6.940670	7.450213	10.265194
H	7.077109	6.235024	8.989933
H	7.287561	5.760449	10.682649
C	4.471823	7.021438	9.098089
H	3.412316	6.761806	9.007824
H	4.921225	6.925268	8.098724
H	4.536751	8.076682	9.385220
C	4.971658	4.647683	9.693871
H	5.540706	3.958749	10.326512
H	5.298861	4.490108	8.655389
H	3.915810	4.363228	9.752927
C	5.029036	4.873701	12.902801
H	6.063535	4.553478	12.745602
H	4.377103	4.048683	12.590448
H	4.892844	5.013699	13.978762
C	2.719720	6.736044	12.133390
C	2.418388	6.741399	13.642650
H	2.656901	5.783118	14.115640
H	1.349766	6.938659	13.811791
H	2.977757	7.527336	14.166306
C	2.263551	8.084227	11.561439
H	2.828230	8.914124	12.005118
H	1.199522	8.249830	11.787994
H	2.380878	8.145927	10.475567
C	1.901178	5.611436	11.478671
H	2.010495	5.605069	10.389290
H	0.832056	5.749237	11.698373
H	2.186079	4.622233	11.854248
C	2.608989	10.856684	14.394492
H	1.927522	10.184137	13.862361
H	2.020365	11.387653	15.152937
H	3.338437	10.231636	14.918312
C	4.339071	13.400517	14.308565
C	5.174105	12.636365	15.348404
H	4.548047	12.051633	16.030643
H	5.763860	13.339633	15.954347
H	5.880831	11.948352	14.865036
C	5.293982	14.282649	13.493371
H	6.064776	13.682972	12.994484
H	5.809085	14.996207	14.153456
H	4.772666	14.858925	12.723358
C	3.342854	14.302434	15.052858
H	2.798944	14.960478	14.367617

H 3.881120 14.946219 15.764546
H 2.608405 13.724409 15.625236

Calculated energies and coordinates of **4**

Electronic energy ... -3178.25666114 Eh
Total Enthalpy ... -3177.05552272 Eh
Final Gibbs free energy ... -3177.20953113 Eh

CARTESIAN COORDINATES (ANGSTROEM)
Si 15.862018 5.736818 19.602607
Si 12.816963 4.293096 15.999239
Si 10.638306 4.490877 18.576423
Al 13.950987 5.007061 18.182589
Al 16.132455 4.122109 15.791814
Si 17.674198 5.286361 14.204504
O 14.547593 4.632205 16.395145
C 12.212754 4.620385 17.661045
C 15.696964 7.619810 20.000308
C 12.864452 2.472759 15.339517
C 16.238531 4.592753 21.120471
C 17.436898 7.190357 13.932839
C 17.343321 5.548586 18.412333
H 17.645468 4.500863 18.314351
H 18.222806 6.107210 18.755983
H 17.105234 5.942628 17.413649
C 12.388622 5.575215 14.626457
C 16.824073 2.505435 16.682238
H 16.653488 1.599956 16.087099
H 16.338877 2.335204 17.650932
H 17.905131 2.567231 16.855117
C 15.874879 3.146894 20.744338
H 14.799237 3.031304 20.564182
H 16.148087 2.464227 21.561932
H 16.409844 2.809296 19.848568
C 10.900416 3.484720 20.227107
C 17.739760 4.640591 21.459870
H 18.359193 4.270635 20.636169
H 17.939509 4.001839 22.332188
H 18.078379 5.651744 21.709005
C 9.861881 6.248986 18.926147
C 16.893922 8.138292 20.813017
H 16.929550 7.699184 21.815218
H 16.814531 9.228017 20.937500
H 17.851231 7.936105 20.318431
C 13.953960 2.216055 14.290582
H 13.792110 2.764858 13.362119
H 13.998534 1.145219 14.042682
H 14.967847 2.457497 14.653960
C 13.114370 1.540652 16.534514
H 14.048250 1.778750 17.057461
H 13.180247 0.498193 16.190045
H 12.305772 1.615139 17.263712
C 9.317250 3.527114 17.607425
H 9.242976 3.883075 16.574029
H 8.323709 3.622471 18.060666
H 9.559269 2.458403 17.572308
C 13.134086 5.340411 13.306455
H 14.213443 5.214053 13.456514
H 12.997815 6.201816 12.635847
H 12.759821 4.459790 12.774929
C 15.441126 4.984213 22.372706
H 15.744955 5.960894 22.762590
H 15.620155 4.246159 23.167804
H 14.362530 5.010356 22.188085
C 16.487599 4.055901 11.872359
H 16.041336 5.010408 11.576997
H 15.763675 3.531167 12.504404
H 16.620846 3.455230 10.960906
C 14.397841 7.920354 20.760421
H 13.520608 7.587793 20.196971
H 14.296638 9.004148 20.915966
H 14.365730 7.441882 21.743241
C 18.344156 2.833633 12.991575
H 18.386517 2.181943 12.107245
H 17.682983 2.348323 13.721827
H 19.349511 2.871513 13.423494
C 10.876289 5.561316 14.344827
H 10.557224 4.639473 13.849223
H 10.611137 6.398507 13.682920
H 10.290635 5.667203 15.261337
C 17.834840 4.228822 12.587693
C 19.323376 5.090305 15.143256
H 19.378104 5.773865 15.997539
H 20.177652 5.306149 14.490332
H 19.451360 4.075190 15.531444
C 12.774255 6.966110 15.159545
H 12.274013 7.186965 16.107760
H 12.491358 7.738539 14.430055
H 13.855093 7.048063 15.324741
C 11.511854 2.095120 14.710174
H 10.674950 2.313668 15.381894
H 11.488262 1.017302 14.493908
H 11.337811 2.620946 13.765929
C 11.710763 4.303980 21.239187
H 12.628044 4.706344 20.784391
H 12.017526 3.678632 22.091747
H 11.141510 5.149653 21.638503
C 9.577442 3.040977 20.875068
H 8.950171 3.880467 21.182309
H 9.784733 2.439430 21.772941
H 8.989944 2.415641 20.193575
C 17.024851 7.813168 15.275779
H 16.079911 7.399601 15.647404
H 16.886570 8.898719 15.165992
H 17.791343 7.660845 16.044683
C 11.692801 2.205068 19.915668
H 11.142972 1.544794 19.234527
H 11.873525 1.640476 20.842894
H 12.659505 2.429444 19.453620
C 15.652302 8.373116 18.660228
H 16.587459 8.272475 18.100151
H 15.486659 9.445082 18.838366
H 14.831946 8.021691 18.018019
C 18.760113 7.846949 13.498736
H 19.551785 7.701009 14.240958
H 18.610576 8.930552 13.386380
H 19.121695 7.465168 12.539632
C 10.916379 7.245251 19.422368
H 11.323090 6.965331 20.399296
H 10.478335 8.249776 19.524583
H 11.749191 7.311688 18.711964
C 16.357243 7.517104 12.893278
H 16.629383 7.163327 11.893462
H 16.222004 8.606436 12.828182
H 15.387736 7.083206 13.156978
C 18.841583 4.846717 11.604216
H 19.815796 5.035697 12.069382
H 18.475158 5.788935 11.184187
H 19.007985 4.158169 10.762911
C 8.707395 6.213331 19.939126
H 7.937002 5.482661 19.666198
H 8.221310 7.199504 19.985056
H 9.054925 5.979527 20.950233
C 9.304782 6.801704 17.603638
H 10.100287 6.928787 16.863435
H 8.853456 7.790988 17.772265
H 8.531076 6.155871 17.173234

Calculated energies and coordinates of **TS1**

Electronic energy ... -3178.10342772 Eh
Total Enthalpy ... -3176.90288351 Eh
Final Gibbs free energy ... -3177.06012950 Eh

CARTESIAN COORDINATES (ANGSTROEM)
Si 1.639449 -0.403834 2.720189
Si -0.206494 -2.288128 -2.623790
Si -2.772361 0.802983 1.637548

Al	-0.313290	0.222910	1.131465
Al	0.461773	-0.143558	-1.393087
Si	2.050347	1.524935	-2.472764
O	0.639903	4.777235	1.073581
C	0.459120	3.663972	0.980073
C	2.251585	1.171650	3.688476
C	-0.606511	-3.616520	-1.265740
C	1.383021	-1.945341	3.886030
C	1.093407	2.911696	-3.443450
C	3.063852	-0.887230	1.546445
H	2.899014	-1.876902	1.105186
H	4.030472	-0.913850	2.064964
H	3.148977	-0.171160	0.721630
C	-1.681860	-1.874671	-3.821114
C	1.115528	-3.129538	-3.707274
H	0.650176	-3.943123	-4.278398
H	1.917252	-3.569011	-3.105566
H	1.581485	-2.450268	-4.424103
C	0.766587	-3.092291	3.070360
H	-0.160006	-2.809600	2.559995
H	0.534256	-3.940693	3.730939
H	1.465414	-3.453542	2.310433
C	-3.447886	-0.232824	3.133980
C	2.726063	-2.473337	4.426843
H	3.421930	-2.725180	3.619401
H	2.545597	-3.394606	5.000599
H	3.222708	-1.767179	5.095091
C	-3.151681	2.706333	1.765725
C	3.286019	0.861067	4.781274
H	2.852326	0.305034	5.617839
H	3.681466	1.803885	5.187904
H	4.136902	0.289894	4.393264
C	-0.853809	-5.021901	-1.839509
H	-1.755966	-5.072048	-2.454199
H	-0.979000	-5.739097	-1.014864
H	-0.009859	-5.368122	-2.445596
C	0.631864	-3.696408	-0.360904
H	1.530665	-3.998811	-0.910293
H	0.468089	-4.433813	0.437326
H	0.838268	-2.732837	0.124526
C	-3.765301	0.210652	0.127892
H	-3.352941	0.613476	-0.800698
H	-4.814688	0.522455	0.193275
H	-3.750487	-0.881148	0.045978
C	-2.497981	-3.114173	-4.216489
H	-1.868497	-3.907572	-4.635173
H	-3.236870	-2.841539	-4.984769
H	-3.050692	-3.529262	-3.367652
C	0.469688	-1.623497	5.076627
H	0.914646	-0.877531	5.743178
H	0.297632	-2.531788	5.672684
H	-0.508873	-1.249890	4.761064
C	2.823677	0.142834	-4.883877
H	2.677238	1.003162	-5.545661
H	1.862701	-0.373282	-4.788972
H	3.523002	-0.542131	-5.385900
C	1.068226	1.911411	4.326700
H	0.334273	2.210111	3.571291
H	1.413306	2.822719	4.837119
H	0.542588	1.295007	5.063880
C	3.756184	-0.689577	-2.716189
H	4.530841	-1.264649	-3.245185
H	2.895034	-1.354568	-2.577332
H	4.154728	-0.438806	-1.725278
C	-2.628863	-0.854568	-3.178273
H	-3.094588	-1.245903	-2.269649
H	-3.434472	-0.577497	-3.874499
H	-2.105450	0.073391	-2.906279
C	3.376988	0.568123	-3.518768
C	3.064312	2.431938	-1.142597
H	2.428549	2.836283	-0.351822
H	3.627167	3.265672	-1.579635
H	3.787328	1.762089	-0.665227
C	-1.092303	-1.261730	-5.101130
H	-0.453942	-0.399671	-4.883231
H	-1.902580	-0.913615	-5.758487
H	-0.496305	-1.987715	-5.663939
C	-1.803320	-3.204756	-0.403235
H	-1.659442	-2.199356	0.018102
H	-1.931785	-3.900674	0.439253
H	-2.739691	-3.194714	-0.972579
C	-3.008990	0.368789	4.475424
H	-1.933347	0.582859	4.498589
H	-3.228545	-0.327438	5.298402
H	-3.539373	1.302755	4.689064
C	-4.980196	-0.365857	3.115161
H	-5.489946	0.597103	3.204583
H	-5.305820	-0.990144	3.960572
H	-5.331883	-0.850033	2.197980
C	0.371564	3.785480	-2.405218
H	-0.278310	3.186778	-1.753104
H	-0.262893	4.528455	-2.910232
H	1.078793	4.331498	-1.772779
C	-2.862740	-1.646357	3.004379
H	-3.183426	-2.138030	2.078725
H	-3.185961	-2.278568	3.844868
H	-1.768396	-1.626104	3.014266
C	2.924323	2.102770	2.668970
H	3.861516	1.683852	2.287918
H	3.154388	3.074433	3.130636
H	2.277062	2.290541	1.806341
C	2.043165	3.810138	-4.251901
H	2.836268	4.239052	-3.629140
H	1.481370	4.648532	-4.690007
H	2.515210	3.268368	-5.077735
C	-2.284241	3.413662	2.813766
H	-2.493012	3.064822	3.829257
H	-2.463621	4.498754	2.789285
H	-1.219035	3.252017	2.630427
C	0.037273	2.331719	-4.391623
H	0.480009	1.710543	-5.175952
H	-0.516153	3.143910	-4.885392
H	-0.695340	1.718552	-3.852805
C	4.655006	1.395599	-3.736421
H	5.135813	1.662541	-2.789735
H	4.463231	2.320777	-4.288815
H	5.380990	0.808840	-4.318557
C	-4.626263	2.974269	2.111741
H	-5.316535	2.449920	1.441798
H	-4.836196	4.050502	2.022280
H	-4.860920	2.683746	3.140535
C	-2.869193	3.319840	0.382634
H	-1.863515	3.073534	0.020578
H	-2.946777	4.415951	0.429602
H	-3.584454	2.968646	-0.368292

Calculated energies and coordinates of **INT1**

Electronic energy ... -3178.13243115 Eh
Total Enthalpy ... -3176.92947409 Eh
Final Gibbs free energy ... -3177.08520351 Eh

CARTESIAN COORDINATES (ANGSTROEM)

Si	1.691667	-0.249146	2.705593
Si	-0.244334	-2.251455	-2.628317
Si	-2.826938	0.816850	1.661850
Al	-0.333768	0.312789	1.176214
Al	0.473187	-0.117416	-1.382333
Si	2.109722	1.527252	-2.452788
O	0.387549	3.312985	0.698265
C	0.152322	2.206549	0.901719
C	2.301051	1.296474	3.723767
C	-0.664709	-3.594193	-1.294585
C	1.426708	-1.826739	3.818110
C	1.190420	2.901209	-3.480820
C	3.086808	-0.695546	1.490819
H	2.895911	-1.657553	1.001054
H	4.058760	-0.765522	1.995135
H	3.173213	0.063205	0.706277

C	-1.702806	-1.798245	-3.829748
C	1.075691	-3.092707	-3.713840
H	0.598441	-3.891933	-4.295408
H	1.866786	-3.552643	-3.113624
H	1.555004	-2.413820	-4.421349
C	0.819435	-2.947332	2.961427
H	-0.089016	-2.643089	2.432462
H	0.560318	-3.807030	3.596689
H	1.534481	-3.299804	2.212640
C	-3.440821	-0.262023	3.155769
C	2.766555	-2.373358	4.351021
H	3.473522	-2.586333	3.541824
H	2.580164	-3.321239	4.877152
H	3.251757	-1.699642	5.059014
C	-3.266162	2.706604	1.832744
C	3.289040	0.917153	4.840529
H	2.817228	0.337182	5.638782
H	3.685964	1.835263	5.298147
H	4.144063	0.348000	4.458587
C	-0.916474	-4.991043	-1.885259
H	-1.817413	-5.029850	-2.502884
H	-1.048872	-5.715733	-1.068226
H	-0.072749	-5.336710	-2.491934
C	0.570935	-3.687079	-0.386940
H	1.462939	-4.014405	-0.933036
H	0.393806	-4.410003	0.421981
H	0.794175	-2.721174	0.084431
C	-3.809655	0.219537	0.151710
H	-3.418017	0.651447	-0.772797
H	-4.866645	0.501247	0.232499
H	-3.762485	-0.869691	0.053123
C	-2.547095	-3.020088	-4.222124
H	-1.935229	-3.826340	-4.642218
H	-3.281158	-2.731605	-4.989171
H	-3.106441	-3.423658	-3.372176
C	0.503410	-1.541929	5.010248
H	0.936427	-0.810556	5.700047
H	0.331569	-2.466726	5.580216
H	-0.474274	-1.166296	4.696326
C	2.890325	0.090700	-4.836662
H	2.808871	0.947795	-5.513471
H	1.901340	-0.374132	-4.768200
H	3.569385	-0.635791	-5.307164
C	1.117344	2.043080	4.355857
H	0.410282	2.393930	3.597792
H	1.474372	2.922253	4.911484
H	0.560273	1.410861	5.056370
C	3.741138	-0.752426	-2.638283
H	4.512263	-1.355902	-3.140186
H	2.856541	-1.390235	-2.519719
H	4.117768	-0.505306	-1.637813
C	-2.624684	-0.755651	-3.186564
H	-3.092958	-1.136161	-2.274587
H	-3.427918	-0.463590	-3.879300
H	-2.080466	0.162130	-2.919876
C	3.423747	0.508971	-3.461383
C	3.153783	2.455780	-1.158284
H	2.556159	2.953380	-0.394050
H	3.759266	3.223771	-1.654541
H	3.848635	1.779422	-0.649260
C	-1.097313	-1.205179	-5.111578
H	-0.430524	-0.364372	-4.897669
H	-1.897862	-0.831871	-5.766881
H	-0.526869	-1.950793	-5.675133
C	-1.865197	-3.182517	-0.436123
H	-1.717100	-2.186406	-0.000024
H	-2.005136	-3.890546	0.394519
H	-2.796186	-3.161495	-1.014149
C	-2.984550	0.334277	4.494045
H	-1.914075	0.573941	4.491557
H	-3.165989	-0.376965	5.313336
H	-3.529343	1.253301	4.733713
C	-4.971459	-0.426983	3.165316
H	-5.502576	0.518719	3.295456
H	-5.260547	-1.083561	3.999189
H	-5.332446	-0.894970	2.243221
C	0.423449	3.795688	-2.493893
H	-0.314019	3.222281	-1.918234
H	-0.125158	4.574417	-3.043524
H	1.087726	4.296533	-1.783163
C	-2.838651	-1.664608	2.999539
H	-3.164089	-2.147251	2.071066
H	-3.147389	-2.309365	3.835616
H	-1.746262	-1.632693	2.996377
C	3.048953	2.247746	2.775259
H	3.976171	1.800275	2.401895
H	3.317628	3.169474	3.311038
H	2.453220	2.539132	1.908600
C	2.181579	3.779321	-4.261780
H	2.937978	4.230439	-3.610160
H	1.641833	4.602109	-4.753808
H	2.699651	3.216632	-5.045105
C	-2.380347	3.453839	2.839829
H	-2.445111	3.025880	3.844577
H	-2.697255	4.504860	2.905822
H	-1.328835	3.453460	2.548148
C	0.171002	2.316442	-4.465500
H	0.638125	1.685773	-5.227306
H	-0.357468	3.128127	-4.986747
H	-0.587380	1.715214	-3.949523
C	4.735805	1.286971	-3.660315
H	5.217232	1.529451	-2.707766
H	4.585422	2.222398	-4.208583
H	5.443610	0.675415	-4.239256
C	-4.730616	2.892032	2.270747
H	-5.431945	2.331690	1.642513
H	-5.001361	3.955434	2.194633
H	-4.886496	2.593201	3.311746
C	-3.116240	3.357348	0.444821
H	-2.134059	3.179458	-0.004913
H	-3.245031	4.445899	0.528357
H	-3.873151	2.988323	-0.254970

Calculated energies and coordinates of **TS2**

Electronic energy ... -3178.11812355 Eh
Total Enthalpy ... -3176.91612185 Eh
Final Gibbs free energy ... -3177.07002819 Eh

CARTESIAN COORDINATES (ANGSTROEM)

Si	1.652935	-0.018435	2.707444
Si	-0.435332	-2.151131	-2.719007
Si	-2.915340	0.650240	1.696407
Al	-0.398328	0.252313	1.231847
Al	0.502399	-0.135695	-1.432725
Si	2.359072	1.413426	-2.342289
O	-0.954127	2.077947	-1.171053
C	-0.564839	1.609637	-0.155341
C	1.984662	1.605288	3.710279
C	-0.899122	-3.495053	-1.398213
C	1.661010	-1.646602	3.772809
C	1.718858	2.875114	-3.469815
C	3.079005	-0.247883	1.478345
H	3.074754	-1.263952	1.067997
H	4.058131	-0.075788	1.941779
H	2.975050	0.442359	0.640889
C	-1.875891	-1.597537	-3.891288
C	0.830291	-3.049916	-3.822871
H	0.302419	-3.811568	-4.410639
H	1.591819	-3.565874	-3.229332
H	1.348926	-2.395904	-4.524020
C	1.184060	-2.818669	2.900886
H	0.162739	-2.683165	2.531506
H	1.200948	-3.750191	3.485351
H	1.833370	-2.968649	2.031883
C	-3.370453	-0.497065	3.203846
C	3.100808	-1.974507	4.215704
H	3.772906	-2.098078	3.360464
H	3.100518	-2.923492	4.771708

H	3.525768	-1.211138	4.872200
C	-3.366104	2.528488	2.004037
C	3.217353	1.491667	4.621703
H	3.041103	0.810762	5.460546
H	3.451318	2.477861	5.048642
H	4.108245	1.148776	4.083190
C	-1.145397	-4.878814	-2.022243
H	-1.988731	-4.879859	-2.718398
H	-1.374512	-5.604076	-1.227475
H	-0.264463	-5.248883	-2.556685
C	0.297989	-3.614195	-0.441543
H	1.215786	-3.918331	-0.959116
H	0.094099	-4.364682	0.336636
H	0.496585	-2.664664	0.071524
C	-4.005647	0.112613	0.246195
H	-3.665424	0.560402	-0.691744
H	-5.041426	0.434839	0.410678
H	-4.009420	-0.973898	0.119810
C	-2.734736	-2.782006	-4.361028
H	-2.133071	-3.571769	-4.825041
H	-3.460217	-2.438954	-5.113402
H	-3.305029	-3.225890	-3.538735
C	0.773820	-1.560253	5.020442
H	1.141857	-0.812731	5.730682
H	0.766603	-2.528772	5.541356
H	-0.263635	-1.312379	4.775431
C	3.024591	-0.197389	-4.635443
H	3.119589	0.622088	-5.353975
H	1.966073	-0.474043	-4.593563
H	3.580815	-1.055927	-5.039717
C	0.772755	1.994442	4.565355
H	-0.123509	2.109390	3.950395
H	0.955053	2.956251	5.066935
H	0.555209	1.253585	5.340851
C	3.671273	-1.070862	-2.380218
H	4.369477	-1.792320	-2.830411
H	2.704355	-1.584454	-2.282810
H	4.036998	-0.840466	-1.372161
C	-2.783797	-0.572772	-3.202137
H	-3.272629	-0.993068	-2.318818
H	-3.573164	-0.234211	-3.889415
H	-2.224309	0.317521	-2.885383
C	3.565469	0.192460	-3.253451
C	3.420090	2.295965	-1.030925
H	2.813118	2.765349	-0.251343
H	4.003609	3.087170	-1.517414
H	4.132822	1.622335	-0.547810
C	-1.241181	-0.938271	-5.126600
H	-0.573384	-0.115624	-4.848913
H	-2.026302	-0.519949	-5.772878
H	-0.666614	-1.654657	-5.723251
C	-2.128160	-3.082724	-0.584493
H	-1.988708	-2.091486	-0.135516
H	-2.301501	-3.799061	0.232362
H	-3.036408	-3.053865	-1.196823
C	-2.830587	0.075381	4.521244
H	-1.761650	0.308866	4.455380
H	-2.961502	-0.652833	5.335326
H	-3.350624	0.991644	4.816130
C	-4.889088	-0.720207	3.321538
H	-5.439723	0.199702	3.529523
H	-5.090768	-1.418198	4.147516
H	-5.303455	-1.164946	2.410356
C	1.255736	4.041613	-2.576594
H	0.463881	3.753350	-1.883073
H	0.859947	4.847891	-3.211150
H	2.083027	4.460903	-1.995039
C	-2.729979	-1.871693	2.968324
H	-3.097478	-2.342200	2.050514
H	-2.960527	-2.546826	3.805502
H	-1.639508	-1.802440	2.896136
C	2.240727	2.723232	2.686394
H	3.160215	2.548815	2.117519
H	2.345167	3.688738	3.201582
H	1.411888	2.818289	1.971460
C	2.850293	3.418799	-4.361187
H	3.722528	3.733173	-3.776955
H	2.488546	4.304271	-4.904531
H	3.183583	2.694274	-5.109346
C	-2.321774	3.258125	2.857016
H	-2.220441	2.816802	3.853268
H	-2.613886	4.309914	2.992134
H	-1.336947	3.249529	2.375373
C	0.552160	2.447352	-4.368706
H	0.846314	1.655327	-5.065170
H	0.203118	3.299794	-4.969897
H	-0.300937	2.089378	-3.781495
C	4.982636	0.767579	-3.412650
H	5.442152	0.992434	-2.444520
H	4.996416	1.682754	-4.011598
H	5.626292	0.032728	-3.918680
C	-4.734226	2.645735	2.701573
H	-5.527783	2.129586	2.149332
H	-5.017469	3.706809	2.761343
H	-4.719234	2.258549	3.724045
C	-3.482328	3.270311	0.660343
H	-2.533835	3.311911	0.122781
H	-3.795227	4.307148	0.850332
H	-4.229377	2.819141	-0.000472

Calculated energies and coordinates of **INT2**

Electronic energy ... -3178.12044958 Eh
Total Enthalpy ... -3176.91790239 Eh
Final Gibbs free energy ... -3177.07323359 Eh

CARTESIAN COORDINATES (ANGSTROEM)

Si	1.697755	-0.065258	2.788399
Si	-0.520991	-2.098471	-2.701179
Si	-2.878889	0.633709	1.669225
Al	-0.358085	0.207589	1.348856
Al	0.383408	0.002888	-1.589428
Si	2.336682	1.447264	-2.354427
O	-0.919605	1.566328	-1.333133
C	-0.576901	1.330712	-0.171287
C	2.044092	1.551782	3.791016
C	-0.934968	-3.439903	-1.361762
C	1.677552	-1.697953	3.839219
C	1.640881	2.911672	-3.441674
C	3.089142	-0.296725	1.522197
H	3.063796	-1.305841	1.096509
H	4.079598	-0.142071	1.967497
H	2.973669	0.404883	0.694987
C	-1.956594	-1.663588	-3.932094
C	0.817720	-2.943734	-3.759965
H	0.345615	-3.737878	-4.351884
H	1.590097	-3.412712	-3.142507
H	1.319508	-2.271423	-4.456895
C	1.162100	-2.849772	2.962461
H	0.134299	-2.689475	2.620739
H	1.174593	-3.789124	3.534252
H	1.786002	-2.999890	2.075203
C	-3.408663	-0.448217	3.200498
C	3.113738	-2.059362	4.266041
H	3.773491	-2.193072	3.402557
H	3.099919	-3.010279	4.818333
H	3.561575	-1.307193	4.920718
C	-3.220913	2.540735	1.898294
C	3.287603	1.422974	4.685936
H	3.117114	0.737511	5.522388
H	3.534660	2.404444	5.116061
H	4.168526	1.076980	4.133181
C	-1.129342	-4.840281	-1.967549
H	-1.987023	-4.889915	-2.643505
H	-1.306432	-5.565848	-1.159975
H	-0.243513	-5.175315	-2.517035
C	0.260061	-3.508517	-0.398482
H	1.188858	-3.795838	-0.905755
H	0.071067	-4.251326	0.390405

H	0.431458	-2.543358	0.094472
C	-3.952427	0.097628	0.209330
H	-3.532249	0.439904	-0.740225
H	-4.956152	0.529930	0.304168
H	-4.061144	-0.989210	0.156956
C	-2.723474	-2.909553	-4.401354
H	-2.061038	-3.676958	-4.817763
H	-3.435408	-2.627939	-5.191211
H	-3.302286	-3.361577	-3.589340
C	0.799625	-1.598651	5.092672
H	1.192211	-0.869660	5.808903
H	0.766353	-2.572021	5.603225
H	-0.232264	-1.318604	4.854721
C	3.100515	-0.071997	-4.677060
H	3.157384	0.771318	-5.372484
H	2.058425	-0.408460	-4.655894
H	3.706551	-0.887684	-5.098262
C	0.845689	1.945927	4.663659
H	-0.056492	2.080545	4.061219
H	1.046476	2.899842	5.172881
H	0.626464	1.200099	5.433633
C	3.762962	-0.966571	-2.433301
H	4.492821	-1.646499	-2.897468
H	2.817458	-1.515258	-2.341420
H	4.118028	-0.741179	-1.420029
C	-2.956032	-0.680024	-3.311389
H	-3.449641	-1.101417	-2.430480
H	-3.739721	-0.425550	-4.039990
H	-2.468645	0.254580	-3.010469
C	3.607378	0.309984	-3.279719
C	3.334499	2.325745	-0.990980
H	2.694582	2.741851	-0.207151
H	3.898881	3.156073	-1.433105
H	4.061435	1.659520	-0.518022
C	-1.318552	-0.996058	-5.162651
H	-0.709354	-0.127210	-4.887244
H	-2.105406	-0.642184	-5.844320
H	-0.682862	-1.690696	-5.721558
C	-2.179213	-3.059196	-0.557080
H	-2.063472	-2.065739	-0.106138
H	-2.341552	-3.779125	0.259051
H	-3.083727	-3.049682	-1.175694
C	-2.867482	0.134069	4.512882
H	-1.785827	0.308192	4.466769
H	-3.055336	-0.562020	5.343625
H	-3.345005	1.084654	4.769331
C	-4.939787	-0.584654	3.289198
H	-5.442274	0.371313	3.453920
H	-5.198360	-1.241730	4.132621
H	-5.358699	-1.036230	2.383581
C	1.066539	3.993742	-2.507832
H	0.276006	3.609438	-1.857311
H	0.631438	4.802870	-3.112614
H	1.842034	4.439134	-1.876127
C	-2.834036	-1.859333	3.015657
H	-3.196706	-2.332903	2.097361
H	-3.125852	-2.500318	3.860388
H	-1.739209	-1.849249	2.975938
C	2.294087	2.671276	2.766890
H	3.204470	2.493813	2.184685
H	2.410361	3.634392	3.283831
H	1.457094	2.773530	2.062504
C	2.754712	3.568332	-4.274763
H	3.592589	3.905494	-3.653702
H	2.353879	4.455318	-4.787861
H	3.150282	2.899789	-5.045334
C	-2.169991	3.230059	2.776702
H	-2.147672	2.823154	3.792377
H	-2.393694	4.303795	2.858146
H	-1.167796	3.136255	2.342496
C	0.527794	2.446010	-4.390002
H	0.878147	1.684577	-5.094814
H	0.152876	3.294172	-4.981890
H	-0.324416	2.034953	-3.837051
C	4.996720	0.955473	-3.412357
H	5.434195	1.183009	-2.434595
H	4.972283	1.882490	-3.992908
H	5.681307	0.263501	-3.925186
C	-4.608036	2.768922	2.525018
H	-5.409137	2.285126	1.954548
H	-4.824596	3.847010	2.538799
H	-4.661375	2.415991	3.559044
C	-3.214117	3.233123	0.523960
H	-2.235814	3.180723	0.040391
H	-3.457168	4.297271	0.656885
H	-3.956518	2.810382	-0.160668

Calculated energies and coordinates of **TS3**

Electronic energy ... -3178.11319523 Eh
Total Enthalpy ... -3176.91173905 Eh
Final Gibbs free energy ... -3177.06455236 Eh

CARTESIAN COORDINATES (ANGSTROEM)

Si	2.158207	0.165732	2.873034
Si	-1.130869	-1.925412	-2.771215
Si	-2.555796	0.859649	1.440430
Al	0.233042	0.227840	1.203463
Al	-0.163213	0.090180	-1.589797
Si	1.916906	1.280712	-2.346888
O	-1.259554	1.557837	-1.069760
C	-0.788536	1.329069	0.112507
C	2.165211	1.646206	4.114851
C	-1.376178	-3.512553	-1.669795
C	2.176959	-1.618133	3.625335
C	1.627249	2.311439	-3.968111
C	3.789720	0.313325	1.914547
H	4.042845	-0.604327	1.378709
H	4.594728	0.503052	2.635107
H	3.796734	1.133770	1.194960
C	-2.626218	-1.539820	-3.953723
C	0.319488	-2.361886	-3.930146
H	0.024342	-3.117545	-4.667752
H	1.168482	-2.767267	-3.369367
H	0.677606	-1.483772	-4.475782
C	1.876234	-2.619791	2.496950
H	0.848482	-2.529249	2.128134
H	2.000049	-3.648520	2.863968
H	2.547984	-2.493900	1.638394
C	-2.929716	-0.416447	2.893202
C	3.576767	-1.943746	4.181392
H	4.344182	-1.920929	3.401463
H	3.570299	-2.957893	4.605264
H	3.882871	-1.258657	4.977359
C	-2.944077	2.706829	1.920030
C	3.299906	1.495366	5.143430
H	3.115814	0.664116	5.831882
H	3.363272	2.410760	5.749126
H	4.279926	1.346307	4.677973
C	-1.140885	-4.798161	-2.483208
H	-1.820880	-4.887107	-3.336012
H	-1.303024	-5.675295	-1.839384
H	-0.116197	-4.856891	-2.863428
C	-0.340175	-3.478572	-0.534847
H	0.686819	-3.399959	-0.914355
H	-0.400593	-4.399100	0.065231
H	-0.513166	-2.626854	0.132573
C	-3.771845	0.390744	0.084564
H	-3.569051	0.950968	-0.827968
H	-4.794763	0.617746	0.405068
H	-3.727949	-0.674998	-0.154642
C	-3.070322	-2.793099	-4.726169
H	-2.242942	-3.264327	-5.268145
H	-3.832225	-2.515937	-5.469712
H	-3.518856	-3.544456	-4.068434
C	1.146046	-1.800497	4.745675
H	1.387399	-1.190187	5.622360
H	1.128250	-2.850219	5.072105
H	0.132624	-1.541540	4.421557

C	3.754624	-0.489071	-3.795872
H	4.029960	0.269050	-4.535376
H	2.891525	-1.042795	-4.178117
H	4.593921	-1.197514	-3.727967
C	0.845256	1.801347	4.872973
H	0.014061	1.961450	4.184357
H	0.893956	2.677324	5.535915
H	0.613796	0.930339	5.493743
C	3.245744	-1.036436	-1.431652
H	4.165315	-1.625274	-1.290974
H	2.468710	-1.714903	-1.801793
H	2.922085	-0.680909	-0.447261
C	-3.837318	-0.957286	-3.215964
H	-4.229480	-1.628075	-2.446144
H	-4.650653	-0.762585	-3.930634
H	-3.584203	-0.007010	-2.737378
C	3.484893	0.122382	-2.413937
C	2.238109	2.583209	-0.992873
H	2.497224	2.116080	-0.038181
H	1.334147	3.173786	-0.813855
H	3.052115	3.266437	-1.262539
C	-2.161837	-0.485606	-4.971976
H	-1.841537	0.438610	-4.474460
H	-2.993772	-0.221438	-5.641164
H	-1.337066	-0.843100	-5.597530
C	-2.775400	-3.576779	-1.044764
H	-3.025829	-2.651008	-0.516203
H	-2.827517	-4.398719	-0.315394
H	-3.552950	-3.754832	-1.794657
C	-2.401915	0.052176	4.252117
H	-1.325842	0.244014	4.222851
H	-2.577742	-0.723439	5.012137
H	-2.895798	0.965552	4.597362
C	-4.453224	-0.635200	3.000060
H	-5.007526	0.288365	3.183067
H	-4.656422	-1.312933	3.842143
H	-4.860199	-1.102975	2.098696
C	0.479961	3.300568	-3.693228
H	-0.437747	2.790576	-3.376411
H	0.249964	3.867116	-4.607338
H	0.742004	4.024079	-2.914525
C	-2.307521	-1.781461	2.573308
H	-2.629410	-2.167303	1.600925
H	-2.598959	-2.516385	3.338018
H	-1.211742	-1.738753	2.560652
C	2.408643	2.922856	3.289595
H	3.398453	2.926678	2.821312
H	2.347000	3.804222	3.943217
H	1.661367	3.046721	2.495095
C	2.878728	3.118467	-4.351432
H	3.199541	3.786810	-3.544766
H	2.664021	3.746100	-5.229364
H	3.723882	2.473261	-4.613455
C	-1.870767	3.313026	2.832174
H	-1.855765	2.841124	3.818742
H	-2.071981	4.382993	2.987324
H	-0.871859	3.229703	2.387377
C	1.219023	1.430559	-5.156204
H	2.008724	0.734778	-5.452728
H	0.986956	2.058572	-6.029132
H	0.322813	0.844996	-4.928948
C	4.743413	0.892115	-1.977386
H	4.665285	1.263385	-0.951131
H	4.941613	1.751537	-2.627335
H	5.621729	0.231231	-2.023713
C	-4.307387	2.827837	2.623708
H	-5.125129	2.421596	2.017800
H	-4.531090	3.889938	2.800419
H	-4.320683	2.329655	3.597897
C	-3.004956	3.560196	0.639200
H	-2.041913	3.600804	0.124022
H	-3.282214	4.588839	0.910752
H	-3.752753	3.193921	-0.071639

Calculated energies and coordinates of **INT3**

Electronic energy ... -3178.13648323 Eh
Total Enthalpy ... -3176.93407990 Eh
Final Gibbs free energy ... -3177.08732931 Eh

CARTESIAN COORDINATES (ANGSTROEM)

Si	2.485058	0.179860	2.715337
Si	-1.192774	-1.982661	-2.844156
Si	-2.730134	0.945355	1.189521
Al	0.742047	0.627118	0.897489
Al	-0.427442	-0.017127	-1.455925
Si	1.822880	1.186330	-1.893049
O	-1.220243	1.545122	-0.990546
C	-1.046955	0.989401	0.308719
C	2.431319	1.663348	3.970119
C	-1.403853	-3.567984	-1.733168
C	2.275794	-1.586681	3.483909
C	1.451457	2.326712	-3.438113
C	4.250440	0.217151	2.010328
H	4.509275	-0.690360	1.459308
H	4.950781	0.300129	2.850305
H	4.436677	1.069353	1.353064
C	-2.695957	-1.636163	-4.028142
C	0.268805	-2.391953	-3.992790
H	-0.010684	-3.172043	-4.710646
H	1.129380	-2.763385	-3.425927
H	0.595517	-1.517460	-4.561998
C	1.997764	-2.581431	2.344065
H	1.054573	-2.368880	1.828509
H	1.929989	-3.602776	2.745385
H	2.796207	-2.576909	1.592475
C	-2.823061	-0.289195	2.674746
C	3.575817	-2.028126	4.182973
H	4.414368	-2.101688	3.483392
H	3.427930	-3.025173	4.622003
H	3.869165	-1.354650	4.993676
C	-3.122633	2.787053	1.653388
C	3.358310	1.404520	5.170138
H	3.001533	0.578375	5.793610
H	3.389213	2.300960	5.806191
H	4.388661	1.186184	4.868808
C	-1.152697	-4.858052	-2.533835
H	-1.838549	-4.969604	-3.378944
H	-1.292518	-5.730410	-1.878521
H	-0.130200	-4.902481	-2.922524
C	-0.357545	-3.503557	-0.608484
H	0.664962	-3.428454	-0.999661
H	-0.405314	-4.411375	0.011556
H	-0.529471	-2.642383	0.045873
C	-4.047006	0.420619	-0.049200
H	-3.905608	0.967386	-0.983973
H	-5.056092	0.632503	0.321031
H	-3.994886	-0.649314	-0.273008
C	-3.102867	-2.910283	-4.787914
H	-2.264178	-3.360108	-5.331021
H	-3.876139	-2.662021	-5.529568
H	-3.525747	-3.669808	-4.122798
C	1.126865	-1.651022	4.497967
H	1.330189	-1.040392	5.383877
H	0.987236	-2.686975	4.838784
H	0.177327	-1.317575	4.070168
C	3.538351	-0.496177	-3.562143
H	3.758683	0.280991	-4.299541
H	2.641885	-1.030691	-3.885681
H	4.373360	-1.211698	-3.588629
C	1.020718	1.947177	4.494908
H	0.331346	2.174610	3.674519
H	1.036234	2.823073	5.159678
H	0.611013	1.109316	5.066401
C	3.260720	-1.131467	-1.187646
H	4.176319	-1.742130	-1.195059
H	2.420859	-1.773512	-1.478197
H	3.080182	-0.818192	-0.154015
C	-3.921224	-1.085836	-3.289755

H	-4.302998	-1.771407	-2.527941
H	-4.735998	-0.900639	-4.005209
H	-3.688723	-0.135869	-2.801014
C	3.398137	0.065980	-2.139452
C	2.249871	2.480976	-0.529204
H	2.801746	2.091752	0.333311
H	1.351945	3.003625	-0.172843
H	2.903733	3.249311	-0.964381
C	-2.273394	-0.576096	-5.058549
H	-2.035851	0.378259	-4.574787
H	-3.103610	-0.386834	-5.754611
H	-1.408302	-0.885721	-5.654776
C	-2.798071	-3.644603	-1.097384
H	-3.068737	-2.711205	-0.591943
H	-2.829262	-4.448630	-0.347402
H	-3.573987	-3.858629	-1.839456
C	-2.227402	0.266860	3.972061
H	-1.194091	0.595049	3.833844
H	-2.227319	-0.508933	4.752725
H	-2.796915	1.118018	4.357851
C	-4.286710	-0.688258	2.939814
H	-4.914674	0.174099	3.185118
H	-4.331365	-1.382229	3.791940
H	-4.733562	-1.194867	2.078452
C	0.401421	3.385403	-3.062247
H	-0.537373	2.935575	-2.729935
H	0.194521	4.013347	-3.941079
H	0.750576	4.048532	-2.263420
C	-2.052923	-1.560257	2.292758
H	-2.447761	-2.019599	1.381396
H	-2.112635	-2.306672	3.099194
H	-0.990923	-1.351401	2.112494
C	2.919614	2.927832	3.241164
H	3.946615	2.830552	2.873744
H	2.897316	3.781020	3.933731
H	2.273247	3.185527	2.393233
C	2.726333	3.060626	-3.889577
H	3.170774	3.662664	-3.089150
H	2.472009	3.752347	-4.705971
H	3.493649	2.380295	-4.270049
C	-1.980001	3.450995	2.433708
H	-1.816804	2.985000	3.410493
H	-2.215039	4.510533	2.612131
H	-1.039649	3.405502	1.871919
C	0.907335	1.513039	-4.618781
H	1.631620	0.787589	-4.999433
H	0.643063	2.184078	-5.448908
H	0.000964	0.966695	-4.340109
C	4.688554	0.835041	-1.807057
H	4.706026	1.198220	-0.776334
H	4.830248	1.697350	-2.466784
H	5.558744	0.174768	-1.935701
C	-4.416494	2.886623	2.479240
H	-5.269306	2.430347	1.963466
H	-4.666986	3.944442	2.647323
H	-4.323346	2.416096	3.463382
C	-3.329521	3.582878	0.350795
H	-2.449604	3.541023	-0.298378
H	-3.527899	4.636389	0.597290
H	-4.187151	3.213155	-0.221160

Calculated energies and coordinates of **TS4**

Electronic energy ... -3178.12285075 Eh
Total Enthalpy ... -3176.92119311 Eh
Final Gibbs free energy ... -3177.07240920 Eh

CARTESIAN COORDINATES (ANGSTROEM)

Si	2.553357	0.379151	2.706153
Si	-1.215078	-1.811305	-2.990282
Si	-2.805334	0.590927	1.226942
Al	0.756143	0.574274	0.895725
Al	-0.431770	-0.035051	-1.369957
Si	1.948183	1.047640	-1.749248
O	-0.763356	1.547834	-0.504394
C	-1.084701	0.412207	0.505114
C	2.424720	1.858890	3.955082
C	-1.464947	-3.397274	-1.886721
C	2.325825	-1.393707	3.470578
C	1.474489	2.166382	-3.300061
C	4.349815	0.418623	2.084005
H	4.615814	-0.461282	1.492415
H	5.011546	0.431006	2.958707
H	4.590382	1.304101	1.491687
C	-2.721769	-1.363884	-4.132753
C	0.207461	-2.287509	-4.157531
H	-0.135157	-3.043920	-4.873980
H	1.043825	-2.722500	-3.600190
H	0.589236	-1.438838	-4.728503
C	2.055009	-2.391854	2.331945
H	1.125814	-2.165110	1.792285
H	1.948873	-3.407349	2.739955
H	2.871132	-2.415698	1.601458
C	-2.902444	-0.492558	2.819923
C	3.611594	-1.840376	4.191189
H	4.460593	-1.924809	3.505705
H	3.449544	-2.832679	4.636098
H	3.895991	-1.161603	5.001205
C	-3.140809	2.484440	1.494839
C	3.309527	1.616318	5.189781
H	2.949855	0.778225	5.795425
H	3.295488	2.509816	5.830689
H	4.355121	1.423927	4.925311
C	-1.369201	-4.694169	-2.708574
H	-2.133483	-4.757656	-3.487950
H	-1.506410	-5.558699	-2.042576
H	-0.390130	-4.802974	-3.186317
C	-0.339478	-3.425829	-0.837567
H	0.655890	-3.436872	-1.298325
H	-0.422445	-4.332432	-0.219907
H	-0.396684	-2.563670	-0.160810
C	-4.234169	0.026136	0.125915
H	-4.147583	0.429179	-0.884371
H	-5.183695	0.386302	0.539910
H	-4.307831	-1.062071	0.048948
C	-3.285114	-2.623830	-4.811744
H	-2.519101	-3.175752	-5.368242
H	-4.065021	-2.333065	-5.530483
H	-3.745936	-3.308375	-4.093117
C	1.159308	-1.458213	4.462423
H	1.331390	-0.829579	5.342071
H	1.028666	-2.490843	4.816910
H	0.216223	-1.149608	4.005060
C	3.596449	-0.623748	-3.498770
H	3.808828	0.159861	-4.232291
H	2.681951	-1.138606	-3.804304
H	4.419044	-1.351452	-3.557638
C	0.986984	2.112609	4.419142
H	0.323669	2.300466	3.568656
H	0.950838	3.001091	5.066021
H	0.576387	1.274050	4.988381
C	3.397440	-1.276254	-1.116807
H	4.310523	-1.889140	-1.156511
H	2.545058	-1.914387	-1.377877
H	3.252325	-0.956094	-0.080512
C	-3.846813	-0.674353	-3.354171
H	-4.233656	-1.293493	-2.540300
H	-4.686545	-0.442201	-4.025513
H	-3.499889	0.271937	-2.922740
C	3.506246	-0.075804	-2.067306
C	2.458786	2.407201	-0.486058
H	3.042538	2.044689	0.360234
H	1.582782	2.949306	-0.103976
H	3.083319	3.152153	-0.994783
C	-2.258458	-0.396188	-5.234685
H	-1.924020	0.560316	-4.819274
H	-3.100372	-0.179531	-5.908117
H	-1.446846	-0.808655	-5.843500
C	-2.804512	-3.362476	-1.145118

H	-2.907366	-2.434610	-0.574611
H	-2.869321	-4.200916	-0.435909
H	-3.658678	-3.441272	-1.825812
C	-2.178684	0.173169	3.995457
H	-1.165310	0.476037	3.718739
H	-2.102380	-0.521077	4.846235
H	-2.706092	1.065270	4.348239
C	-4.354452	-0.783732	3.232181
H	-4.913955	0.130323	3.455494
H	-4.365525	-1.406508	4.139003
H	-4.897957	-1.328820	2.453498
C	0.387278	3.180324	-2.908685
H	-0.544715	2.693850	-2.603763
H	0.166216	3.821824	-3.774000
H	0.698529	3.832248	-2.086515
C	-2.218093	-1.834588	2.508359
H	-2.740230	-2.382022	1.715820
H	-2.207258	-2.476004	3.402536
H	-1.181152	-1.696855	2.181740
C	2.921179	3.130713	3.244134
H	3.969350	3.055182	2.936321
H	2.839898	3.987522	3.927652
H	2.321187	3.365960	2.356850
C	2.711332	2.951535	-3.774244
H	3.116702	3.612774	-3.002285
H	2.423188	3.590399	-4.621952
H	3.516703	2.297950	-4.122656
C	-1.932031	3.213347	2.095209
H	-1.701400	2.853737	3.102402
H	-2.140784	4.290786	2.171760
H	-1.043557	3.092665	1.464228
C	0.951757	1.347547	-4.484168
H	1.715023	0.690389	-4.908430
H	0.611092	2.019795	-5.285008
H	0.099756	0.727486	-4.193557
C	4.809371	0.684921	-1.766033
H	4.869788	1.005198	-0.722730
H	4.922316	1.572865	-2.397148
H	5.672711	0.031138	-1.958311
C	-4.353243	2.708637	2.416087
H	-5.252569	2.203272	2.045825
H	-4.582866	3.783176	2.467893
H	-4.169308	2.366636	3.439515
C	-3.465498	3.127932	0.132413
H	-2.654108	2.975638	-0.585381
H	-3.607508	4.210814	0.264930
H	-4.391446	2.729658	-0.296166

Calculated energies and coordinates of **INT4**

Electronic energy ... -3178.12800312 Eh
Total Enthalpy ... -3176.92565171 Eh
Final Gibbs free energy ... -3177.07871453 Eh

CARTESIAN COORDINATES (ANGSTROEM)

Si	2.450170	0.538533	2.837305
Si	-1.228438	-1.710933	-2.989831
Si	-2.816547	0.441402	1.265163
Al	0.575303	0.737410	1.141623
Al	-0.302445	-0.031707	-1.304932
Si	2.030267	0.947562	-1.934451
O	-0.488843	1.498717	-0.188744
C	-1.101791	0.104013	0.554599
C	2.329565	1.972177	4.127671
C	-1.494180	-3.316388	-1.908018
C	2.300820	-1.274019	3.520539
C	1.568806	2.076874	-3.472300
C	4.200787	0.647668	2.112289
H	4.412944	-0.159280	1.405197
H	4.915039	0.549033	2.938519
H	4.413480	1.596217	1.614811
C	-2.738086	-1.231803	-4.118555
C	0.168553	-2.235663	-4.171978
H	-0.198154	-2.993970	-4.874380
H	0.995887	-2.685455	-3.612183
H	0.576017	-1.411884	-4.759700
C	2.019394	-2.217637	2.338177
H	1.068452	-1.988552	1.840045
H	1.955436	-3.255954	2.693903
H	2.811937	-2.180270	1.583246
C	-2.947421	-0.597265	2.888529
C	3.626815	-1.712740	4.169726
H	4.450973	-1.732671	3.450217
H	3.513300	-2.731982	4.565543
H	3.920069	-1.068736	5.005275
C	-3.077926	2.351663	1.493556
C	3.319436	1.747858	5.283544
H	3.042652	0.882621	5.895193
H	3.317681	2.626841	5.944016
H	4.348096	1.605717	4.934694
C	-1.464001	-4.603252	-2.749953
H	-2.254549	-4.631408	-3.504573
H	-1.607155	-5.472692	-2.091089
H	-0.504604	-4.735684	-3.260163
C	-0.340083	-3.398732	-0.892029
H	0.641386	-3.425016	-1.380407
H	-0.430008	-4.318839	-0.295200
H	-0.357213	-2.555357	-0.189835
C	-4.286648	-0.093339	0.205783
H	-4.212025	0.286762	-0.813567
H	-5.213055	0.301740	0.638585
H	-4.398630	-1.179330	0.149799
C	-3.326575	-2.466803	-4.820010
H	-2.570788	-3.024576	-5.384411
H	-4.101196	-2.150412	-5.534082
H	-3.799906	-3.153666	-4.111465
C	1.176432	-1.432836	4.549487
H	1.358555	-0.843561	5.454190
H	1.100486	-2.486408	4.854477
H	0.205291	-1.143076	4.141036
C	3.690531	-0.752284	-3.654115
H	3.916252	0.032028	-4.383801
H	2.777141	-1.262250	-3.972933
H	4.510630	-1.484051	-3.701792
C	0.921660	2.135824	4.710041
H	0.194981	2.375995	3.927973
H	0.909834	2.966866	5.429670
H	0.572695	1.240588	5.232514
C	3.450690	-1.381634	-1.261504
H	4.358604	-2.003471	-1.277800
H	2.597059	-2.017736	-1.522205
H	3.294758	-1.042368	-0.231416
C	-3.849094	-0.542326	-3.320888
H	-4.252299	-1.183854	-2.532751
H	-4.681403	-0.265879	-3.985088
H	-3.480191	0.378629	-2.853559
C	3.581933	-0.195766	-2.228105
C	2.578004	2.271686	-0.665276
H	2.974347	1.835515	0.252181
H	1.738603	2.922628	-0.395543
H	3.372134	2.908274	-1.072627
C	-2.264830	-0.247374	-5.200841
H	-1.899169	0.688993	-4.764774
H	-3.108523	0.008528	-5.858388
H	-1.471379	-0.664811	-5.830004
C	-2.806876	-3.255719	-1.123818
H	-2.852299	-2.343016	-0.523541
H	-2.883847	-4.114326	-0.439671
H	-3.683954	-3.279664	-1.779705
C	-2.221561	0.090603	4.049847
H	-1.208068	0.394505	3.767415
H	-2.136758	-0.589079	4.911223
H	-2.753681	0.985022	4.389991
C	-4.406694	-0.850511	3.300582
H	-4.955895	0.078424	3.485372
H	-4.432475	-1.438984	4.229568
H	-4.952103	-1.417401	2.539373
C	0.428457	3.025535	-3.062150
H	-0.481343	2.481716	-2.775719

H	0.165709	3.674920	-3.910058
H	0.699177	3.671894	-2.221834
C	-2.286553	-1.961137	2.619711
H	-2.804118	-2.513066	1.826806
H	-2.313275	-2.582790	3.527277
H	-1.239252	-1.854189	2.316167
C	2.699096	3.278135	3.400785
H	3.733058	3.275034	3.041885
H	2.588798	4.128848	4.087631
H	2.042822	3.465000	2.540398
C	2.774783	2.927535	-3.905009
H	3.138514	3.578837	-3.104370
H	2.481805	3.577335	-4.743305
H	3.610712	2.310650	-4.251403
C	-1.900929	3.062629	2.173214
H	-1.733341	2.690920	3.187263
H	-2.106948	4.140493	2.252378
H	-0.977637	2.962617	1.589461
C	1.086119	1.274454	-4.683075
H	1.865978	0.630734	-5.098182
H	0.760439	1.956815	-5.482458
H	0.231547	0.647477	-4.421071
C	4.887952	0.553256	-1.909024
H	4.932868	0.881776	-0.866123
H	5.020203	1.436440	-2.543435
H	5.747806	-0.110411	-2.083266
C	-4.337344	2.622683	2.338263
H	-5.228462	2.140240	1.922916
H	-4.535254	3.704311	2.362449
H	-4.224371	2.290515	3.375426
C	-3.301660	2.984405	0.105524
H	-2.454035	2.802863	-0.561865
H	-3.421712	4.072540	0.212702
H	-4.211439	2.602747	-0.369536

Calculated energies and coordinates of **TS5**

Electronic energy ... -3178.11622153 Eh
Total Enthalpy ... -3176.91671719 Eh
Final Gibbs free energy ... -3177.06952229 Eh

CARTESIAN COORDINATES (ANGSTROEM)

Si	2.368863	0.174702	3.119019
Si	-1.092065	-1.877154	-2.826766
Si	-2.741472	0.641357	1.226024
Al	0.452882	0.356961	1.539777
Al	-0.315910	0.124686	-1.387998
Si	1.619426	1.487368	-2.443910
O	0.013519	1.336219	0.126545
C	-1.102022	0.039270	0.483994
C	2.294479	1.523600	4.494449
C	-1.319887	-3.438515	-1.680162
C	2.353458	-1.673324	3.676964
C	1.450853	2.286501	-4.205953
C	3.972152	0.462321	2.150882
H	4.210908	-0.383340	1.500542
H	4.809156	0.593151	2.847582
H	3.918612	1.355729	1.522162
C	-2.622144	-1.545061	-3.994727
C	0.322341	-2.374870	-4.005827
H	-0.009891	-3.139688	-4.718801
H	1.170580	-2.792518	-3.451753
H	0.692354	-1.523074	-4.582621
C	2.025807	-2.532304	2.442193
H	0.995981	-2.372724	2.094534
H	2.114126	-3.599753	2.688995
H	2.700592	-2.335320	1.599521
C	-3.072538	-0.398778	2.831073
C	3.739947	-2.086117	4.202223
H	4.516462	-1.974087	3.438286
H	3.720006	-3.144393	4.498443
H	4.042782	-1.506028	5.079623
C	-2.713640	2.562950	1.480780
C	3.365560	1.247060	5.563449
H	3.137863	0.347251	6.144561
H	3.406091	2.089971	6.267658
H	4.367006	1.132389	5.133560
C	-1.238163	-4.771090	-2.440983
H	-2.027720	-4.877024	-3.189749
H	-1.338837	-5.609573	-1.734841
H	-0.275699	-4.887018	-2.950676
C	-0.177461	-3.420232	-0.649135
H	0.808556	-3.469314	-1.128476
H	-0.254520	-4.288396	0.023997
H	-0.217797	-2.515606	-0.030592
C	-4.213934	0.283152	0.099415
H	-4.079172	0.715371	-0.894111
H	-5.139567	0.692060	0.518743
H	-4.363355	-0.793411	-0.032663
C	-3.165755	-2.832649	-4.632645
H	-2.386377	-3.397832	-5.156205
H	-3.941042	-2.581269	-5.372310
H	-3.627281	-3.494116	-3.892701
C	1.298952	-1.950078	4.756227
H	1.527768	-1.433814	5.694340
H	1.261279	-3.026635	4.974951
H	0.294338	-1.647424	4.437197
C	3.576473	-0.450839	-3.489114
H	3.923799	0.194024	-4.302578
H	2.734626	-1.041901	-3.859295
H	4.395461	-1.149322	-3.258139
C	0.924474	1.618658	5.177438
H	0.146095	1.911136	4.466429
H	0.953259	2.388040	5.961820
H	0.616521	0.678640	5.645253
C	2.896034	-0.635868	-1.111000
H	3.776100	-1.244250	-0.848040
H	2.098480	-1.333511	-1.406296
H	2.580794	-0.112024	-0.195597
C	-3.762128	-0.844499	-3.247830
H	-4.125112	-1.423337	-2.394292
H	-4.614219	-0.671493	-3.922453
H	-3.436454	0.133092	-2.873838
C	3.214768	0.359413	-2.238344
C	1.926200	2.967637	-1.274101
H	2.195207	2.617625	-0.271310
H	1.018314	3.567069	-1.157342
H	2.731388	3.623696	-1.625122
C	-2.171795	-0.605847	-5.124667
H	-1.801658	0.347667	-4.729372
H	-3.023349	-0.376905	-5.782759
H	-1.384632	-1.047448	-5.744632
C	-2.648203	-3.362812	-0.920226
H	-2.747285	-2.397660	-0.411875
H	-2.702253	-4.151590	-0.154160
H	-3.509610	-3.489628	-1.584831
C	-2.222776	0.071832	4.014708
H	-1.152857	0.103789	3.762430
H	-2.324107	-0.622744	4.862034
H	-2.511281	1.067500	4.367736
C	-4.554053	-0.360476	3.244404
H	-4.901247	0.651220	3.475748
H	-4.703994	-0.974654	4.144267
H	-5.201401	-0.767015	2.461146
C	0.253029	3.251359	-4.137957
H	-0.670022	2.731452	-3.848310
H	0.078680	3.708508	-5.123150
H	0.419374	4.063337	-3.421784
C	-2.709634	-1.865151	2.534716
H	-3.342520	-2.289083	1.748964
H	-2.855853	-2.476174	3.437709
H	-1.665822	-1.977364	2.216318
C	2.594228	2.878122	3.826398
H	3.602009	2.915016	3.400808
H	2.518362	3.682489	4.571213
H	1.879313	3.106513	3.025008
C	2.703653	3.089791	-4.591045
H	2.941001	3.864279	-3.854253
H	2.545492	3.593308	-5.556859

H	3.582665	2.445311	-4.701163
C	-1.519451	3.075917	2.300699
H	-1.511212	2.662582	3.313047
H	-1.580197	4.169678	2.403531
H	-0.562915	2.863733	1.808981
C	1.169477	1.259172	-5.308761
H	2.015310	0.586985	-5.477585
H	0.963393	1.772559	-6.260051
H	0.295682	0.647697	-5.071274
C	4.445048	1.185451	-1.832338
H	4.293656	1.721709	-0.890779
H	4.701291	1.927435	-2.596541
H	5.318081	0.527161	-1.703765
C	-3.998120	3.022403	2.193383
H	-4.904601	2.677139	1.684920
H	-4.034138	4.121389	2.215932
H	-4.036253	2.675134	3.231351
C	-2.669541	3.226086	0.090905
H	-1.769223	2.941132	-0.463715
H	-2.656458	4.319962	0.204313
H	-3.549125	2.972473	-0.510315

Calculated energies and coordinates of **INT5**

Electronic energy ... -3178.15614971 Eh
Total Enthalpy ... -3176.95505417 Eh
Final Gibbs free energy ... -3177.10944047 Eh

CARTESIAN COORDINATES (ANGSTROEM)

Si	1.711904	-1.840645	3.169074
Si	0.027509	-1.863698	-2.633017
Si	-2.210676	1.520374	1.117391
Al	0.355998	-0.684861	1.415421
Al	-0.053111	0.365157	-1.356510
Si	0.585328	2.494945	-2.608124
O	0.794441	0.627448	0.302540
C	-1.017038	0.216765	0.432481
C	3.160679	-0.621423	3.559368
C	0.804125	-3.244586	-1.493693
C	0.545162	-2.356248	4.619336
C	-0.351219	2.729992	-4.295550
C	2.504563	-3.447391	2.559210
H	1.762194	-4.228919	2.373475
H	3.189988	-3.817445	3.331359
H	3.080330	-3.307845	1.643907
C	-1.769818	-2.307737	-3.253303
C	1.118818	-1.956587	-4.195262
H	2.184848	-1.969040	-3.946989
H	0.895979	-2.878157	-4.747389
H	0.960160	-1.121082	-4.880473
C	-0.693823	-3.026449	3.998641
H	-1.272217	-2.328509	3.380816
H	-1.363615	-3.389113	4.791209
H	-0.430991	-3.886267	3.370927
C	-3.705838	0.485886	1.796583
C	1.236332	-3.381684	5.535238
H	1.502522	-4.297243	4.997542
H	0.552993	-3.665163	6.348252
H	2.145413	-2.981564	5.995445
C	-1.449086	2.727484	2.416204
C	3.937993	-1.077554	4.805055
H	3.335878	-0.987389	5.715441
H	4.827534	-0.445202	4.937021
H	4.283328	-2.114646	4.725374
C	1.139946	-4.543498	-2.246502
H	0.252677	-5.029814	-2.658354
H	1.622262	-5.258423	-1.562263
H	1.836384	-4.360891	-3.070959
C	2.133143	-2.681210	-0.966514
H	2.789436	-2.344301	-1.776790
H	2.676616	-3.451133	-0.403200
H	1.992581	-1.815633	-0.300359
C	-2.861710	2.545904	-0.323815
H	-2.026189	2.972619	-0.885271
H	-3.525476	3.361568	-0.018048
H	-3.418115	1.902019	-1.015520
C	-1.951368	-3.791476	-3.604452
H	-1.218903	-4.134540	-4.343894
H	-2.950804	-3.950230	-4.037164
H	-1.876072	-4.438126	-2.724273
C	0.093884	-1.158778	5.463472
H	0.931060	-0.697534	5.998371
H	-0.638990	-1.482354	6.216283
H	-0.382635	-0.385095	4.853874
C	2.924212	0.895859	-3.188452
H	2.510102	0.611876	-4.160326
H	2.577347	0.154792	-2.456079
H	4.019485	0.806980	-3.251444
C	2.681587	0.821191	3.771279
H	2.207302	1.227327	2.869007
H	3.541232	1.467520	3.998393
H	1.973249	0.911343	4.600896
C	3.142018	2.605178	-1.396739
H	4.224093	2.407609	-1.425627
H	2.705192	1.972237	-0.614176
H	3.006362	3.649988	-1.098483
C	-2.809713	-1.918799	-2.197256
H	-2.665456	-2.454980	-1.254043
H	-3.827697	-2.137327	-2.554596
H	-2.757501	-0.847447	-1.969414
C	2.525054	2.319324	-2.778509
C	0.367830	4.169904	-1.727289
H	-0.674367	4.504963	-1.705198
H	0.941658	4.935720	-2.263944
H	0.734912	4.143879	-0.700561
C	-2.056990	-1.487667	-4.521773
H	-1.955494	-0.412077	-4.343866
H	-3.089842	-1.666836	-4.855487
H	-1.390054	-1.755424	-5.348048
C	-0.106940	-3.588612	-0.310447
H	-0.453390	-2.685356	0.222878
H	0.407412	-4.237109	0.413296
H	-1.016659	-4.106192	-0.632689
C	-3.493267	0.006617	3.238094
H	-2.543767	-0.527851	3.357309
H	-4.296898	-0.688446	3.522103
H	-3.509257	0.832564	3.955926
C	-4.994418	1.324769	1.740040
H	-4.923732	2.246336	2.327001
H	-5.832489	0.738333	2.144455
H	-5.252487	1.600599	0.712722
C	-1.857290	2.594065	-4.009861
H	-2.106607	1.626330	-3.558191
H	-2.431690	2.683416	-4.944053
H	-2.214703	3.374634	-3.328307
C	-3.909274	-0.751771	0.908536
H	-4.075030	-0.482066	-0.138965
H	-4.791255	-1.314998	1.248168
H	-3.045079	-1.424586	0.945736
C	4.108855	-0.630893	2.345732
H	4.583099	-1.606136	2.197477
H	4.909343	0.106563	2.497680
H	3.590365	-0.357505	1.416706
C	-0.121886	4.120017	-4.913157
H	-0.461203	4.922230	-4.249860
H	-0.692239	4.206150	-5.850186
H	0.929826	4.302715	-5.152051
C	-0.764594	1.997974	3.573057
H	-1.477210	1.499445	4.233672
H	-0.181245	2.704664	4.181430
H	-0.052793	1.242231	3.214570
C	0.054055	1.663281	-5.318527
H	1.096984	1.775292	-5.634425
H	-0.573185	1.734845	-6.220017
H	-0.067078	0.655204	-4.912770
C	3.129507	3.306235	-3.788307
H	2.857240	4.344284	-3.566131
H	2.817122	3.085385	-4.814249
H	4.228204	3.243633	-3.761018

C	-2.515839	3.672160	2.993970
H	-3.048441	4.218374	2.207739
H	-2.035709	4.419504	3.642504
H	-3.255922	3.140697	3.601419
C	-0.391741	3.575450	1.691213
H	0.390686	2.949062	1.248272
H	0.084421	4.267555	2.401482
H	-0.838958	4.177409	0.895077

Calculated energies and coordinates of **TS6**

Electronic energy ... -3178.15303190 Eh
Total Enthalpy ... -3176.95277662 Eh
Final Gibbs free energy ... -3177.10518336 Eh

CARTESIAN COORDINATES (ANGSTROEM)

Si	1.728984	-1.769779	3.166470
Si	0.009638	-1.855916	-2.628394
Si	-2.288278	1.480186	1.095253
Al	0.498413	-0.547540	1.372934
Al	0.092938	0.351780	-1.279503
Si	0.697580	2.486551	-2.591170
O	1.212017	0.503742	0.178077
C	-1.035083	0.235118	0.438476
C	3.144845	-0.550576	3.676220
C	0.789265	-3.268008	-1.526548
C	0.522632	-2.362877	4.552582
C	-0.320746	2.739641	-4.229445
C	2.580688	-3.333540	2.518377
H	1.863236	-4.129781	2.299294
H	3.272247	-3.710342	3.281939
H	3.157161	-3.144407	1.611418
C	-1.815275	-2.247707	-3.204747
C	1.052960	-1.952705	-4.222147
H	2.124485	-1.985527	-4.002266
H	0.799504	-2.866282	-4.774717
H	0.889012	-1.110003	-4.896748
C	-0.675675	-3.034122	3.857757
H	-1.231195	-2.328252	3.228283
H	-1.377805	-3.427740	4.606592
H	-0.368396	-3.872405	3.221255
C	-3.734466	0.404768	1.838200
C	1.196142	-3.404840	5.462182
H	1.513069	-4.290787	4.902431
H	0.484908	-3.738871	6.231212
H	2.071854	-2.998817	5.978191
C	-1.516844	2.727335	2.359778
C	3.868156	-1.032145	4.944016
H	3.226389	-0.968314	5.829379
H	4.747666	-0.399388	5.132055
H	4.223056	-2.065214	4.853783
C	1.034274	-4.578189	-2.295932
H	0.111095	-5.039695	-2.653216
H	1.534333	-5.305697	-1.638401
H	1.687655	-4.418124	-3.159435
C	2.167205	-2.763873	-1.062069
H	2.810558	-2.497719	-1.908157
H	2.683547	-3.551497	-0.496177
H	2.101932	-1.873583	-0.422698
C	-2.993602	2.470666	-0.343865
H	-2.179021	2.940710	-0.902156
H	-3.697720	3.251232	-0.037770
H	-3.515021	1.804421	-1.041575
C	-2.037996	-3.724763	-3.562382
H	-1.328420	-4.079693	-4.318227
H	-3.049353	-3.856573	-3.975932
H	-1.960521	-4.377933	-2.687367
C	0.013056	-1.202156	5.414953
H	0.819772	-0.741707	5.995483
H	-0.742407	-1.561517	6.128660
H	-0.454109	-0.418716	4.809613
C	2.978340	0.847030	-3.269506
H	2.492863	0.556158	-4.205635
H	2.680687	0.121597	-2.500949
H	4.065285	0.747321	-3.409178
C	2.631864	0.878037	3.906722
H	2.225512	1.314375	2.985049
H	3.461024	1.525567	4.226355
H	1.858280	0.927248	4.679682
C	3.326889	2.584212	-1.520727
H	4.403048	2.377579	-1.618691
H	2.938264	1.965811	-0.703175
H	3.219554	3.636293	-1.236098
C	-2.816877	-1.851905	-2.116765
H	-2.663318	-2.416187	-1.191505
H	-3.849715	-2.034692	-2.451490
H	-2.727119	-0.787585	-1.867444
C	2.622421	2.279541	-2.854373
C	0.527344	4.145443	-1.677112
H	-0.514364	4.474856	-1.602646
H	1.074334	4.919408	-2.230381
H	0.943109	4.105791	-0.669089
C	-2.121216	-1.411586	-4.457784
H	-2.004111	-0.339240	-4.272239
H	-3.163112	-1.576742	-4.770144
H	-1.476324	-1.678969	-5.301456
C	-0.076836	-3.577266	-0.301239
H	-0.338091	-2.666987	0.265922
H	0.441911	-4.259590	0.387278
H	-1.028349	-4.043945	-0.578368
C	-3.472189	-0.016579	3.287825
H	-2.502828	-0.512738	3.404136
H	-4.245369	-0.730156	3.608040
H	-3.507210	0.831673	3.978116
C	-5.043978	1.208321	1.778856
H	-4.989495	2.149043	2.336422
H	-5.855211	0.609948	2.219441
H	-5.331142	1.442342	0.749056
C	-1.813005	2.620796	-3.875167
H	-2.050739	1.665102	-3.393298
H	-2.426325	2.698315	-4.785363
H	-2.133922	3.419408	-3.197066
C	-3.914517	-0.862916	0.990208
H	-4.116611	-0.626584	-0.058324
H	-4.765742	-1.448330	1.368266
H	-3.025318	-1.503674	1.024540
C	4.151043	-0.509504	2.510865
H	4.654243	-1.471103	2.368205
H	4.926219	0.241766	2.718377
H	3.670316	-0.229219	1.564026
C	-0.104936	4.132154	-4.848040
H	-0.386652	4.934043	-4.157983
H	-0.733665	4.235643	-5.745076
H	0.931685	4.298823	-5.154213
C	-0.816182	2.047759	3.537009
H	-1.513471	1.563856	4.223765
H	-0.238628	2.786420	4.111248
H	-0.098259	1.288648	3.200621
C	0.021539	1.675550	-5.277561
H	1.052358	1.769385	-5.635572
H	-0.641392	1.769245	-6.150821
H	-0.103768	0.667239	-4.875160
C	3.165876	3.249415	-3.916535
H	2.911440	4.291695	-3.693152
H	2.793781	3.014634	-4.918963
H	4.263740	3.181311	-3.951490
C	-2.591809	3.681040	2.904709
H	-3.130703	4.195027	2.101418
H	-2.116491	4.454249	3.525589
H	-3.324575	3.163508	3.532684
C	-0.469695	3.550835	1.595010
H	0.314944	2.911930	1.172595
H	0.007760	4.270728	2.275651
H	-0.920752	4.120550	0.778278

Calculated energies and coordinates of **INT6** (**4′**)

Electronic energy ... -3178.26104025 Eh

Total Enthalpy ... -3177.05717686 Eh
Final Gibbs free energy ... -3177.20634666 Eh

CARTESIAN COORDINATES (ANGSTROEM)

Si	1.875238	-1.449498	3.041219
Si	-0.982243	-1.737847	-1.578694
Si	-2.473122	0.573033	0.492166
Al	0.590866	-0.634238	1.026097
Al	0.489205	0.779908	-1.124903
Si	1.357048	2.712031	-2.474895
O	1.712965	0.179815	-0.032232
C	-0.993621	-0.337433	-0.266151
C	3.155759	-0.046156	3.460616
C	-0.571845	-3.561565	-0.969904
C	0.923103	-2.164339	4.571245
C	0.382507	3.105587	-4.102623
C	2.918752	-2.896638	2.367985
H	2.341561	-3.824307	2.308341
H	3.771743	-3.078440	3.033379
H	3.320361	-2.683920	1.372192
C	-2.415119	-1.781800	-2.890817
C	0.576631	-1.354588	-2.644153
H	0.600922	-1.993904	-3.532590
H	1.514217	-1.527230	-2.104591
H	0.630602	-0.333792	-3.066023
C	-0.171955	-3.122985	4.069383
H	-0.901936	-2.629533	3.421845
H	-0.723056	-3.541009	4.924490
H	0.255334	-3.966466	3.514643
C	-3.353532	-0.481624	1.870068
C	1.855314	-2.991535	5.477741
H	2.321085	-3.821313	4.935461
H	1.269418	-3.426226	6.300815
H	2.650241	-2.390166	5.924714
C	-1.893414	2.292582	1.233605
C	3.933873	-0.344688	4.752260
H	3.300721	-0.284702	5.643426
H	4.735998	0.398470	4.872849
H	4.407766	-1.332823	4.733295
C	0.295085	-4.310486	-2.006013
H	-0.176292	-4.378817	-2.989305
H	0.462321	-5.337993	-1.652468
H	1.278794	-3.849893	-2.134625
C	0.271080	-3.473269	0.301657
H	1.183425	-2.875809	0.138633
H	0.623229	-4.467758	0.612729
H	-0.290079	-3.062144	1.149114
C	-3.843075	1.160371	-0.688257
H	-3.440497	1.732366	-1.529066
H	-4.501996	1.835492	-0.129467
H	-4.477021	0.372940	-1.097919
C	-2.153871	-2.821635	-3.996675
H	-1.196778	-2.673011	-4.507198
H	-2.940340	-2.730337	-4.759898
H	-2.189897	-3.848929	-3.623683
C	0.284276	-1.051232	5.415069
H	1.043285	-0.427662	5.899290
H	-0.337166	-1.487797	6.210672
H	-0.357017	-0.391304	4.822230
C	3.462116	0.846226	-3.145661
H	2.913016	0.590697	-4.059193
H	3.163243	0.151611	-2.353562
H	4.530062	0.673267	-3.343758
C	2.475950	1.322890	3.600243
H	2.044270	1.643005	2.646905
H	3.216055	2.081525	3.894142
H	1.682365	1.323839	4.356021
C	3.936618	2.500781	-1.349383
H	4.989031	2.190502	-1.427120
H	3.468639	1.894952	-0.565320
H	3.926184	3.549530	-1.034177
C	-3.792508	-2.103287	-2.292696
H	-3.841987	-3.122121	-1.899611
H	-4.564626	-2.011462	-3.070421
H	-4.059238	-1.422910	-1.485295
C	3.241922	2.303098	-2.709741
C	1.298795	4.305144	-1.434008
H	0.316939	4.784917	-1.480887
H	2.038119	5.022695	-1.810265
H	1.526110	4.116412	-0.379955
C	-2.468052	-0.404643	-3.569001
H	-2.604450	0.399993	-2.845191
H	-3.307924	-0.360980	-4.277371
H	-1.554408	-0.202776	-4.138870
C	-1.796533	-4.446281	-0.680626
H	-2.507174	-3.985500	0.008441
H	-1.464889	-5.392745	-0.229092
H	-2.334363	-4.702609	-1.597868
C	-2.569243	-0.424346	3.185551
H	-1.510331	-0.658362	3.022938
H	-2.953750	-1.168174	3.899133
H	-2.619745	0.551639	3.674450
C	-4.803135	-0.035648	2.121836
H	-4.875965	1.006647	2.444682
H	-5.249057	-0.656433	2.912756
H	-5.422155	-0.153936	1.225998
C	-1.112730	3.168747	-3.746598
H	-1.471000	2.223471	-3.326317
H	-1.708596	3.378540	-4.647004
H	-1.326067	3.960737	-3.019607
C	-3.384079	-1.951559	1.437143
H	-3.953754	-2.102682	0.514918
H	-3.849849	-2.570286	2.218720
H	-2.370207	-2.327658	1.274720
C	4.168542	0.039138	2.302675
H	4.790701	-0.860213	2.241775
H	4.841907	0.892450	2.471350
H	3.674065	0.184446	1.336214
C	0.781491	4.472100	-4.687021
H	0.632082	5.282771	-3.965885
H	0.156818	4.694847	-5.564662
H	1.824631	4.497770	-5.012869
C	-0.568085	2.203357	1.998567
H	-0.582756	1.463437	2.807160
H	-0.314612	3.170874	2.457486
H	0.272761	1.966883	1.331250
C	0.591417	2.028707	-5.175731
H	1.629155	1.992974	-5.523573
H	-0.042329	2.235884	-6.050522
H	0.325789	1.028260	-4.814883
C	3.906055	3.234333	-3.735968
H	3.745060	4.293468	-3.503546
H	3.545973	3.049169	-4.753404
H	4.992454	3.061638	-3.738062
C	-2.945271	2.926679	2.162694
H	-3.922468	3.016340	1.677369
H	-2.624793	3.943850	2.431683
H	-3.080721	2.375913	3.096324
C	-1.690814	3.287624	0.078231
H	-1.025754	2.898312	-0.704077
H	-1.232874	4.212906	0.455442
H	-2.635864	3.555625	-0.403321

Calculated energies and coordinates of **TS7**

Electronic energy ... -3178.22229954 Eh
Total Enthalpy ... -3177.02010416 Eh
Final Gibbs free energy ... -3177.17056367 Eh

CARTESIAN COORDINATES (ANGSTROEM)

Si	1.821824	-1.413507	2.914029
Si	-1.500664	-1.890466	-1.261356
Si	-2.712613	0.453067	0.632771
Al	0.417688	-0.725688	0.918853
Al	0.835983	0.313984	-1.759438
Si	1.538137	2.631613	-2.437551
O	1.554695	-0.267265	-0.291670
C	-1.336238	-0.632159	-0.054999
C	3.110208	0.012801	3.221265

C	-0.669227	-3.619341	-0.989984
C	0.988673	-2.103807	4.526920
C	0.830925	3.132580	-4.176298
C	2.857102	-2.867881	2.239173
H	2.297092	-3.808291	2.236482
H	3.742177	-3.013284	2.870765
H	3.207708	-2.677415	1.220128
C	-2.753864	-1.869005	-2.731184
C	0.727227	-0.941661	-3.305308
H	0.191911	-1.892042	-3.237772
H	1.771529	-1.209897	-3.527326
H	0.341307	-0.430589	-4.193690
C	-0.129766	-3.081148	4.121000
H	-0.901627	-2.610956	3.505648
H	-0.624008	-3.481516	5.018566
H	0.271366	-3.935919	3.563372
C	-3.419487	-0.451902	2.203320
C	1.992055	-2.908744	5.375239
H	2.413633	-3.751685	4.817833
H	1.475959	-3.322546	6.254198
H	2.819569	-2.295983	5.740706
C	-2.027430	2.220195	1.027356
C	3.940900	-0.197867	4.496949
H	3.340418	-0.103909	5.407286
H	4.730371	0.566655	4.547091
H	4.435519	-1.175738	4.510813
C	0.183231	-4.197615	-2.131639
H	-0.346388	-4.253744	-3.084299
H	0.480858	-5.220721	-1.862171
H	1.099323	-3.619005	-2.280406
C	0.216912	-3.582591	0.254879
H	1.098142	-2.946603	0.095392
H	0.604899	-4.587679	0.472433
H	-0.325266	-3.251179	1.148405
C	-4.279053	0.824011	-0.378546
H	-4.118939	1.124187	-1.414643
H	-4.775742	1.662686	0.124768
H	-4.996886	0.000531	-0.369437
C	-2.454577	-2.862448	-3.866806
H	-1.480625	-2.689279	-4.333496
H	-3.215814	-2.727135	-4.648037
H	-2.509466	-3.906268	-3.545934
C	0.408879	-0.983315	5.403949
H	1.200877	-0.357220	5.827539
H	-0.153088	-1.412931	6.246386
H	-0.272003	-0.326499	4.855957
C	3.931153	1.094741	-2.958589
H	3.596527	0.937489	-3.989877
H	3.563875	0.260127	-2.346643
H	5.029437	1.030145	-2.952180
C	2.420659	1.381967	3.302578
H	1.959088	1.647804	2.345721
H	3.158244	2.163927	3.535681
H	1.645309	1.415229	4.077057
C	3.892413	2.503344	-0.908709
H	4.962774	2.269420	-0.808377
H	3.337665	1.773405	-0.308682
H	3.729121	3.496011	-0.476062
C	-4.151422	-2.219507	-2.176982
H	-4.192889	-3.239750	-1.781594
H	-4.887488	-2.149866	-2.990389
H	-4.466641	-1.538810	-1.387665
C	3.476992	2.448293	-2.389432
C	1.159655	4.100994	-1.291568
H	0.162233	4.509022	-1.474039
H	1.882047	4.907145	-1.469314
H	1.218206	3.826626	-0.234325
C	-2.765208	-0.458797	-3.349782
H	-2.754557	0.332329	-2.598601
H	-3.660721	-0.331065	-3.973099
H	-1.890377	-0.314871	-3.988893
C	-1.845712	-4.584438	-0.706806
H	-2.495132	-4.230073	0.102857
H	-1.437852	-5.557089	-0.398380
H	-2.464644	-4.757056	-1.591266
C	-2.448130	-0.335369	3.379140
H	-1.436004	-0.636768	3.087349
H	-2.753497	-0.990305	4.208600
H	-2.390994	0.684825	3.771633
C	-4.786546	0.106604	2.636911
H	-4.753275	1.177166	2.857694
H	-5.115444	-0.406664	3.552101
H	-5.558074	-0.057123	1.878159
C	-0.688618	2.895888	-4.138110
H	-0.929615	1.839166	-3.974707
H	-1.147323	3.199879	-5.090919
H	-1.172815	3.473978	-3.341448
C	-3.604127	-1.943505	1.882030
H	-4.248319	-2.100605	1.006836
H	-4.071995	-2.458483	2.733692
H	-2.642740	-2.426229	1.679460
C	4.079793	0.030321	2.025211
H	4.736819	-0.845982	2.026652
H	4.721045	0.922407	2.082045
H	3.548008	0.054352	1.068739
C	1.069854	4.621445	-4.483445
H	0.606037	5.272491	-3.735236
H	0.627665	4.874473	-5.458649
H	2.134350	4.870377	-4.531189
C	-0.605569	2.161963	1.592466
H	-0.537220	1.551283	2.501049
H	-0.243710	3.166309	1.858091
H	0.104163	1.779288	0.843887
C	1.429773	2.297569	-5.317168
H	2.494279	2.508805	-5.461247
H	0.920094	2.530892	-6.263948
H	1.325521	1.221287	-5.144403
C	4.199511	3.574688	-3.141950
H	3.889783	4.566751	-2.793642
H	4.029320	3.526214	-4.222323
H	5.284909	3.495545	-2.978160
C	-2.908552	3.011964	2.007971
H	-3.946618	3.085441	1.666080
H	-2.525232	4.038789	2.096746
H	-2.912402	2.580823	3.013156
C	-1.989665	3.005313	-0.293989
H	-1.374529	2.501511	-1.050841
H	-1.553089	3.998144	-0.122423
H	-2.991294	3.153170	-0.710134

Calculated energies and coordinates of **INT7**

Electronic energy ... -3178.22962835 Eh
Total Enthalpy ... -3177.02760721 Eh
Final Gibbs free energy ... -3177.18230193 Eh

CARTESIAN COORDINATES (ANGSTROEM)

Si	1.476580	-0.826348	2.914244
Si	-1.701610	-1.781429	-1.230712
Si	-3.314114	0.193717	0.784009
Al	-0.049930	-0.577592	0.904537
Al	2.528417	-0.094949	-1.236569
Si	2.959655	1.940071	-2.587135
O	1.071942	-0.280664	-0.375012
C	-1.856774	-0.782374	0.174646
C	2.516839	0.803528	3.182829
C	-0.600644	-3.372597	-1.141507
C	0.801460	-1.605873	4.563198
C	1.534412	3.229045	-2.380943
C	2.737271	-2.108798	2.277582
H	2.318670	-3.121070	2.286544
H	3.633213	-2.120025	2.910018
H	3.057065	-1.895738	1.254696
C	-2.663707	-1.602645	-2.901539
C	3.777324	-1.622090	-1.338956
H	3.981305	-1.891829	-2.383384
H	3.419424	-2.527058	-0.836321
H	4.749890	-1.364011	-0.897275
C	-0.218989	-2.693006	4.178262

H	-1.081450	-2.287843	3.642635
H	-0.595718	-3.194269	5.082176
H	0.236695	-3.464451	3.544941
C	-3.875861	-0.581804	2.474213
C	1.921079	-2.305314	5.357924
H	2.387291	-3.112062	4.783775
H	1.494391	-2.755072	6.266645
H	2.709746	-1.617121	5.674343
C	-2.841564	2.068560	0.920028
C	3.509074	0.651062	4.347833
H	2.998036	0.566163	5.311462
H	4.155489	1.539638	4.400422
H	4.162283	-0.220775	4.230323
C	0.528809	-3.474923	-2.179243
H	0.161155	-3.524603	-3.204789
H	1.111063	-4.389203	-1.994950
H	1.220169	-2.629413	-2.102098
C	0.029970	-3.508309	0.250334
H	0.831753	-2.773570	0.402495
H	0.512421	-4.492071	0.343322
H	-0.706915	-3.417301	1.055616
C	-4.915743	0.140050	-0.234641
H	-4.818308	0.395561	-1.290457
H	-5.622777	0.854243	0.203678
H	-5.387192	-0.846299	-0.166185
C	-1.941083	-2.250293	-4.095233
H	-0.931749	-1.848018	-4.235466
H	-2.510777	-2.025722	-5.007985
H	-1.874959	-3.338766	-4.018322
C	0.132624	-0.577173	5.487224
H	0.866962	0.109570	5.921469
H	-0.366445	-1.089658	6.322852
H	-0.623377	0.025283	4.976158
C	2.158375	0.171523	-4.706101
H	1.127647	0.532880	-4.624130
H	2.266958	-0.689472	-4.030815
H	2.296970	-0.209104	-5.728913
C	1.626225	2.023619	3.456891
H	0.987959	2.253628	2.598517
H	2.248840	2.909979	3.648756
H	0.979331	1.879812	4.328268
C	4.597156	0.644630	-4.484759
H	4.724174	0.138400	-5.452836
H	4.770900	-0.099839	-3.698326
H	5.381398	1.404512	-4.402968
C	-4.064091	-2.235923	-2.786790
H	-4.009012	-3.316027	-2.613720
H	-4.612701	-2.079374	-3.726982
H	-4.653548	-1.794824	-1.981470
C	3.192781	1.267983	-4.397047
C	4.576184	2.813412	-2.095655
H	4.456066	3.353766	-1.150463
H	4.882253	3.541417	-2.857077
H	5.397795	2.102204	-1.965036
C	-2.782807	-0.101782	-3.224080
H	-3.195777	0.476395	-2.398685
H	-3.430288	0.034971	-4.101795
H	-1.802779	0.327783	-3.461332
C	-1.575385	-4.559480	-1.318963
H	-2.370002	-4.549131	-0.562547
H	-1.021542	-5.501888	-1.202837
H	-2.043586	-4.577167	-2.306883
C	-2.912385	-0.188712	3.596487
H	-1.870131	-0.346801	3.297872
H	-3.088379	-0.796801	4.496359
H	-3.022905	0.862493	3.884425
C	-5.300595	-0.166341	2.878630
H	-5.416364	0.918845	2.961509
H	-5.543716	-0.598464	3.860441
H	-6.049515	-0.531095	2.168693
C	1.298706	3.417704	-0.872501
H	0.989138	2.477993	-0.402234
H	0.497438	4.152600	-0.704470
H	2.193841	3.781426	-0.353681
C	-3.856088	-2.113745	2.330210
H	-4.518670	-2.458113	1.525867
H	-4.196849	-2.583114	3.264910
H	-2.850472	-2.480478	2.103779
C	3.332348	1.093931	1.909290
H	4.024305	0.278514	1.666162
H	3.931691	2.005906	2.046154
H	2.671327	1.270774	1.050853
C	1.885356	4.595668	-2.989927
H	2.791397	5.018024	-2.541038
H	1.066608	5.307192	-2.806999
H	2.038852	4.543628	-4.071803
C	-1.463676	2.254603	1.559922
H	-1.406412	1.861334	2.579147
H	-1.188289	3.319403	1.597182
H	-0.681259	1.774008	0.950257
C	0.233922	2.703601	-3.002598
H	0.305710	2.611163	-4.091573
H	-0.598521	3.388664	-2.784828
H	-0.030930	1.721515	-2.589621
C	3.089557	2.364983	-5.467073
H	3.779623	3.193926	-5.270689
H	2.077890	2.776727	-5.539003
H	3.345485	1.952320	-6.454482
C	-3.865066	2.890527	1.718554
H	-4.879395	2.785242	1.317866
H	-3.605847	3.958484	1.671513
H	-3.888112	2.608768	2.776000
C	-2.767966	2.637537	-0.507309
H	-2.041163	2.088182	-1.115647
H	-2.447750	3.689715	-0.477695
H	-3.739647	2.603119	-1.012936

Calculated energies and coordinates of **TS8**

Electronic energy ... -3178.22736073 Eh
Total Enthalpy ... -3177.02716530 Eh
Final Gibbs free energy ... -3177.18021967 Eh

CARTESIAN COORDINATES (ANGSTROEM)

Si	1.609753	-0.750988	2.778720
Si	-1.502550	-1.861319	-1.040519
Si	-3.362104	0.089630	0.837276
Al	-0.054139	-0.622662	0.901748
Al	2.276815	-0.342729	-1.493571
Si	2.733112	1.845051	-2.605151
O	0.871809	-0.742906	-0.587761
C	-1.872984	-0.859658	0.330273
C	2.733396	0.834530	2.825977
C	-0.711553	-3.602928	-0.749161
C	0.897718	-1.303221	4.491250
C	1.536252	3.301418	-2.158397
C	2.767885	-2.167865	2.233868
H	2.333584	-3.147487	2.458250
H	3.730033	-2.110391	2.757664
H	2.970451	-2.141046	1.159126
C	-2.075298	-1.568902	-2.858429
C	3.618170	-1.761328	-1.767701
H	3.434520	-2.293672	-2.711779
H	3.625376	-2.519485	-0.976922
H	4.630150	-1.343721	-1.839695
C	-0.102239	-2.444194	4.226369
H	-0.902470	-2.150430	3.539812
H	-0.571980	-2.758383	5.169763
H	0.393368	-3.323866	3.799002
C	-4.163583	-0.740005	2.401578
C	1.989039	-1.859979	5.423730
H	2.523973	-2.700741	4.969091
H	1.526260	-2.229061	6.350842
H	2.726313	-1.103969	5.706492
C	-2.910073	1.959731	1.121192
C	3.754840	0.778331	3.972597
H	3.267466	0.871318	4.948160
H	4.464114	1.614515	3.882972
H	4.339122	-0.148735	3.970896

C	0.544967	-3.954353	-1.557119
H	0.382196	-3.939590	-2.635740
H	0.874555	-4.969299	-1.291653
H	1.362678	-3.268216	-1.322566
C	-0.366237	-3.770468	0.735929
H	0.463486	-3.114638	1.034613
H	-0.023588	-4.799032	0.920263
H	-1.218631	-3.565309	1.387953
C	-4.781617	0.113380	-0.424661
H	-4.474016	0.436635	-1.421928
H	-5.565334	0.802041	-0.088514
H	-5.246136	-0.874906	-0.517010
C	-1.184360	-2.268672	-3.896415
H	-0.125121	-2.014298	-3.774961
H	-1.484551	-1.941409	-4.901863
H	-1.282052	-3.357414	-3.865913
C	0.177440	-0.150011	5.203443
H	0.883970	0.613014	5.548012
H	-0.359490	-0.523870	6.087308
H	-0.557175	0.340958	4.556259
C	1.669121	0.655639	-4.992410
H	0.722436	1.188696	-4.857244
H	1.590166	-0.302708	-4.460482
H	1.769692	0.421784	-6.062517
C	1.914817	2.126201	2.962927
H	1.247739	2.273937	2.108520
H	2.586214	2.996765	3.006825
H	1.300672	2.136042	3.869093
C	4.145547	0.622407	-4.726594
H	4.213338	0.322471	-5.782418
H	4.143973	-0.294350	-4.125155
H	5.055334	1.181095	-4.483098
C	-3.525897	-2.044883	-3.061748
H	-3.622476	-3.129851	-2.952867
H	-3.852330	-1.785064	-4.079357
H	-4.217916	-1.571481	-2.362908
C	2.878708	1.467626	-4.504386
C	4.463577	2.377784	-2.012696
H	4.422453	2.775205	-0.992695
H	4.880019	3.161390	-2.657507
H	5.167601	1.539395	-2.008889
C	-1.984908	-0.056290	-3.113854
H	-2.580192	0.521456	-2.404053
H	-2.334790	0.171734	-4.130847
H	-0.948057	0.286163	-3.028272
C	-1.840407	-4.604051	-1.085170
H	-2.762072	-4.389604	-0.528254
H	-1.519781	-5.618002	-0.807494
H	-2.077969	-4.618713	-2.153336
C	-3.366483	-0.421848	3.671572
H	-2.299482	-0.619005	3.529483
H	-3.706154	-1.047117	4.511024
H	-3.478888	0.624000	3.977672
C	-5.626604	-0.316228	2.612210
H	-5.738421	0.765326	2.735927
H	-6.023789	-0.793048	3.520673
H	-6.264032	-0.625836	1.777554
C	1.252734	3.216555	-0.649916
H	0.767764	2.267330	-0.388769
H	0.576711	4.027025	-0.339648
H	2.168744	3.306161	-0.054230
C	-4.139550	-2.262910	2.189144
H	-4.661410	-2.556903	1.269222
H	-4.635792	-2.770461	3.029569
H	-3.112409	-2.631805	2.116556
C	3.508185	0.897800	1.497949
H	4.151655	0.024458	1.345280
H	4.148291	1.791783	1.471448
H	2.818395	0.983608	0.643408
C	2.179703	4.668172	-2.449217
H	3.108467	4.810719	-1.886842
H	1.489656	5.472170	-2.154145
H	2.405327	4.803593	-3.511547
C	-1.630023	2.128786	1.941482
H	-1.703816	1.721916	2.954352
H	-1.352192	3.190505	2.022826
H	-0.775839	1.639567	1.445993
C	0.199804	3.211225	-2.903907
H	0.319173	3.336796	-3.984988
H	-0.480327	4.001972	-2.554759
H	-0.300009	2.253848	-2.726997
C	3.004811	2.742951	-5.351810
H	3.842438	3.370238	-5.025090
H	2.095934	3.351852	-5.324270
H	3.187414	2.475963	-6.403349
C	-4.035002	2.749541	1.808490
H	-4.985280	2.660030	1.270530
H	-3.778311	3.818798	1.840085
H	-4.198112	2.423585	2.840532
C	-2.652386	2.589591	-0.258500
H	-1.860402	2.055639	-0.795309
H	-2.329801	3.635334	-0.144070
H	-3.550491	2.589500	-0.886021

Calculated energies and coordinates of **TS9**

Electronic energy ... -3291.54773260 Eh
Total Enthalpy ... -3290.33700443 Eh
Final Gibbs free energy ... -3290.49435820 Eh

CARTESIAN COORDINATES (ANGSTROEM)

Si	-1.292872	4.112652	-0.438495
Al	-0.241741	2.133429	-1.556775
C	-0.251789	2.285870	-3.519177
H	0.587127	2.871491	-3.912995
H	-1.165709	2.821110	-3.810593
H	-0.258544	1.322284	-4.040503
C	-0.200762	5.690061	-0.780860
C	-2.881724	4.387825	-1.455587
C	-1.813351	3.897208	1.407664
C	-0.631071	3.429810	2.266641
H	-0.247906	2.466289	1.911592
H	-0.949505	3.294642	3.310544
H	0.199847	4.141026	2.266986
C	-1.082521	6.951477	-0.831833
H	-1.633192	7.116716	0.099288
H	-1.808662	6.908101	-1.649700
H	-0.449254	7.834410	-1.001689
C	-2.894223	2.806195	1.455927
H	-3.791518	3.084967	0.891748
H	-3.201771	2.622113	2.496003
H	-2.515336	1.855999	1.058730
C	0.473107	5.538432	-2.156204
H	0.996043	6.468836	-2.421350
H	-0.250524	5.330786	-2.953109
H	1.220114	4.736442	-2.162768
C	-2.396066	5.187896	2.003696
H	-1.643116	5.977747	2.087681
H	-2.775720	4.990734	3.016789
H	-3.232407	5.575717	1.410823
C	0.893317	5.903883	0.275210
H	1.530287	5.022111	0.404737
H	0.472981	6.160492	1.252675
H	1.544000	6.737251	-0.027602
Si	-2.808063	-1.566182	-0.263216
Al	-0.389486	-0.932074	0.071652
Si	1.935527	-0.176999	-0.695694
Si	2.258994	-2.623498	1.541517
O	0.293236	0.570234	-0.890626
O	-1.478632	0.493593	4.394946
C	-2.889665	-3.499345	-0.239857
C	-1.397314	0.342741	3.276484
C	-3.522599	-0.785522	-1.892944
C	-5.061227	-0.794972	-1.876407
H	-5.461895	-0.204155	-1.045901
H	-5.443721	-0.356124	-2.809432
H	-5.469289	-1.806491	-1.797312
C	-3.913401	-0.950736	1.153184
H	-4.068988	0.132222	1.113662

H	-4.898862	-1.430196	1.107992
H	-3.473508	-1.183365	2.127595
C	-2.693660	-3.938943	1.220907
H	-3.542596	-3.646616	1.848260
H	-2.598659	-5.032813	1.275730
H	-1.788810	-3.510462	1.663860
C	1.364163	-1.469740	0.428585
C	-4.247071	-4.040987	-0.716155
H	-4.421392	-3.852354	-1.779959
H	-4.277712	-5.130668	-0.570601
H	-5.083613	-3.614496	-0.150274
C	3.013480	1.333138	-0.139674
C	1.626347	-2.401984	3.377408
C	-3.020765	-1.500728	-3.154586
H	-3.417027	-2.516948	-3.235483
H	-3.341796	-0.951691	-4.051935
H	-1.925989	-1.564576	-3.179862
C	-1.774662	-4.111766	-1.101459
H	-0.783836	-3.754530	-0.796545
H	-1.773990	-5.206737	-1.000775
H	-1.897470	-3.876838	-2.163454
C	2.218549	-4.516617	1.033497
C	-3.067750	0.677632	-1.967928
H	-1.989932	0.709535	-2.182289
H	-3.562728	1.204504	-2.796623
H	-3.276274	1.229521	-1.044565
C	2.433660	-0.753006	-2.478866
C	1.940042	-0.961406	3.813525
H	3.018141	-0.764352	3.828398
H	1.470481	-0.236384	3.140694
H	1.559685	-0.783847	4.831062
C	2.792658	1.610527	1.352206
H	1.729750	1.707862	1.595695
H	3.190924	0.803306	1.969540
H	3.295477	2.544942	1.643737
C	2.588844	2.587928	-0.916541
H	3.248026	3.437429	-0.683640
H	2.597480	2.461841	-2.003874
H	1.588830	2.933735	-0.583948
C	0.920697	-5.215867	1.461626
H	0.886694	-6.233358	1.043245
H	0.843098	-5.311976	2.548806
H	0.032929	-4.687791	1.103139
C	1.253277	-1.548986	-3.061316
H	0.383438	-0.903163	-3.232660
H	1.534971	-1.986108	-4.029920
H	0.948742	-2.365145	-2.397790
C	2.313303	-3.352066	4.371211
H	2.006658	-3.104008	5.398583
H	2.044108	-4.398463	4.198392
H	3.405360	-3.270666	4.329122
C	4.113080	-2.195993	1.586362
C	0.110024	-2.594788	3.492586
H	-0.421401	-1.977445	2.755022
H	-0.193022	-3.633796	3.343088
H	-0.242435	-2.288211	4.489347
C	2.335996	-4.651289	-0.492136
H	1.572360	-4.066701	-1.015020
H	3.318268	-4.323520	-0.848257
H	2.224691	-5.705823	-0.786419
C	3.638025	-1.699610	-2.337735
H	3.432308	-2.493397	-1.618253
H	3.859104	-2.172045	-3.305919
H	4.542311	-1.172959	-2.015184
C	4.517367	1.106494	-0.371696
H	5.086566	1.942354	0.060092
H	4.869261	0.188585	0.108975
H	4.774389	1.050578	-1.432819
C	2.804796	0.337402	-3.495341
H	3.691785	0.905503	-3.200069
H	3.037034	-0.132768	-4.462289
H	1.986281	1.039477	-3.671490
C	3.402725	-5.290407	1.642881
H	3.337039	-6.350963	1.356221
H	4.363446	-4.913269	1.277685
H	3.421065	-5.247914	2.735675
H	4.270248	-1.138332	1.817868
H	4.584170	-2.395100	0.615865
H	4.656678	-2.779497	2.337015
H	-3.509142	3.493908	-1.504067
H	-2.640219	4.671286	-2.486031
H	-3.485922	5.195890	-1.026148

Calculated energies and coordinates of **INT9**

Electronic energy ... -3291.55557136 Eh
Total Enthalpy ... -3290.34385107 Eh
Final Gibbs free energy ... -3290.50108565 Eh

CARTESIAN COORDINATES (ANGSTROEM)

Si	-1.314486	4.193678	-0.430408
Al	-0.285438	2.141473	-1.450306
C	-0.342572	2.213523	-3.416279
H	0.484779	2.782488	-3.855754
H	-1.264918	2.732820	-3.709219
H	-0.357865	1.226902	-3.892338
C	-0.219686	5.741275	-0.881488
C	-2.901592	4.395486	-1.465718
C	-1.840628	4.107977	1.425348
C	-0.653112	3.725855	2.319139
H	-0.175106	2.805551	1.969371
H	-0.987596	3.550838	3.351735
H	0.115358	4.503048	2.344669
C	-1.100823	6.996734	-1.017826
H	-1.653620	7.223274	-0.101004
H	-1.825185	6.901483	-1.833001
H	-0.466190	7.866185	-1.243102
C	-2.914187	3.013128	1.541485
H	-3.830040	3.279042	1.001711
H	-3.180153	2.849964	2.595788
H	-2.554773	2.054236	1.146302
C	0.456528	5.497819	-2.241864
H	0.979459	6.408540	-2.568151
H	-0.265111	5.236275	-3.024378
H	1.204219	4.697785	-2.192535
C	-2.444219	5.428359	1.929581
H	-1.703070	6.233069	1.958869
H	-2.822267	5.296854	2.953842
H	-3.285644	5.761788	1.311458
C	0.872313	6.023192	0.160294
H	1.501595	5.147639	0.353887
H	0.449080	6.350953	1.114799
H	1.530240	6.828895	-0.196942
Si	-2.815368	-1.609712	-0.173681
Al	-0.435217	-0.856088	0.245375
Si	1.903095	-0.167912	-0.597934
Si	2.243103	-2.675385	1.575024
O	0.269464	0.589987	-0.768675
O	-1.069963	0.404997	3.140419
C	-2.863129	-3.540590	-0.249695
C	-0.888643	0.307900	2.017954
C	-3.575835	-0.759845	-1.749565
C	-5.111557	-0.858871	-1.754685
H	-5.553573	-0.373573	-0.877986
H	-5.512402	-0.356459	-2.647339
H	-5.459212	-1.894759	-1.777550
C	-3.905041	-1.064355	1.288376
H	-4.063292	0.020392	1.293110
H	-4.891556	-1.541368	1.242687
H	-3.454868	-1.332594	2.249968
C	-2.681389	-4.048806	1.190385
H	-3.547485	-3.806802	1.815817
H	-2.561373	-5.141676	1.192635
H	-1.793882	-3.622863	1.669676
C	1.334237	-1.482425	0.510454
C	-4.190731	-4.100921	-0.782661
H	-4.347027	-3.865239	-1.839967
H	-4.188494	-5.197042	-0.691784

H	-5.053499	-3.729748	-0.216987
C	2.966755	1.342637	-0.016896
C	1.624182	-2.524546	3.423040
C	-3.020348	-1.354912	-3.050052
H	-3.342536	-2.389867	-3.197304
H	-3.375807	-0.774684	-3.914330
H	-1.923730	-1.340590	-3.069553
C	-1.709338	-4.066706	-1.117875
H	-0.740937	-3.692034	-0.767462
H	-1.672810	-5.165276	-1.082333
H	-1.815086	-3.772585	-2.167435
C	2.208890	-4.553705	1.008391
C	-3.214906	0.729106	-1.717480
H	-2.129051	0.844001	-1.855098
H	-3.687839	1.269229	-2.550284
H	-3.511359	1.216358	-0.782888
C	2.418714	-0.731727	-2.375424
C	1.967782	-1.106001	3.908420
H	3.050936	-0.942153	3.942654
H	1.531681	-0.344221	3.257620
H	1.578932	-0.949725	4.925820
C	2.709737	1.598926	1.473067
H	1.640176	1.700659	1.689280
H	3.088857	0.779790	2.087181
H	3.205689	2.527503	1.793177
C	2.571487	2.608130	-0.790379
H	3.228794	3.450570	-0.527714
H	2.615453	2.492975	-1.877929
H	1.563423	2.961037	-0.491712
C	0.915437	-5.268393	1.424184
H	0.887018	-6.276721	0.984199
H	0.837395	-5.388209	2.508729
H	0.025890	-4.737122	1.076738
C	1.242344	-1.529067	-2.965106
H	0.375181	-0.882668	-3.142641
H	1.530306	-1.967968	-3.931077
H	0.928653	-2.341365	-2.301763
C	2.306887	-3.515248	4.379816
H	2.005377	-3.299963	5.415987
H	2.029777	-4.552929	4.172104
H	3.399307	-3.439801	4.336468
C	4.096130	-2.243403	1.629053
C	0.106115	-2.713026	3.532791
H	-0.424954	-2.156680	2.751291
H	-0.187231	-3.762022	3.443191
H	-0.264033	-2.345162	4.501418
C	2.318427	-4.654926	-0.520714
H	1.558467	-4.053051	-1.028148
H	3.302447	-4.332268	-0.876104
H	2.193560	-5.701595	-0.836628
C	3.629125	-1.669500	-2.237115
H	3.428256	-2.469505	-1.523343
H	3.855060	-2.135792	-3.207137
H	4.529011	-1.138523	-1.909657
C	4.474874	1.110769	-0.215091
H	5.038872	1.933925	0.246879
H	4.808564	0.180606	0.254385
H	4.757314	1.074697	-1.270999
C	2.784451	0.371472	-3.378888
H	3.668093	0.941543	-3.076408
H	3.019242	-0.087084	-4.350827
H	1.961496	1.070588	-3.547559
C	3.399197	-5.338886	1.591133
H	3.336613	-6.390451	1.272351
H	4.356807	-4.946777	1.233794
H	3.421299	-5.330007	2.684428
H	4.252994	-1.196243	1.904665
H	4.566899	-2.403455	0.651342
H	4.640585	-2.856745	2.355174
H	-3.529287	3.500727	-1.445685
H	-2.660389	4.599803	-2.514739
H	-3.506210	5.233908	-1.099772

Calculated energies and coordinates of **TS10**

Electronic energy ... -3291.54349947 Eh
Total Enthalpy ... -3290.33166960 Eh
Final Gibbs free energy ... -3290.48676902 Eh

CARTESIAN COORDINATES (ANGSTROEM)

Si	-1.427974	4.043884	-0.335730
Al	-0.169872	2.218774	-1.511227
C	-0.051173	2.435981	-3.464973
H	0.774744	3.084075	-3.780876
H	-0.973110	2.933095	-3.796187
H	0.030526	1.499488	-4.025016
C	-0.641891	5.756716	-0.828708
C	-3.136914	3.997207	-1.179467
C	-1.691535	3.817030	1.559792
C	-0.356358	3.577279	2.275598
H	0.160927	2.715461	1.847167
H	-0.527603	3.347991	3.336581
H	0.315279	4.438918	2.223716
C	-1.718916	6.856024	-0.800834
H	-2.194527	6.950358	0.180174
H	-2.505538	6.674829	-1.540463
H	-1.259971	7.826244	-1.040567
C	-2.561918	2.565117	1.747169
H	-3.573596	2.705250	1.351994
H	-2.649392	2.322721	2.815572
H	-2.121314	1.686869	1.259298
C	-0.082290	5.680037	-2.257955
H	0.255091	6.675496	-2.581286
H	-0.827842	5.335333	-2.983252
H	0.783661	5.010160	-2.318703
C	-2.413653	5.009713	2.205746
H	-1.803156	5.918424	2.200214
H	-2.639384	4.776744	3.256297
H	-3.365371	5.234742	1.710167
C	0.506079	6.168113	0.104003
H	1.292109	5.405520	0.153073
H	0.160767	6.363416	1.123489
H	0.969504	7.093694	-0.267370
Si	-2.850789	-1.443404	-0.665613
Al	-0.516440	-0.802338	0.017577
Si	1.971811	-0.085470	-0.486034
Si	2.223296	-2.592215	1.678067
O	0.375601	0.646408	-0.862304
O	0.123381	0.281341	2.864084
C	-3.222630	-3.243177	-0.071093
C	-0.037659	-0.138362	1.779358
C	-3.258689	-1.080622	-2.524022
C	-4.771636	-0.921153	-2.750389
H	-5.185865	-0.094689	-2.163420
H	-4.966825	-0.702769	-3.810495
H	-5.329419	-1.826880	-2.493408
C	-4.009805	-0.313573	0.335220
H	-4.039505	0.699216	-0.079042
H	-5.034453	-0.704250	0.320352
H	-3.696933	-0.226225	1.379932
C	-3.072759	-3.240062	1.461656
H	-3.829789	-2.613725	1.944928
H	-3.183609	-4.261356	1.853317
H	-2.086167	-2.876641	1.781350
C	1.283853	-1.248984	0.759613
C	-4.651948	-3.683574	-0.424528
H	-4.781731	-3.808389	-1.504884
H	-4.867662	-4.655281	0.043382
H	-5.408740	-2.975157	-0.069479
C	3.085080	1.395695	0.091137
C	1.987102	-2.558949	3.617824
C	-2.717687	-2.165196	-3.464999
H	-3.255841	-3.110325	-3.341570
H	-2.839362	-1.853804	-4.512882
H	-1.650706	-2.359173	-3.298865
C	-2.235105	-4.259114	-0.657134
H	-1.200987	-3.993893	-0.423766
H	-2.418319	-5.255821	-0.229783
H	-2.320334	-4.345286	-1.744330

C	1.859843	-4.396907	1.005127
C	-2.577857	0.248147	-2.883550
H	-1.488937	0.136908	-2.872357
H	-2.858900	0.578357	-3.894410
H	-2.859062	1.057221	-2.192577
C	2.520675	-0.724753	-2.235085
C	2.598664	-1.248915	4.153721
H	3.674021	-1.194423	3.949363
H	2.116814	-0.365394	3.734150
H	2.474578	-1.206652	5.245981
C	2.980859	1.581428	1.611488
H	1.953816	1.710539	1.954241
H	3.388833	0.718028	2.142686
H	3.553412	2.468922	1.917724
C	2.610680	2.693556	-0.587262
H	3.265923	3.535616	-0.321027
H	2.602800	2.633633	-1.682975
H	1.612696	2.991848	-0.214307
C	0.619981	-5.017722	1.668782
H	0.348901	-5.951498	1.153745
H	0.794310	-5.265453	2.718693
H	-0.251405	-4.356214	1.620436
C	1.342282	-1.502866	-2.849779
H	0.492522	-0.843002	-3.054961
H	1.646863	-1.947415	-3.807891
H	0.998972	-2.318608	-2.207712
C	2.759631	-3.706747	4.298215
H	2.713441	-3.571242	5.388971
H	2.334050	-4.690336	4.080596
H	3.819028	-3.723468	4.020962
C	4.093421	-2.322348	1.456681
C	0.525919	-2.658307	4.095803
H	-0.131268	-1.924110	3.629635
H	0.103675	-3.649471	3.914983
H	0.482505	-2.481666	5.180849
C	1.603898	-4.371320	-0.506123
H	0.799006	-3.678753	-0.762201
H	2.493040	-4.074115	-1.069114
H	1.316599	-5.372907	-0.859792
C	3.737218	-1.657465	-2.147336
H	3.545293	-2.512737	-1.498227
H	3.978262	-2.046647	-3.146957
H	4.627536	-1.143989	-1.771371
C	4.576907	1.192097	-0.234099
H	5.157805	2.010919	0.213204
H	4.959263	0.257244	0.187584
H	4.790731	1.190815	-1.304818
C	2.865589	0.403930	-3.222140
H	3.774130	0.946805	-2.951446
H	3.038921	-0.031076	-4.217269
H	2.052261	1.123745	-3.328449
C	3.060618	-5.332925	1.237905
H	2.819966	-6.339298	0.863846
H	3.950071	-4.987884	0.700066
H	3.326503	-5.430967	2.293553
H	4.369026	-1.293807	1.709416
H	4.423735	-2.509700	0.431058
H	4.675728	-2.983338	2.107463
H	-3.619216	3.020064	-1.075903
H	-3.056470	4.210806	-2.251419
H	-3.812617	4.742913	-0.744104

Calculated energies and coordinates of **INT10**

Electronic energy ... -3291.55100739 Eh
Total Enthalpy ... -3290.33768219 Eh
Final Gibbs free energy ... -3290.49296539 Eh

CARTESIAN COORDINATES (ANGSTROEM)

Si	-1.358854	4.019649	-0.348383
Al	-0.215259	2.176077	-1.590376
C	-0.130668	2.407514	-3.543763
H	0.780715	2.917145	-3.879420
H	-0.973014	3.040701	-3.851908
H	-0.210459	1.470272	-4.106327
C	-0.273661	5.625945	-0.537160
C	-2.901460	4.350413	-1.419975
C	-1.957586	3.641438	1.444882
C	-0.812874	3.180454	2.356088
H	-0.358893	2.257376	1.986973
H	-1.200938	2.949346	3.357770
H	-0.030756	3.936356	2.470971
C	-1.159336	6.884668	-0.492987
H	-1.723981	6.969061	0.440276
H	-1.873189	6.908051	-1.322445
H	-0.527415	7.780733	-0.578446
C	-2.971058	2.487961	1.360446
H	-3.842179	2.734708	0.742004
H	-3.335333	2.233769	2.365794
H	-2.498337	1.579282	0.962665
C	0.421949	5.601427	-1.910525
H	0.942160	6.554913	-2.082760
H	-0.287380	5.462317	-2.734709
H	1.176466	4.809573	-1.980209
C	-2.647154	4.863087	2.073293
H	-1.939671	5.678972	2.253797
H	-3.076037	4.583774	3.046200
H	-3.464419	5.249153	1.452884
C	0.799944	5.743254	0.554126
H	1.421626	4.843513	0.631865
H	0.357907	5.926518	1.538365
H	1.467008	6.588808	0.331613
Si	-2.920746	-1.625730	-0.774133
Al	-0.714748	-0.705169	0.010824
Si	1.839999	-0.170306	-0.554803
Si	2.297403	-2.376053	1.928731
O	0.296595	0.554433	-1.010273
O	0.148592	-0.033878	2.874316
C	-3.031490	-3.553841	-0.880974
C	0.175533	-0.320689	1.687583
C	-3.595449	-0.701521	-2.344015
C	-5.131631	-0.622339	-2.319751
H	-5.496444	-0.071976	-1.446618
H	-5.490133	-0.096421	-3.216722
H	-5.597566	-1.612544	-2.309932
C	-4.050289	-1.107392	0.668436
H	-4.233603	-0.028008	0.663660
H	-5.024444	-1.608227	0.615363
H	-3.597060	-1.347740	1.635699
C	-2.709350	-4.096572	0.522234
H	-3.465150	-3.800701	1.257331
H	-2.674006	-5.195256	0.503392
H	-1.735282	-3.747395	0.883549
C	1.220610	-1.124392	0.929071
C	-4.449378	-4.010708	-1.262800
H	-4.705414	-3.740220	-2.292646
H	-4.515551	-5.106059	-1.188789
H	-5.214605	-3.593206	-0.599182
C	2.990751	1.338130	-0.174641
C	2.378369	-2.107407	3.869593
C	-3.146061	-1.343568	-3.662544
H	-3.608991	-2.322930	-3.817459
H	-3.438575	-0.706204	-4.509966
H	-2.057982	-1.474645	-3.707326
C	-2.038816	-4.157274	-1.884365
H	-1.007407	-3.876635	-1.657384
H	-2.093842	-5.255075	-1.848774
H	-2.248933	-3.850416	-2.912865
C	1.780477	-4.215636	1.503824
C	-3.049318	0.732935	-2.302539
H	-1.967656	0.719378	-2.499489
H	-3.502230	1.355542	-3.088217
H	-3.240405	1.219459	-1.338138
C	2.312000	-1.064155	-2.208535
C	2.929484	-0.701622	4.177491
H	3.911983	-0.529256	3.722929
H	2.234105	0.074104	3.850507
H	3.061287	-0.599999	5.264627
C	2.828998	1.725876	1.302245

H	1.788330	1.945800	1.561371
H	3.159664	0.923112	1.964797
H	3.428653	2.621261	1.521550
C	2.586017	2.552700	-1.020623
H	3.263542	3.398763	-0.834432
H	2.581569	2.369350	-2.098917
H	1.599173	2.945226	-0.693996
C	0.698965	-4.744657	2.459169
H	0.285789	-5.685946	2.067548
H	1.100250	-4.954462	3.454031
H	-0.134839	-4.042791	2.575623
C	1.092673	-1.878353	-2.677758
H	0.230739	-1.233223	-2.881047
H	1.333483	-2.406645	-3.611190
H	0.794185	-2.628182	-1.943374
C	3.385139	-3.122657	4.454957
H	3.497734	-2.924371	5.530584
H	3.041464	-4.157126	4.356871
H	4.381482	-3.045620	4.007985
C	4.115522	-2.192741	1.405788
C	1.068602	-2.281854	4.663354
H	0.267003	-1.646372	4.287823
H	0.725843	-3.318233	4.674797
H	1.254083	-1.996109	5.709289
C	1.209021	-4.289732	0.086132
H	0.355260	-3.617026	-0.025724
H	1.953400	-4.020028	-0.668431
H	0.870727	-5.312607	-0.136509
C	3.526468	-2.000869	-2.137362
H	3.361368	-2.845065	-1.464633
H	3.719616	-2.416336	-3.136502
H	4.436256	-1.483348	-1.817591
C	4.477673	1.042517	-0.438362
H	5.083338	1.896632	-0.104851
H	4.821190	0.164625	0.115042
H	4.696784	0.881217	-1.497256
C	2.599018	-0.022075	-3.306780
H	3.524086	0.534596	-3.134103
H	2.713496	-0.542963	-4.267792
H	1.778202	0.688975	-3.427161
C	2.989834	-5.167340	1.553070
H	2.656392	-6.195485	1.348883
H	3.736224	-4.908153	0.794398
H	3.489915	-5.171687	2.525823
H	4.512960	-1.230761	1.743771
H	4.259825	-2.251605	0.325521
H	4.736915	-2.973777	1.853978
H	-3.521248	3.460012	-1.550761
H	-2.619850	4.702614	-2.418486
H	-3.528467	5.124887	-0.962876

Calculated energies and coordinates of **TS11**

Electronic energy ... -3291.55066318 Eh
Total Enthalpy ... -3290.33834072 Eh
Final Gibbs free energy ... -3290.49200270 Eh

CARTESIAN COORDINATES (ANGSTROEM)

Si	-1.354572	4.002941	-0.320549
Al	-0.207131	2.182814	-1.595832
C	-0.132588	2.421850	-3.548148
H	0.778868	2.929897	-3.885924
H	-0.974705	3.058114	-3.850425
H	-0.217441	1.486665	-4.113460
C	-0.271708	5.613069	-0.483422
C	-2.902745	4.352920	-1.377451
C	-1.946206	3.588375	1.467773
C	-0.803128	3.083574	2.357239
H	-0.381689	2.151457	1.971538
H	-1.182908	2.853204	3.362222
H	0.002659	3.815418	2.467555
C	-1.153764	6.871558	-0.387043
H	-1.694485	6.934406	0.561760
H	-1.887770	6.916170	-1.197852
H	-0.522168	7.768368	-0.466690
C	-2.983946	2.458719	1.361842
H	-3.865673	2.751269	0.779464
H	-3.325417	2.166517	2.364714
H	-2.543329	1.559379	0.909622
C	0.396879	5.627623	-1.870290
H	0.920903	6.582496	-2.021984
H	-0.330711	5.521677	-2.683374
H	1.143453	4.833246	-1.983644
C	-2.607269	4.805788	2.134281
H	-1.881090	5.599592	2.337396
H	-3.040450	4.506656	3.099341
H	-3.416750	5.228138	1.527523
C	0.821494	5.697345	0.591011
H	1.449889	4.799507	0.623937
H	0.397202	5.840698	1.589643
H	1.479537	6.554768	0.387951
Si	-2.926527	-1.622508	-0.829940
Al	-0.733215	-0.688263	-0.015692
Si	1.841351	-0.168449	-0.564049
Si	2.323425	-2.360121	1.951875
O	0.295193	0.554616	-1.019634
O	0.052478	-0.215218	2.887661
C	-3.026599	-3.550255	-0.952550
C	0.159101	-0.393670	1.680342
C	-3.591996	-0.689964	-2.398413
C	-5.129548	-0.633140	-2.397552
H	-5.515831	-0.100308	-1.522749
H	-5.481001	-0.099153	-3.292573
H	-5.581932	-1.629361	-2.409549
C	-4.070830	-1.120204	0.606458
H	-4.271945	-0.044029	0.600337
H	-5.036315	-1.636950	0.546913
H	-3.619914	-1.353856	1.576449
C	-2.727857	-4.102255	0.451660
H	-3.493958	-3.810004	1.177493
H	-2.695135	-5.200943	0.425668
H	-1.758857	-3.758231	0.829449
C	1.253218	-1.110938	0.937271
C	-4.434745	-4.010708	-1.365196
H	-4.670484	-3.737468	-2.399155
H	-4.497352	-5.106590	-1.296656
H	-5.215433	-3.599465	-0.715807
C	2.981382	1.352053	-0.211108
C	2.515234	-1.997100	3.871560
C	-3.112034	-1.310264	-3.716414
H	-3.556996	-2.295017	-3.888476
H	-3.400743	-0.668595	-4.561920
H	-2.021586	-1.424603	-3.744991
C	-2.011714	-4.141582	-1.940494
H	-0.986219	-3.862600	-1.686389
H	-2.066790	-5.239707	-1.918758
H	-2.198881	-3.822875	-2.970060
C	1.670912	-4.171122	1.616211
C	-3.070511	0.752226	-2.329702
H	-1.984406	0.760827	-2.501289
H	-3.515558	1.375911	-3.119049
H	-3.291403	1.224495	-1.364904
C	2.306203	-1.072433	-2.217986
C	2.956767	-0.536539	4.073719
H	3.872143	-0.299497	3.518077
H	2.162992	0.156180	3.785487
H	3.181986	-0.373508	5.137932
C	2.782405	1.782048	1.248167
H	1.731929	1.991757	1.476070
H	3.108648	1.004139	1.938949
H	3.363072	2.692868	1.455217
C	2.603295	2.547233	-1.095272
H	3.282876	3.393032	-0.915280
H	2.610092	2.341750	-2.168454
H	1.615470	2.956766	-0.789543
C	0.481608	-4.524297	2.525063
H	-0.038696	-5.410224	2.132665
H	0.807731	-4.760600	3.540318
H	-0.250489	-3.710807	2.594982

C	1.087246	-1.893473	-2.673292
H	0.213904	-1.257502	-2.855953
H	1.316906	-2.411735	-3.615204
H	0.814094	-2.653016	-1.938740
C	3.647430	-2.887842	4.429625
H	3.789762	-2.647791	5.493171
H	3.404294	-3.953237	4.374449
H	4.607137	-2.725841	3.929434
C	4.126043	-2.307490	1.349724
C	1.286044	-2.245521	4.766615
H	0.386699	-1.759405	4.389159
H	1.090350	-3.311300	4.903039
H	1.492866	-1.832310	5.764753
C	1.191495	-4.274181	0.165266
H	0.382393	-3.565302	-0.026410
H	1.997350	-4.070510	-0.547564
H	0.814233	-5.286832	-0.041152
C	3.518669	-2.012500	-2.159050
H	3.345245	-2.869301	-1.504666
H	3.712508	-2.409949	-3.165410
H	4.430409	-1.505674	-1.827815
C	4.469807	1.031650	-0.428368
H	5.081466	1.880882	-0.093327
H	4.782082	0.156937	0.149595
H	4.713875	0.846893	-1.478445
C	2.585777	-0.034868	-3.322081
H	3.513047	0.520500	-3.155952
H	2.694258	-0.559339	-4.281781
H	1.765084	0.676910	-3.440234
C	2.765037	-5.236992	1.800427
H	2.335968	-6.234105	1.622552
H	3.585556	-5.103726	1.087166
H	3.191687	-5.243165	2.807060
H	4.628919	-1.413639	1.734284
H	4.227482	-2.304024	0.264155
H	4.684973	-3.171453	1.722288
H	-3.520489	3.464023	-1.526055
H	-2.627630	4.729852	-2.368654
H	-3.529580	5.114191	-0.898232

Calculated energies and coordinates of **INT11**

Electronic energy ... -3291.56545125 Eh
Total Enthalpy ... -3290.35314999 Eh
Final Gibbs free energy ... -3290.50838872 Eh

CARTESIAN COORDINATES (ANGSTROEM)

Si	-1.248180	3.774498	0.068443
Al	-0.140144	2.242755	-1.556112
C	-0.204929	2.762294	-3.448824
H	0.485448	3.595358	-3.632885
H	-1.210087	3.136327	-3.682391
H	0.024611	1.979940	-4.177853
C	-0.289183	5.464604	0.072150
C	-2.932943	4.121884	-0.750031
C	-1.584654	3.024161	1.814309
C	-0.330846	2.374884	2.413035
H	0.023496	1.536051	1.800344
H	-0.557556	1.962373	3.405682
H	0.493758	3.084854	2.531494
C	-1.226667	6.614903	0.480520
H	-1.640678	6.481488	1.484138
H	-2.063052	6.724236	-0.217939
H	-0.670307	7.563464	0.476912
C	-2.649382	1.933645	1.644833
H	-3.620194	2.338653	1.342850
H	-2.779835	1.366855	2.576623
H	-2.358935	1.199193	0.871110
C	0.213124	5.754814	-1.353257
H	0.653015	6.761503	-1.397191
H	-0.591483	5.715293	-2.096931
H	0.993867	5.050976	-1.665824
C	-2.131635	4.074611	2.795664
H	-1.377796	4.827193	3.047078
H	-2.428834	3.585170	3.733951
H	-3.013668	4.591410	2.399872
C	0.921739	5.445725	1.016067
H	1.608918	4.619752	0.794809
H	0.624393	5.357813	2.065571
H	1.489664	6.381671	0.913739
Si	-3.336567	-1.022122	-1.279974
Al	-1.073469	-0.516534	-0.321293
Si	1.806861	-0.271519	-0.698503
Si	2.516630	-2.232960	1.975038
O	0.242891	0.547696	-1.079400
O	-0.465902	-1.815931	2.171550
C	-3.576804	-2.817413	-0.604843
C	0.024348	-1.290647	1.140280
C	-3.520954	-0.795582	-3.195989
C	-5.005318	-0.668948	-3.581976
H	-5.478571	0.200412	-3.114549
H	-5.093556	-0.546309	-4.671178
H	-5.582336	-1.557449	-3.304116
C	-4.681515	0.055789	-0.483593
H	-4.670074	1.080458	-0.870187
H	-5.672179	-0.365138	-0.693229
H	-4.565500	0.108869	0.601736
C	-3.688456	-2.731295	0.928905
H	-4.580749	-2.178744	1.242299
H	-3.766894	-3.745697	1.345315
H	-2.805170	-2.271684	1.395387
C	1.408726	-1.244095	0.798339
C	-4.861294	-3.450296	-1.163440
H	-4.802901	-3.623553	-2.243251
H	-5.033309	-4.425399	-0.684948
H	-5.744329	-2.831371	-0.965534
C	3.046876	1.218719	-0.564715
C	2.407888	-1.657713	3.839351
C	-2.900416	-1.938756	-4.010235
H	-3.424703	-2.885846	-3.850545
H	-2.962926	-1.706395	-5.083324
H	-1.843582	-2.095011	-3.771061
C	-2.374833	-3.712506	-0.944309
H	-1.454737	-3.343567	-0.471951
H	-2.539988	-4.726418	-0.553018
H	-2.198776	-3.796966	-2.021597
C	2.268099	-4.157872	1.788864
C	-2.805821	0.510968	-3.569579
H	-1.723610	0.424400	-3.415659
H	-2.966353	0.756605	-4.629859
H	-3.168868	1.363976	-2.979550
C	2.031276	-1.257634	-2.345909
C	2.014125	-0.173666	3.889364
H	2.746814	0.466072	3.387407
H	1.037475	-0.016372	3.423819
H	1.954911	0.158152	4.937157
C	3.160956	1.686024	0.891382
H	2.184300	1.908645	1.330563
H	3.624267	0.919720	1.513714
H	3.782028	2.592307	0.948944
C	2.544148	2.408643	-1.392038
H	3.283978	3.222063	-1.406776
H	2.312592	2.167187	-2.434825
H	1.676764	2.896860	-0.883684
C	0.814742	-4.613207	1.994595
H	0.750953	-5.703823	1.855796
H	0.435342	-4.371828	2.989219
H	0.143043	-4.135810	1.275500
C	0.811581	-2.187039	-2.454995
H	-0.126549	-1.614076	-2.525222
H	0.869891	-2.789898	-3.372414
H	0.735834	-2.867491	-1.601823
C	3.782589	-1.810951	4.518217
H	3.707404	-1.502239	5.571321
H	4.140830	-2.846101	4.506446
H	4.549446	-1.185820	4.048330
C	4.323376	-1.938362	1.440723
C	1.372511	-2.426076	4.678400
H	0.376692	-2.353056	4.236070

H	1.627825	-3.483570	4.793976
H	1.341267	-1.992896	5.689757
C	2.679924	-4.592293	0.373604
H	2.051543	-4.117073	-0.386034
H	3.729723	-4.365328	0.154435
H	2.552797	-5.680074	0.269186
C	3.296603	-2.129767	-2.331769
H	3.343245	-2.765398	-1.445734
H	3.298252	-2.791848	-3.209578
H	4.212086	-1.533281	-2.373223
C	4.458100	0.872752	-1.074158
H	5.132071	1.719047	-0.880028
H	4.873373	0.002012	-0.559671
H	4.478506	0.676840	-2.149820
C	2.056462	-0.367741	-3.596684
H	2.923875	0.300167	-3.622834
H	2.109986	-0.994548	-4.498472
H	1.150250	0.240152	-3.678735
C	3.172717	-4.921807	2.773669
H	3.072808	-6.003410	2.598641
H	4.231795	-4.670160	2.644638
H	2.906618	-4.738657	3.817827
H	4.665927	-0.927568	1.685042
H	4.450886	-2.081424	0.362390
H	5.008245	-2.630619	1.942064
H	-3.461352	3.193304	-0.989499
H	-2.809968	4.679224	-1.685163
H	-3.583453	4.713244	-0.094532

Calculated energies and coordinates of **TS12**

Electronic energy ... -3291.55623818 Eh
Total Enthalpy ... -3290.34521837 Eh
Final Gibbs free energy ... -3290.50055369 Eh

CARTESIAN COORDINATES (ANGSTROEM)

Si	-1.133409	3.903564	-0.131590
Al	-0.175467	2.169355	-1.648633
C	-0.386628	2.427700	-3.585648
H	0.539911	2.725200	-4.091876
H	-1.119361	3.225096	-3.764861
H	-0.756950	1.529065	-4.093865
C	0.047097	5.441539	-0.050246
C	-2.697812	4.516162	-1.027718
C	-1.678948	3.177680	1.572901
C	-0.571968	2.328656	2.212259
H	-0.264229	1.501139	1.559641
H	-0.928662	1.879167	3.149455
H	0.323331	2.912790	2.449084
C	-0.726594	6.700668	0.380199
H	-1.160086	6.603233	1.379700
H	-1.535675	6.939492	-0.317830
H	-0.045061	7.563602	0.400275
C	-2.877397	2.256881	1.301323
H	-3.746473	2.803689	0.919102
H	-3.179257	1.741827	2.223808
H	-2.630375	1.476221	0.565772
C	0.608525	5.700656	-1.460676
H	1.222958	6.612644	-1.458169
H	-0.186031	5.845980	-2.201868
H	1.249320	4.884378	-1.814563
C	-2.115589	4.265192	2.566581
H	-1.272872	4.887394	2.884540
H	-2.533679	3.798398	3.470030
H	-2.887649	4.922642	2.149999
C	1.216700	5.222861	0.918541
H	1.792495	4.320639	0.683543
H	0.875936	5.135940	1.955424
H	1.909652	6.075764	0.873320
Si	-3.437928	-1.272375	-0.882264
Al	-1.051013	-0.770521	-0.430505
Si	1.802819	-0.367297	-0.813522
Si	2.679571	-2.215400	2.048327
O	0.207937	0.508942	-1.083688
O	-0.649597	-1.838853	1.550250
C	-3.463604	-3.210857	-0.864484
C	0.265568	-1.568221	0.656663
C	-3.989809	-0.429290	-2.531547
C	-5.501567	-0.580322	-2.774321
H	-6.086020	-0.184626	-1.936304
H	-5.792771	-0.018318	-3.673525
H	-5.795385	-1.621646	-2.928622
C	-4.617700	-0.688957	0.480021
H	-4.854443	0.375943	0.403895
H	-5.560358	-1.246633	0.414429
H	-4.190791	-0.869566	1.470830
C	-3.443992	-3.668757	0.606236
H	-4.355190	-3.374351	1.137618
H	-3.382232	-4.765971	0.642902
H	-2.579823	-3.264503	1.146012
C	1.643580	-1.440589	0.684771
C	-4.732677	-3.757304	-1.537077
H	-4.738817	-3.574896	-2.616966
H	-4.784176	-4.845754	-1.389445
H	-5.647269	-3.327671	-1.111492
C	3.006231	1.144264	-0.654243
C	2.224520	-1.581728	3.830781
C	-3.216416	-0.981283	-3.735132
H	-3.445252	-2.036110	-3.918822
H	-3.475304	-0.422432	-4.646047
H	-2.130493	-0.894730	-3.595497
C	-2.231749	-3.806692	-1.561485
H	-1.308250	-3.539392	-1.033618
H	-2.295740	-4.904120	-1.560123
H	-2.134805	-3.482825	-2.603759
C	2.531211	-4.145511	1.883378
C	-3.692653	1.072072	-2.400873
H	-2.620585	1.260098	-2.241349
H	-3.974899	1.606994	-3.319160
H	-4.237784	1.525543	-1.566476
C	2.041879	-1.303278	-2.485703
C	1.915740	-0.077291	3.759869
H	2.790374	0.507777	3.455295
H	1.102527	0.127508	3.056495
H	1.611941	0.286734	4.752736
C	3.037218	1.604562	0.808446
H	2.037412	1.821796	1.197645
H	3.467889	0.832191	1.445539
H	3.651834	2.511755	0.908375
C	2.560904	2.334512	-1.509504
H	3.309474	3.140175	-1.484885
H	2.376102	2.091854	-2.560032
H	1.671934	2.832055	-1.055425
C	1.071423	-4.621516	1.812412
H	1.046570	-5.716106	1.699827
H	0.495198	-4.360475	2.703326
H	0.552635	-4.183874	0.953701
C	0.905911	-2.323091	-2.652345
H	-0.079274	-1.840248	-2.726889
H	1.049811	-2.892054	-3.582483
H	0.875205	-3.029243	-1.817410
C	3.406438	-1.774025	4.798800
H	3.135106	-1.386611	5.792134
H	3.679411	-2.826663	4.923417
H	4.300108	-1.232510	4.469397
C	4.517034	-1.817935	1.775527
C	0.985950	-2.276739	4.418926
H	0.120985	-2.177393	3.754996
H	1.159406	-3.341084	4.605992
H	0.736108	-1.815362	5.386297
C	3.213671	-4.584963	0.577057
H	2.706423	-4.155983	-0.292910
H	4.272037	-4.302701	0.537607
H	3.161109	-5.679621	0.480841
C	3.363245	-2.095811	-2.437708
H	3.466860	-2.657920	-1.505029
H	3.387679	-2.819576	-3.264506
H	4.240539	-1.453433	-2.544697
C	4.444428	0.789380	-1.072010

H	5.116175	1.617752	-0.805373
H	4.808053	-0.103780	-0.555897
H	4.537258	0.626792	-2.149606
C	2.052869	-0.392539	-3.720963
H	2.897940	0.304145	-3.715992
H	2.147896	-1.000657	-4.632186
H	1.128446	0.186964	-3.811919
C	3.236861	-4.864087	3.045630
H	3.221737	-5.950510	2.873247
H	4.287555	-4.565141	3.141450
H	2.744851	-4.682081	4.005485
H	4.769687	-0.785304	2.038889
H	4.803077	-1.976158	0.729988
H	5.152751	-2.467067	2.388098
H	-3.326985	3.691136	-1.373129
H	-2.438904	5.115154	-1.907413
H	-3.307026	5.145621	-0.367710

Calculated energies and coordinates of **INT12**

Electronic energy ... -3291.60333060 Eh
Total Enthalpy ... -3290.39111990 Eh
Final Gibbs free energy ... -3290.55290871 Eh

CARTESIAN COORDINATES (ANGSTROEM)

Si	-1.273428	4.285040	-0.354254
Al	-0.609793	2.591005	-2.053925
C	-0.089919	2.986102	-3.905211
H	0.848083	3.549411	-3.974333
H	-0.853064	3.599076	-4.403177
H	0.026691	2.066734	-4.491107
C	-0.025532	5.764242	-0.257334
C	-2.922579	5.011913	-0.978726
C	-1.640204	3.419139	1.341049
C	-0.469257	2.524820	1.775693
H	-0.222981	1.774986	1.012188
H	-0.722696	1.979483	2.696549
H	0.441299	3.098368	1.973756
C	-0.708147	7.032621	0.281511
H	-1.114134	6.889694	1.288103
H	-1.526959	7.361195	-0.367376
H	0.020282	7.855134	0.333227
C	-2.878727	2.528624	1.144467
H	-3.785236	3.118311	0.969227
H	-3.047886	1.909434	2.037461
H	-2.766796	1.852046	0.284911
C	0.473568	6.054054	-1.684039
H	1.103067	6.955961	-1.688803
H	-0.350775	6.226007	-2.387160
H	1.078264	5.228217	-2.076375
C	-1.950623	4.423118	2.462712
H	-1.077383	5.028296	2.725824
H	-2.262455	3.888070	3.371882
H	-2.764497	5.104045	2.187936
C	1.190995	5.439279	0.621105
H	1.682903	4.509715	0.312727
H	0.920302	5.341626	1.677268
H	1.934202	6.246873	0.548185
Si	-3.614342	-1.375248	-0.592935
Al	-1.405036	-0.310847	-0.661164
Si	2.603533	-0.537993	-0.848948
Si	2.675425	-2.508426	2.186655
O	-0.924416	0.951018	-1.680502
O	-0.189648	-1.022561	0.373942
C	-3.232655	-3.270883	-0.687555
C	1.125190	-0.978239	0.104426
C	-4.700450	-0.654387	-2.020256
C	-6.172235	-1.073908	-1.867100
H	-6.592930	-0.730433	-0.915574
H	-6.772695	-0.626918	-2.672676
H	-6.303868	-2.158624	-1.922151
C	-4.525117	-1.038580	1.038072
H	-4.907635	-0.013305	1.076127
H	-5.379122	-1.717984	1.149440
H	-3.870448	-1.180499	1.903320
C	-2.723504	-3.703592	0.699061
H	-3.506211	-3.633495	1.461836
H	-2.386836	-4.749666	0.661096
H	-1.869311	-3.097825	1.025643
C	2.217876	-1.511854	0.682489
C	-4.472838	-4.104846	-1.041357
H	-4.814699	-3.918803	-2.064931
H	-4.235935	-5.176074	-0.965291
H	-5.309516	-3.906263	-0.361175
C	3.399902	1.205850	-0.668104
C	1.676193	-1.870789	3.706676
C	-4.180321	-1.096530	-3.395148
H	-4.279391	-2.176967	-3.542759
H	-4.752482	-0.602019	-4.193467
H	-3.124906	-0.828015	-3.534279
C	-2.121303	-3.557759	-1.710412
H	-1.177024	-3.078299	-1.418374
H	-1.920949	-4.637931	-1.760475
H	-2.378014	-3.220986	-2.720369
C	2.433296	-4.373041	1.765434
C	-4.629718	0.882250	-1.950491
H	-3.610612	1.243956	-2.133271
H	-5.282493	1.321729	-2.718712
H	-4.958319	1.271030	-0.979518
C	2.939811	-1.451659	-2.505085
C	1.658765	-0.332872	3.656929
H	2.668589	0.094771	3.644522
H	1.128421	0.028797	2.770256
H	1.144287	0.062006	4.545470
C	3.400615	1.585421	0.819157
H	2.392588	1.564011	1.245157
H	4.018824	0.897661	1.405948
H	3.803325	2.601052	0.947240
C	2.576985	2.251151	-1.438890
H	3.056207	3.239835	-1.382958
H	2.448641	1.999978	-2.496311
H	1.582743	2.351932	-0.971184
C	1.061861	-4.619702	1.117198
H	0.979316	-5.668957	0.797349
H	0.235832	-4.418543	1.805258
H	0.916817	-3.985637	0.235549
C	1.930721	-2.606051	-2.614734
H	0.899762	-2.234549	-2.654659
H	2.116394	-3.176098	-3.536128
H	2.002825	-3.298255	-1.769877
C	2.347301	-2.303593	5.021326
H	1.777486	-1.911165	5.876264
H	2.391722	-3.392176	5.129167
H	3.368974	-1.916079	5.102585
C	4.510095	-2.202353	2.519316
C	0.220024	-2.359990	3.693758
H	-0.281329	-2.099402	2.754788
H	0.147927	-3.443735	3.834041
H	-0.339107	-1.887590	4.515152
C	3.523632	-4.763243	0.751517
H	3.494324	-4.123490	-0.137757
H	4.528168	-4.696388	1.183564
H	3.372973	-5.800457	0.418742
C	4.362745	-2.036334	-2.531610
H	4.542355	-2.696744	-1.675420
H	4.507860	-2.630720	-3.445376
H	5.130924	-1.257099	-2.520403
C	4.850009	1.230834	-1.177811
H	5.297222	2.215007	-0.977059
H	5.467009	0.479559	-0.670962
H	4.918813	1.055641	-2.255887
C	2.737838	-0.527775	-3.717640
H	3.464464	0.290457	-3.748175
H	2.859190	-1.102899	-4.646918
H	1.731397	-0.091578	-3.728892
C	2.580117	-5.271772	3.002107
H	2.541875	-6.329761	2.703471
H	3.535442	-5.111484	3.516522
H	1.775119	-5.108855	3.726280

H	4.672150	-1.197079	2.924160
H	5.078680	-2.275420	1.585817
H	4.935233	-2.917935	3.231805
H	-3.635639	4.224959	-1.247618
H	-2.764787	5.626861	-1.871798
H	-3.397817	5.647216	-0.221264

Calculated energies and coordinates of **INT13**

Electronic energy ... -3404.91383900 Eh
Total Enthalpy ... -3403.69166978 Eh
Final Gibbs free energy ... -3403.85937057 Eh

CARTESIAN COORDINATES (ANGSTROEM)

Si	-1.981405	3.671848	-2.812002
Al	-1.150384	1.361811	-2.502631
C	-0.529233	0.145322	-3.911593
H	-0.698838	-0.904421	-3.643801
H	0.550958	0.258880	-4.076650
H	-1.017127	0.338182	-4.874547
C	-1.034990	4.897432	-1.650499
C	-1.752666	4.277285	-4.599388
C	-3.892552	3.547910	-2.500201
C	-4.210833	3.393693	-1.006129
H	-3.621866	2.589232	-0.548060
H	-5.273990	3.146712	-0.867493
H	-4.015345	4.316934	-0.450191
C	-1.715616	6.270978	-1.558405
H	-2.681929	6.211773	-1.046447
H	-1.883048	6.713353	-2.547369
H	-1.085341	6.969428	-0.988174
C	-4.392935	2.287973	-3.230453
H	-4.152816	2.303059	-4.300296
H	-5.485490	2.200059	-3.137554
H	-3.966985	1.369792	-2.800313
C	0.371444	5.083282	-2.246420
H	0.989431	5.693607	-1.571833
H	0.339741	5.587843	-3.217887
H	0.888336	4.123268	-2.382165
C	-4.647268	4.761722	-3.064124
H	-4.331823	5.697851	-2.592686
H	-5.727442	4.650531	-2.887606
H	-4.499541	4.863937	-4.144819
C	-0.880406	4.314000	-0.238134
H	-0.402089	3.327872	-0.273980
H	-1.841542	4.192776	0.271405
H	-0.252751	4.972680	0.380795
Si	-3.064871	-0.657882	1.959051
Al	-1.121521	-0.048886	0.571954
Si	2.910593	0.265747	0.957177
Si	3.162391	-3.200078	-0.100496
O	-1.262181	0.771719	-0.909482
O	0.201853	-1.208735	0.699997
O	0.070565	2.190839	2.625207
C	-2.450382	-1.751880	3.431848
C	1.478896	-0.866616	0.668698
C	-4.308689	-1.507380	0.740497
C	-5.435016	-2.248187	1.475877
H	-5.961234	-1.597415	2.184131
H	-6.178125	-2.615420	0.752671
H	-5.059591	-3.116654	2.026728
C	-3.925370	0.860519	2.712111
H	-4.088064	1.646827	1.967693
H	-4.902308	0.592888	3.133018
H	-3.327743	1.293151	3.522241
C	-1.224894	-1.056147	4.052361
H	-1.448628	-0.032061	4.377213
H	-0.884363	-1.612080	4.938208
H	-0.383817	-1.024618	3.349063
C	2.642375	-1.478272	0.397873
C	-3.529511	-1.890334	4.518434
H	-4.432710	-2.382460	4.144311
H	-3.144194	-2.496633	5.351284
H	-3.821135	-0.917128	4.928830
C	3.679966	0.447992	2.713556
C	2.032034	-4.489643	0.780477
C	-3.580738	-2.487975	-0.193935
H	-3.076546	-3.292858	0.349737
H	-4.297064	-2.951880	-0.887743
H	-2.825899	-1.975716	-0.806649
C	-2.018088	-3.146035	2.957237
H	-1.273352	-3.087880	2.154965
H	-1.563248	-3.703336	3.789427
H	-2.866993	-3.735838	2.595569
C	3.153765	-3.269250	-2.024110
C	-0.095045	1.425997	1.803421
C	-4.924090	-0.394166	-0.124168
H	-4.148465	0.196612	-0.627099
H	-5.564794	-0.832352	-0.903364
H	-5.540285	0.293760	0.464761
C	3.342519	1.618042	-0.341277
C	1.825433	-4.037118	2.237306
H	2.776113	-3.903600	2.768558
H	1.270138	-3.095620	2.288905
H	1.246807	-4.796254	2.783976
C	2.777838	-0.292053	3.714212
H	1.809937	0.207072	3.836113
H	2.587962	-1.324435	3.403471
H	3.261895	-0.319351	4.700937
C	3.864396	1.894888	3.190390
H	4.255365	1.893881	4.217854
H	4.583695	2.442147	2.572566
H	2.922056	2.454058	3.198150
C	1.853991	-2.678984	-2.593695
H	1.908398	-2.632908	-3.691086
H	0.977448	-3.279366	-2.331317
H	1.675930	-1.663517	-2.224306
C	2.597999	1.279359	-1.641453
H	1.511822	1.258971	-1.477154
H	2.816700	2.038211	-2.406613
H	2.883794	0.302540	-2.041023
C	2.712404	-5.870217	0.798019
H	2.065239	-6.595672	1.312016
H	2.900665	-6.255251	-0.209677
H	3.668182	-5.846807	1.332935
C	4.932211	-3.486113	0.495618
C	0.651956	-4.619754	0.117385
H	0.147230	-3.650814	0.038646
H	0.719137	-5.057484	-0.884181
H	0.012336	-5.282345	0.719643
C	4.337223	-2.415949	-2.516513
H	4.306971	-1.401458	-2.102674
H	5.300901	-2.859884	-2.243547
H	4.308610	-2.329703	-3.612264
C	4.857505	1.614583	-0.610552
H	5.213774	0.626704	-0.925607
H	5.099471	2.327694	-1.411864
H	5.428446	1.913702	0.275567
C	5.053832	-0.248219	2.691254
H	5.513618	-0.198861	3.689057
H	4.960675	-1.303191	2.415143
H	5.746310	0.224382	1.985040
C	2.910185	3.026684	0.097190
H	3.442781	3.374381	0.984971
H	3.114841	3.742418	-0.712056
H	1.835708	3.076990	0.305150
C	3.338803	-4.699563	-2.552920
H	3.438376	-4.684894	-3.648127
H	4.241512	-5.172953	-2.148250
H	2.483591	-5.338981	-2.311926
H	4.963780	-3.636176	1.580523
H	5.566780	-2.624665	0.262762
H	5.381056	-4.370942	0.030098
H	-2.392672	3.714354	-5.287589
H	-0.720102	4.144616	-4.937321
H	-2.007001	5.338816	-4.706034

Calculated energies and coordinates of **TS13**

Electronic energy ... -3404.90291476 Eh
Total Enthalpy ... -3403.68124875 Eh
Final Gibbs free energy ... -3403.84701814 Eh

CARTESIAN COORDINATES (ANGSTROEM)

Si	-2.109650	3.863038	-2.840080
Al	-1.164492	1.590666	-2.575961
C	-0.213743	0.580083	-3.971196
H	-0.485676	0.887705	-4.987442
H	-0.396582	-0.497980	-3.880438
H	0.871860	0.723083	-3.873963
C	-0.609647	5.092364	-2.774067
C	-2.951512	4.097205	-4.530384
C	-3.469617	4.132694	-1.491927
C	-2.859498	4.259753	-0.089258
H	-2.195136	3.416843	0.140029
H	-3.655345	4.271649	0.669938
H	-2.285921	5.185924	0.024349
C	-1.046706	6.556198	-2.619897
H	-1.495780	6.746378	-1.639403
H	-1.771600	6.850370	-3.387910
H	-0.176781	7.223028	-2.716358
C	-4.379853	2.890839	-1.508710
H	-4.812643	2.706343	-2.499777
H	-5.215974	3.029404	-0.807323
H	-3.833422	1.991795	-1.197994
C	0.155575	4.944989	-4.101067
H	1.081122	5.538528	-4.071192
H	-0.435197	5.295269	-4.953973
H	0.440217	3.901841	-4.296707
C	-4.333236	5.368490	-1.791298
H	-3.747231	6.292762	-1.795648
H	-5.114406	5.476257	-1.024218
H	-4.836263	5.284337	-2.761039
C	0.348701	4.729398	-1.629846
H	0.760387	3.719451	-1.756941
H	-0.132554	4.766619	-0.647621
H	1.203171	5.422351	-1.609031
Si	-3.372380	-0.720920	1.516909
Al	-1.282034	0.080534	0.470352
Si	2.295769	0.104069	1.376812
Si	3.329241	-3.124118	-0.103372
O	-1.275703	0.880061	-1.039631
O	-0.146195	-1.470054	0.266194
O	0.291253	1.771655	2.384622
C	-2.968232	-1.316636	3.316081
C	1.065979	-1.466866	0.432178
C	-4.159375	-2.071347	0.364232
C	-5.640793	-2.309128	0.699470
H	-6.232356	-1.395160	0.579131
H	-6.062911	-3.065641	0.021045
H	-5.784041	-2.669034	1.723102
C	-4.659597	0.671527	1.668502
H	-5.093490	0.910041	0.692251
H	-5.482138	0.388815	2.337057
H	-4.212425	1.592282	2.056883
C	-2.710353	-0.055375	4.160706
H	-3.614648	0.552369	4.271368
H	-2.376086	-0.340412	5.169223
H	-1.930484	0.581343	3.723413
C	2.345862	-1.621824	0.522660
C	-4.121555	-2.103681	3.954357
H	-4.287106	-3.065162	3.456961
H	-3.895968	-2.315930	5.010177
H	-5.063022	-1.542541	3.927137
C	2.943733	-0.051933	3.180991
C	2.537436	-4.677804	0.716506
C	-3.401603	-3.403526	0.442855
H	-3.509918	-3.880676	1.422742
H	-3.793443	-4.106611	-0.307245
H	-2.329728	-3.275570	0.246369
C	-1.694048	-2.174849	3.344717
H	-0.831819	-1.617316	2.960094
H	-1.455550	-2.468695	4.378073
H	-1.788647	-3.089234	2.750230
C	3.268476	-3.086373	-2.028308
C	0.281359	0.864478	1.595207
C	-4.074382	-1.555474	-1.084228
H	-3.035151	-1.448892	-1.419692
H	-4.573572	-2.260392	-1.765619
H	-4.562475	-0.580282	-1.204471
C	3.094079	1.411259	0.210274
C	2.339213	-4.411677	2.218776
H	3.273389	-4.133849	2.720622
H	1.608018	-3.616799	2.394983
H	1.965805	-5.322508	2.707248
C	1.864965	-0.734739	4.037832
H	0.967839	-0.116388	4.135774
H	1.573390	-1.706111	3.624051
H	2.259262	-0.913916	5.048049
C	3.265552	1.323962	3.784494
H	3.532736	1.194569	4.842904
H	4.122511	1.793481	3.290268
H	2.413447	2.007725	3.734128
C	1.873615	-2.713061	-2.555038
H	1.880314	-2.712229	-3.654009
H	1.101432	-3.418375	-2.231136
H	1.568109	-1.712515	-2.233073
C	2.410261	1.270579	-1.158912
H	1.330383	1.445556	-1.078386
H	2.823761	2.009056	-1.860563
H	2.546385	0.278188	-1.596109
C	3.478042	-5.889303	0.574056
H	3.035690	-6.757767	1.082018
H	3.647789	-6.172641	-0.468522
H	4.453035	-5.701872	1.037300
C	5.128216	-3.011613	0.438003
C	1.167252	-5.023146	0.112659
H	0.463828	-4.184499	0.176188
H	1.240192	-5.325833	-0.936448
H	0.720019	-5.862181	0.664341
C	4.276784	-2.034694	-2.525922
H	4.075081	-1.041498	-2.112983
H	5.308301	-2.301940	-2.272236
H	4.214920	-1.952100	-3.620057
C	4.600642	1.148327	0.063512
H	4.804717	0.126597	-0.273904
H	5.029992	1.835448	-0.679940
H	5.135719	1.309269	1.005963
C	4.209505	-0.919828	3.209640
H	4.587955	-0.986390	4.239850
H	3.996182	-1.934971	2.865817
H	5.012162	-0.510901	2.586104
C	2.880086	2.856060	0.692832
H	3.369543	3.060088	1.648085
H	3.308505	3.542285	-0.051407
H	1.820855	3.106021	0.797974
C	3.678318	-4.445759	-2.621563
H	3.759378	-4.357552	-3.714174
H	4.652415	-4.785807	-2.250089
H	2.941904	-5.227937	-2.413487
H	5.261477	-3.283866	1.488985
H	5.532749	-2.004457	0.300889
H	5.738752	-3.698259	-0.159872
H	-3.902369	3.555214	-4.572760
H	-2.326324	3.722615	-5.346957
H	-3.165533	5.154262	-4.730605

Calculated energies and coordinates of **INT14**

Electronic energy ... -3404.90386492 Eh
Total Enthalpy ... -3403.68065994 Eh
Final Gibbs free energy ... -3403.84666605 Eh

CARTESIAN COORDINATES (ANGSTROEM)

Si	-2.262041	4.093322	-2.709196
Al	-1.239726	1.844755	-2.841559
C	-0.184915	1.151683	-4.350335

H	-0.605109	1.418116	-5.327363
H	-0.086328	0.060394	-4.304685
H	0.832142	1.567327	-4.324369
C	-0.785231	5.292935	-2.331573
C	-3.048149	4.669542	-4.341922
C	-3.667843	4.068095	-1.379914
C	-3.107939	3.943466	0.043709
H	-2.450817	3.070933	0.148672
H	-3.932728	3.824277	0.761318
H	-2.540466	4.830536	0.343949
C	-1.247109	6.672340	-1.840508
H	-1.720379	6.619209	-0.854740
H	-1.959153	7.135227	-2.533989
H	-0.385106	7.350591	-1.753509
C	-4.536877	2.829698	-1.664750
H	-4.942584	2.833692	-2.684035
H	-5.391394	2.799151	-0.972376
H	-3.965236	1.903581	-1.527156
C	0.003346	5.478079	-3.640022
H	0.917271	6.059596	-3.449945
H	-0.580020	6.013724	-4.396080
H	0.309215	4.515818	-4.073122
C	-4.560552	5.316899	-1.468366
H	-4.004408	6.239133	-1.272815
H	-5.366678	5.253092	-0.722743
H	-5.030994	5.410011	-2.453497
C	0.162074	4.673046	-1.292429
H	0.609262	3.741055	-1.667784
H	-0.332783	4.439203	-0.344238
H	0.996641	5.356817	-1.076528
Si	-3.222503	-0.736220	1.276029
Al	-1.233154	0.135267	0.080142
Si	2.282778	0.169802	1.124521
Si	3.258004	-3.252361	0.152779
O	-1.352722	0.867569	-1.458780
O	-0.119692	-1.515705	-0.328800
O	0.297722	1.954194	1.734208
C	-2.621100	-1.426221	2.987160
C	1.019962	-1.618138	0.039086
C	-4.138945	-2.028736	0.153813
C	-5.555850	-2.329851	0.668483
H	-6.168865	-1.423422	0.720603
H	-6.061265	-3.029885	-0.013617
H	-5.548493	-2.787369	1.662542
C	-4.483772	0.633510	1.657927
H	-4.996983	0.959854	0.747579
H	-5.244924	0.284777	2.366621
H	-3.998979	1.514011	2.090175
C	-2.284232	-0.210208	3.868937
H	-3.182952	0.351773	4.143509
H	-1.800669	-0.541594	4.799952
H	-1.597843	0.484454	3.367817
C	2.240840	-1.658474	0.444678
C	-3.681565	-2.272610	3.705855
H	-3.878392	-3.213818	3.181658
H	-3.337136	-2.528120	4.719284
H	-4.632651	-1.737503	3.809322
C	2.900815	0.202857	2.945187
C	2.392259	-4.646695	1.161339
C	-3.354189	-3.343243	0.038529
H	-3.306359	-3.877559	0.993394
H	-3.841494	-4.012214	-0.686241
H	-2.327011	-3.174683	-0.307767
C	-1.343841	-2.263391	2.823603
H	-0.531126	-1.664043	2.394914
H	-0.994862	-2.626857	3.802250
H	-1.492104	-3.134834	2.177093
C	3.332240	-3.540159	-1.752894
C	0.386860	0.900832	1.106724
C	-4.260646	-1.416745	-1.253527
H	-3.278444	-1.237698	-1.707447
H	-4.817192	-2.098339	-1.913958
H	-4.798050	-0.460743	-1.241655
C	3.203153	1.251584	-0.177250
C	2.113430	-4.134026	2.584782
H	3.024276	-3.800612	3.095434
H	1.399316	-3.305946	2.580810
H	1.679915	-4.943817	3.188220
C	1.785798	-0.321654	3.863803
H	0.891567	0.306378	3.818775
H	1.499519	-1.347986	3.614750
H	2.141808	-0.323176	4.903759
C	3.231340	1.649917	3.351041
H	3.443329	1.674705	4.429365
H	4.123828	2.018338	2.835741
H	2.400662	2.334191	3.153255
C	1.988755	-3.279572	-2.451842
H	2.077844	-3.521393	-3.520033
H	1.172841	-3.886747	-2.046690
H	1.692634	-2.227651	-2.386457
C	2.502402	1.010020	-1.526662
H	1.439754	1.280425	-1.482334
H	2.971774	1.628157	-2.304633
H	2.561041	-0.034001	-1.848315
C	3.307200	-5.879743	1.276716
H	2.818441	-6.639705	1.902295
H	3.519550	-6.341798	0.309054
H	4.263468	-5.633595	1.751446
C	5.025416	-3.076395	0.773974
C	1.050362	-5.064188	0.539637
H	0.367763	-4.213884	0.422176
H	1.173830	-5.539363	-0.438419
H	0.547296	-5.788847	1.195269
C	4.376267	-2.582292	-2.355199
H	4.131063	-1.533678	-2.160062
H	5.383841	-2.772657	-1.970347
H	4.409420	-2.712304	-3.445967
C	4.685984	0.872728	-0.292032
H	4.820489	-0.189238	-0.520212
H	5.158943	1.446910	-1.102033
H	5.233944	1.095833	0.630303
C	4.147662	-0.666728	3.140887
H	4.496991	-0.578305	4.179734
H	3.925762	-1.719991	2.955616
H	4.974143	-0.370845	2.486102
C	3.091842	2.752711	0.144282
H	3.678163	3.035848	1.021089
H	3.479817	3.326209	-0.709532
H	2.058964	3.067225	0.315712
C	3.779245	-4.980692	-2.060429
H	3.952635	-5.087338	-3.140378
H	4.715115	-5.244005	-1.553630
H	3.019841	-5.716554	-1.779041
H	5.106015	-3.237487	1.852147
H	5.449818	-2.094073	0.549306
H	5.652370	-3.829676	0.282683
H	-3.985931	4.139149	-4.539466
H	-2.384092	4.487158	-5.192672
H	-3.274791	5.742637	-4.318493

Calculated energies and coordinates of **5’**

Electronic energy ... -3404.97862954 Eh
Total Enthalpy ... -3403.75462508 Eh
Final Gibbs free energy ... -3403.92031083 Eh

CARTESIAN COORDINATES (ANGSTROEM)

Si	-2.142316	4.036447	-2.396668
Al	-0.975751	1.878420	-2.739132
C	0.546251	1.528031	-3.936991
H	1.220859	2.387470	-4.033837
H	0.210711	1.278208	-4.952689
H	1.131678	0.672896	-3.576601
C	-1.565248	5.351821	-3.694414
C	-4.024367	3.846283	-2.591850
C	-1.806540	4.502855	-0.540780
C	-0.335063	4.251016	-0.171803
H	-0.073718	3.189678	-0.282196
H	-0.156584	4.509788	0.882835

H	0.358498	4.839600	-0.781863
C	-2.492512	6.577585	-3.722965
H	-2.502010	7.113150	-2.768679
H	-3.524475	6.296824	-3.960830
H	-2.157414	7.285226	-4.495845
C	-2.680241	3.579873	0.327766
H	-3.746448	3.799834	0.208006
H	-2.428038	3.710254	1.390241
H	-2.525336	2.522492	0.078651
C	-1.612539	4.680865	-5.079507
H	-1.344396	5.407164	-5.860934
H	-2.612263	4.298758	-5.318561
H	-0.904820	3.846047	-5.149587
C	-2.164153	5.960389	-0.216043
H	-1.500873	6.668811	-0.723348
H	-2.069767	6.139840	0.865467
H	-3.195999	6.200133	-0.498555
C	-0.123814	5.809545	-3.432411
H	0.568464	4.959710	-3.376981
H	-0.036775	6.377131	-2.499895
H	0.221857	6.462709	-4.247288
Si	-3.560510	-1.103465	0.743913
Al	-1.492240	-0.329772	-0.327693
Si	2.567681	0.058093	1.535413
Si	2.966080	-3.209982	0.126141
O	-1.511996	0.601680	-1.756750
O	-0.136249	-1.600950	-0.376951
O	-0.358800	0.755408	0.877974
C	-3.398324	-0.967941	2.670298
C	0.898950	-1.125330	0.269618
C	-3.945018	-2.882136	0.073642
C	-5.424026	-3.254834	0.262259
H	-6.086377	-2.560525	-0.266205
H	-5.612794	-4.261377	-0.139925
H	-5.718104	-3.261200	1.316948
C	-5.009285	0.008244	0.211333
H	-5.224195	-0.126854	-0.854464
H	-5.927258	-0.214528	0.768661
H	-4.772826	1.066846	0.360112
C	-3.398548	0.530708	3.022324
H	-4.352278	1.008612	2.773939
H	-3.231954	0.662834	4.101678
H	-2.602453	1.073392	2.496817
C	2.186105	-1.553844	0.569802
C	-4.563095	-1.645454	3.406971
H	-4.555303	-2.732115	3.271112
H	-4.493850	-1.449861	4.487629
H	-5.535255	-1.269058	3.067619
C	2.727744	-0.057998	3.439179
C	1.749310	-4.650404	0.549872
C	-3.061811	-3.946844	0.738315
H	-3.314196	-4.087149	1.794870
H	-3.198429	-4.917300	0.237832
H	-1.997602	-3.690116	0.671898
C	-2.072635	-1.569509	3.164276
H	-1.213656	-1.072743	2.696771
H	-1.975853	-1.434434	4.252215
H	-1.992842	-2.640520	2.954738
C	3.518821	-3.141448	-1.722214
C	0.752252	0.163798	0.930145
C	-3.634569	-2.878893	-1.434911
H	-2.562867	-2.738244	-1.627888
H	-3.921186	-3.842035	-1.882736
H	-4.178146	-2.091306	-1.970482
C	3.657753	1.354276	0.646331
C	1.019340	-4.320107	1.863688
H	1.715359	-4.170483	2.696818
H	0.399872	-3.421820	1.770708
H	0.356731	-5.154228	2.136227
C	2.128770	-1.397588	3.893283
H	1.090001	-1.521175	3.567459
H	2.701307	-2.244742	3.503253
H	2.143958	-1.456020	4.990534
C	1.905147	1.082220	4.070233
H	1.945075	0.998438	5.165377
H	2.284992	2.072791	3.801830
H	0.851143	1.033100	3.772634
C	2.392423	-2.605766	-2.620440
H	2.741788	-2.550414	-3.661416
H	1.502144	-3.239779	-2.599902
H	2.081538	-1.599048	-2.320063
C	3.132659	1.495973	-0.794485
H	2.102132	1.872643	-0.812938
H	3.754551	2.212744	-1.347918
H	3.146142	0.548102	-1.341641
C	2.548885	-5.949763	0.762663
H	1.857109	-6.766637	1.012790
H	3.101861	-6.252070	-0.132636
H	3.263933	-5.861600	1.587803
C	4.504246	-3.384764	1.210458
C	0.689305	-4.891360	-0.536722
H	0.112026	-3.987567	-0.751335
H	1.130521	-5.250406	-1.471595
H	-0.013588	-5.663942	-0.192498
C	4.723730	-2.194320	-1.843841
H	4.471966	-1.178081	-1.525907
H	5.582319	-2.535641	-1.254708
H	5.044483	-2.136440	-2.893753
C	5.133990	0.925298	0.610577
H	5.266382	-0.059924	0.151193
H	5.714800	1.643779	0.015142
H	5.577227	0.891986	1.610350
C	4.182193	0.020881	3.926991
H	4.212323	-0.114985	5.017185
H	4.807275	-0.762753	3.481981
H	4.640759	0.990026	3.707122
C	3.537539	2.724290	1.335130
H	3.959591	2.719855	2.345064
H	4.088833	3.476544	0.753559
H	2.494966	3.059643	1.398660
C	3.959705	-4.528156	-2.218587
H	4.353345	-4.442926	-3.241710
H	4.757284	-4.953491	-1.597575
H	3.132747	-5.243302	-2.247562
H	4.237506	-3.603524	2.250418
H	5.090062	-2.458370	1.206722
H	5.158888	-4.192476	0.865214
H	-4.385707	2.958046	-2.063555
H	-4.305499	3.738293	-3.644882
H	-4.560173	4.716634	-2.193263

Calculated energies and coordinates of **4''**

Electronic energy ... -3178.23033889 Eh
Total Enthalpy ... -3177.03044325 Eh
Final Gibbs free energy ... -3177.18785042 Eh

CARTESIAN COORDINATES (ANGSTROEM)

Si	-5.471853	-0.821451	0.183008
Si	-0.437236	-0.425590	-1.258591
Si	-0.440534	0.537016	1.904913
Al	-3.035469	-0.379081	0.409656
Al	2.857863	-0.481837	-1.203211
Si	4.493898	0.687044	-2.649338
O	1.189500	-0.165047	-1.276579
C	-1.231603	-0.125668	0.374764
C	-5.862971	-2.459520	1.123420
C	-0.662214	-2.276498	-1.814294
C	-6.486527	0.741185	0.697798
C	4.108758	2.571213	-2.861850
C	-5.779829	-1.096589	-1.668710
H	-5.667736	-0.162578	-2.228968
H	-6.800890	-1.462942	-1.831424
H	-5.090103	-1.825738	-2.102904
C	-1.133609	0.827648	-2.567828
C	3.575095	-1.818569	0.048394
H	2.821131	-2.376599	0.611241
H	4.233057	-1.327861	0.779696
H	4.205949	-2.544213	-0.482490

C	-5.746306	1.991166	0.190703
H	-4.796459	2.144481	0.717095
H	-6.361569	2.885967	0.361789
H	-5.532423	1.941365	-0.883834
C	-1.476255	2.052879	2.578448
C	-7.874572	0.700730	0.032709
H	-7.802163	0.721035	-1.059451
H	-8.454847	1.582360	0.339947
H	-8.450099	-0.186362	0.317276
C	-0.253794	-0.862248	3.251664
C	-7.372320	-2.753577	1.111298
H	-7.939151	-2.017574	1.691055
H	-7.562014	-3.738913	1.560445
H	-7.781249	-2.775361	0.094462
C	0.293597	-2.659620	-2.955753
H	0.157605	-2.051162	-3.853157
H	0.145281	-3.712034	-3.239139
H	1.340705	-2.561922	-2.640670
C	-0.346040	-3.201870	-0.629138
H	0.685611	-3.067756	-0.284458
H	-0.458527	-4.254106	-0.930485
H	-1.009650	-3.011353	0.220630
C	1.306339	1.213586	1.616602
H	1.964649	0.417744	1.254613
H	1.741088	1.605992	2.542744
H	1.304023	2.014723	0.869874
C	-0.649125	0.514528	-3.992034
H	0.436620	0.369204	-4.024287
H	-0.890720	1.348588	-4.667719
H	-1.124491	-0.382838	-4.403043
C	-6.662420	0.857088	2.218038
H	-7.283953	0.050652	2.620320
H	-7.158274	1.806903	2.463837
H	-5.702968	0.843130	2.747135
C	3.325329	-0.549542	-4.975630
H	2.845991	0.383350	-5.286813
H	2.627070	-1.074140	-4.310943
H	3.453100	-1.172387	-5.873200
C	-5.364642	-2.419443	2.575297
H	-4.280012	-2.265283	2.624897
H	-5.576707	-3.377875	3.070132
H	-5.843616	-1.631849	3.164076
C	5.275430	-1.688730	-3.935138
H	5.340627	-2.319071	-4.833844
H	4.650853	-2.224551	-3.207326
H	6.282783	-1.606075	-3.513870
C	-2.663414	0.912728	-2.580215
H	-3.140866	-0.026616	-2.880022
H	-3.004925	1.689899	-3.280491
H	-3.053479	1.202908	-1.590138
C	4.683941	-0.315039	-4.297804
C	6.158902	0.569129	-1.731477
H	6.174003	1.234155	-0.860565
H	7.001801	0.849282	-2.375016
H	6.340245	-0.445967	-1.363569
C	-0.588448	2.210011	-2.169770
H	-0.916049	2.493803	-1.165682
H	-0.942889	2.976780	-2.874799
H	0.505080	2.223943	-2.181671
C	-2.102500	-2.549208	-2.259579
H	-2.832676	-2.229867	-1.495257
H	-2.270590	-3.626548	-2.406415
H	-2.354521	-2.040839	-3.195908
C	-2.822243	1.583900	3.136453
H	-3.366066	0.933578	2.420405
H	-3.494143	2.433453	3.330341
H	-2.716575	1.015057	4.065407
C	-0.737626	2.845771	3.671909
H	-0.502723	2.246094	4.553626
H	-1.357483	3.691811	4.004096
H	0.199679	3.263720	3.289670
C	3.587975	3.085873	-1.507814
H	2.639250	2.608880	-1.233736
H	3.411116	4.170619	-1.553972
H	4.303205	2.904991	-0.695615
C	-1.750170	3.036260	1.429097
H	-0.819034	3.416847	0.994052
H	-2.320050	3.900606	1.801749
H	-2.323135	2.570829	0.620039
C	-5.130721	-3.601840	0.397595
H	-5.479953	-3.730524	-0.632317
H	-5.302546	-4.548763	0.928025
H	-4.044857	-3.438753	0.368781
C	5.373120	3.365704	-3.228875
H	6.141508	3.287449	-2.452273
H	5.125185	4.431380	-3.342080
H	5.814198	3.027808	-4.172323
C	-1.519011	-1.722022	3.370592
H	-2.387452	-1.146336	3.712344
H	-1.363784	-2.533450	4.097174
H	-1.768712	-2.186148	2.408472
C	3.031672	2.820121	-3.925968
H	3.397335	2.597985	-4.934240
H	2.724467	3.876348	-3.914555
H	2.134848	2.215277	-3.751130
C	5.630918	0.375659	-5.290549
H	6.610103	0.586073	-4.844715
H	5.216993	1.319901	-5.659575
H	5.799436	-0.270958	-6.164592
C	0.106902	-0.334305	4.648362
H	0.970111	0.340907	4.625122
H	0.367517	-1.173988	5.309822
H	-0.728464	0.196205	5.116985
C	0.887584	-1.782251	2.784333
H	0.718300	-2.134773	1.762376
H	0.954399	-2.664751	3.437851
H	1.858683	-1.277283	2.815115

Calculated energies and coordinates of **CO**

Electronic energy ... -113.29360648 Eh
Total Enthalpy ... -113.28530425 Eh
Final Gibbs free energy ... -113.30773634 Eh

CARTESIAN COORDINATES (ANGSTROEM)

C	0.000000	0.000000	0.000000
O	0.000000	0.000000	1.130425

8. References

[S1] Réant B. L. L.; Berryman V. E. J.; Basford A. R.; Nodaraki L. E.; Wooles A. J.; Tuna F.; Kaltsoyannis N.; Mills D. P.; Liddle S. T.; *J. Am. Chem. Soc.* **2021**, *143*, 9813.

[S2] Hicks J.; Juckel M.; Paparo A.; Dange D.; Jones C. *Organometallics* **2018**, *37*, 4810.

[S3] a) Sheldrick, G. M. SADABS, Bruker AXS, Madison, USA, **2007**; b) CrysAlisPro, Scale3 Abspack, Rigaku Oxford Diffraction, **2019.**; Clark, R. C.; Reid, J. S., *Acta Crystallogr. A* **1995**, *51*, 887.

[S4] Dolomanov, O. V.; Bourhis, L. J.; Gildea, R. J.; Howard, J. A. K.; Puschmann, H., *J. Appl. Crystallogr*. **2009**, *42*, 339.

[S5] Sheldrick, G., *Acta Crystallogr. A* **2015**, *71*, 3.

[S6] Sheldrick, G. M., *Acta crystallographica. Section C, Structural chemistry* **2015**, *71*, 3.

[S7] Sheldrick, G. M., *Acta Crystallogr. A* **2008**, *64*, 112.

[S8] Neese, F. *WIREs Computational Molecular Science* **2022**, *12* (5), e1606..

[S9] Furness, J. W.; Kaplan, A. D.; Ning, J.; Perdew, J. P.; Sun, J. *J Phys Chem. Lett.* **2020**, *11*, 9248.

[S10] Furness, J. W.; Kaplan, A. D.; Ning, J.; Perdew, J. P.; Sun, J. *J. Phys. Chem. Lett.* **2020**, *11*, 22.

[S11] Kruse, H.; Grimme, S. *Journal of Chemical Physics* **2012**, *136* (15).

[S12] Caldeweyher, E.; Bannwarth, C.; Grimme, S. *Journal of Chemical Physics* **2017**, *147* (3).

[S13] Caldeweyher, E.; Ehlert, S.; Hansen, A.; Neugebauer, H.; Spicher, S.; Bannwarth, C.; Grimme, S. A *Journal of Chemical Physics* **2019**, *150* (15).

[S14] Caldeweyher, E.; Mewes, J. M.; Ehlert, S.; Grimme, S. *Physical Chemistry Chemical Physics* **2020**, *22*, 8499.

[S15] Grimme, S.; Hansen, A.; Ehlert, S.; Mewes, J. M. R. *J Chem Phys* **2021**, *154* (6).

[S16] Marenich, A. V.; Cramer, C. J.; Truhlar, D. G. *Journal of Physical Chemistry B* **2009**, *113*, 6378.

[S17] Zhao, Y.; Truhlar, D. G. *J. Phys. Chem. A* **2005**, *109*, 5656.

[S18] Weigend, F.; Ahlrichs, R. *Physical Chemistry Chemical Physics* **2005**, *7*, 3297.

[S19] Weigend, F. *Physical Chemistry Chemical Physics* **2006**, *8*, 1057.

[S20] Hellweg, A.; Hättig, C.; Höfener, S.; Klopper, W. *Theor Chem Acc* **2007**, *117*, 587.

[S21] NBO 7.0. *NBO 7.0. E. D. Glendening, J, K. Badenhoop, A. E. Reed, J. E. Carpenter, J. A. Bohmann, C. M. Morales, P. Karafiloglou, C. R. Landis, and F. Weinhold, Theoretical Chemistry Institute, University of Wisconsin, Madison (2018).* NBO 7.0. E. D. Glendening, J, K. Badenhoop, A. E. Reed, J. E. Carpenter, J. A. Bohmann, C. M. Morales, P. Karafiloglou, C. R. Landis, and F. Weinhold, Theoretical Chemistry Institute, University of Wisconsin, Madison (2018). 2018.

[S22] Adamo, C.; Barone, V. *J Chem Phys* **1999**, *110*, 6158.

[S23] (a) Frisch, M. J.; Pople, J. A.; Binkley, J. S. *J Chem Phys* **1984**, *80* (7), 3265–3269.
(b) Clark, T.; Chandrasekhar, J.; Spitznagel, G. W.; Schleyer, P. V. R. *J Comput Chem* **1983**, *4* (3), 294–301; (c) Curtiss, L. A.; McGrath, M. P.; Blaudeau, J. P.; Davis, N. E.; Binning, R. C.; Radom, L. *J. Chem. Phys.* **1995**, *103*, 6104; (d) Blaudeau, J. P.; McGrath, M. P.; Curtiss, L. A.; Radom, L. *J. Chem. Phys.* **1997**, *107*, 5016; (e) McLean, A. D.; Chandler, G. S. *J Chem Phys* **1980**, *72*, 5639; (f) Krishnan, R.; Binkley, J. S.; Seeger, R.; Pople, J. A. *J. Chem. Phys.* **1980**, *72*, 650.

[S24] Knizia, G. *J. Chem. Theory Comput.* **2013**, *9*, 4834.
